# Cerebellar and frontal lobe alterations in pulmonary hypertension implicate an inflammatory lung vascular/brain axis

**DOI:** 10.1172/JCI195110

**Published:** 2026-11-02

**Authors:** Satoshi Okawa, Yinan Shi, Somanshu Banerjee, Yi Yin Tai, Jamal Nabhanizadeh, Stefan Guenther, Anisha N. Shah, Siyi Jiang, Timothy Kim, Yassmin Al Aaraj, Caroline Pidro, Metin Aytekin, Omolara Fatukasi, Nishita Kalepalli, Joseph Mettenburg, Timothy D. Girard, James T. Becker, Kathiravan Kaliyappan, Shanshan Song, Sandra Breuils-Bonnet, Steeve Provencher, Sébastien Bonnet, Theodore Huppert, Lloyd D. Harvey, Seyed Mehdi Nouraie, Rajkumar Savai, Dandan Sun, Soban Umar, Soni S. Pullamsetti, Stephen Y. Chan

**Affiliations:** 1 Center for Pulmonary Vascular Biology and Medicine, Pittsburgh Heart, Lung, and Blood Vascular Medicine Institute, Division of Cardiology, Department of Medicine;; 2 Department of Computational and Systems Biology; and; 3 McGowan Institute for Regenerative Medicine, University of Pittsburgh School of Medicine, Pittsburgh, Pennsylvania, USA.; 4 Department of Anesthesiology and Perioperative Medicine, Division of Molecular Medicine, University of California, Los Angeles, Los Angeles, California, USA.; 5 Institute for Lung Health, Department of Internal Medicine, German Center for Lung Research (DZL), Cardio-pulmonary Institute (CPI), Justus Liebig University, Giessen, Germany.; 6 Max Planck Institute for Heart and Lung Research, DZL, CPI, Bad Nauheim, Germany.; 7 Cellular and Molecular Pathology Graduate Program, University of Pittsburgh, Pittsburgh, Pennsylvania, USA.; 8 Heart and Vascular Institute, University of Pittsburgh Medical Center (UPMC), Pittsburgh, Pennsylvania, USA.; 9 Department of Critical Care Medicine,; 10 Department of Psychiatry,; 11 Department of Radiology, and; 12 Department of Neurology, University of Pittsburgh School of Medicine, Pittsburgh, Pennsylvania, USA.; 13 Pittsburgh Veteran’s Affairs Research Center, Pittsburgh, Pennsylvania, USA.; 14 PAH Research Group, Institut Universitaire de Cardiologie et Pneumologie de Québec Research Center, Department of Medicine, Université Laval, Quebec City, Québec, Canada.; 15 Department of Electrical and Computer Engineering, Swanson School of Engineering, University of Pittsburgh, Pittsburgh, Pennsylvania, USA.; 16 Division of Pulmonary, Allergy, and Critical Care Medicine, Department of Medicine, University of Pittsburgh School of Medicine, Pittsburgh, Pennsylvania, USA.

**Keywords:** Inflammation, Neuroscience, Pulmonology, Genetic risk factors, Neuroimaging, Transcriptomics

## Abstract

Connections between vascular and neurocognitive diseases are emerging but remain poorly defined. Cognitive impairment is increasingly recognized in pulmonary hypertension (PH), a disease traditionally viewed as limited to the heart and lungs. Given that inflammation is shared by PH and neurodegenerative diseases, we investigated its role in driving brain and cognitive deficits in PH. Using structural and functional MRI data from UK Biobank (*N* = 248 PH) and a prospective validation cohort, we identified reduced cerebellar white matter volume, thinner frontal gyri, and weakened cerebellar-frontal connectivity in patients with PH but not in other heart or lung diseases. These changes were accompanied by worsened executive function and processing speed. Olink proteomics and targeted genomics linked inflammatory plasma proteins and a PH-associated nuclear receptor coactivator 7 (NCOA7) single nucleotide polymorphism, rs11154337, to these deficits. PH cerebellum showed increased microglial activation and white matter derangement. In PH mice, Ncoa7 deficiency worsened cerebellar inflammation and atrophy. Microglial functional assays, cerebellar single-nucleus RNA sequencing, and spatial transcriptomic analyses further implicated Ncoa7-dependent lysosomal, inflammatory, and cell-specific mechanisms of cerebellar pathobiology. These findings identify NCOA7-linked inflammatory dysfunction in the cerebellar-frontal axis in PH, redefining this disease with neurologic consequences and thus offering neural targets for diagnostic, prognostic, and therapeutic development.

## Abstract 2

We identify mechanisms that drive cerebellar and cerebral neuroinflammation and cognitive dysfunction in pulmonary hypertension, thus offering neural targets for clinical development.

## Graphical abstract

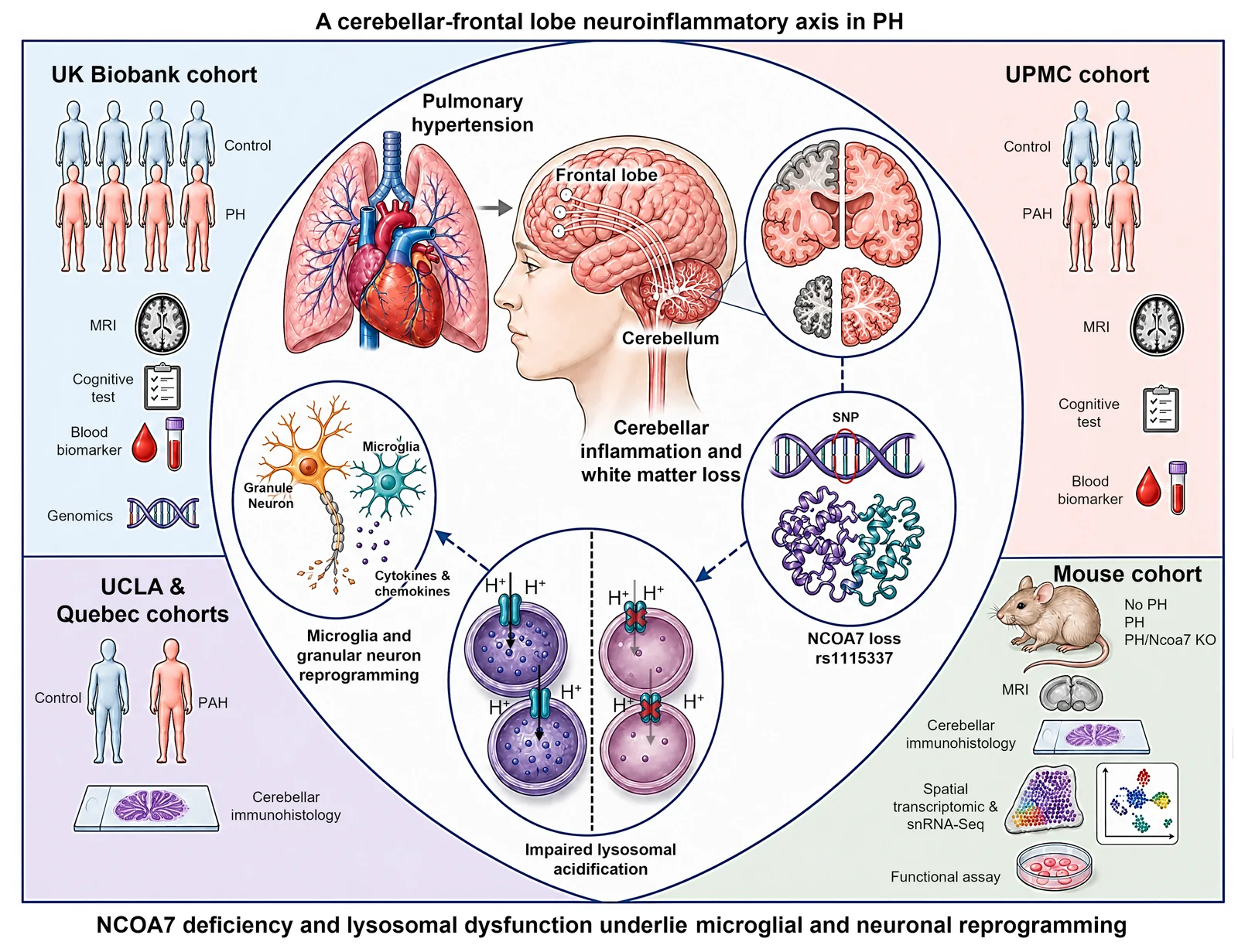

## Introduction

Physiologic and molecular links between vascular and neurodegenerative diseases are beginning to emerge, though they remain poorly defined (1). While the roles of autonomic and peripheral nervous system activity in regulating vascular tone are well established (2), understanding how cardiopulmonary diseases affect the central nervous system (CNS) is still in its early stages. Obesity (3) and vascular disease (4) are associated with the development of Alzheimer’s disease and dementia, even without stroke. Conversely, dementia may predict worse cardiovascular outcomes (5, 6). Structural brain changes and altered brain connectivity have been reported in heart failure and obstructive sleep apnea as well (7–9), yet the biology linking brain, heart, and lung vasculature is not defined.

Chronic inflammation may serve as a common pathway linking cardiovascular disease and CNS disorders. It plays a central role in peripheral vascular atherosclerosis (10, 11), and in pulmonary vascular disease (12), and regulates the progression of neurodegenerative diseases via microglial activation (13, 14). Genetic factors, such as the APOE4 allele, may further connect these disease domains by modulating inflammation. Notably, altered limbic activity has been linked to vascular inflammation and adverse outcomes in atherosclerosis (15, 16), suggesting the potential for brain-vascular inflammatory pathways. Whether a similar axis exists between the pulmonary vasculature and brain function is unclear.

Pulmonary hypertension (PH) is a progressive and often fatal pulmonary vascular disease where historic focus has been placed on the study of elevated pulmonary arterial pressure and right ventricular failure. The World Symposium on Pulmonary Hypertension (WSPH) classifies PH into 5 groups, including a particularly severe subtype known as pulmonary arterial hypertension (PAH, or group 1 PH) (17). Despite this classification, all 5 groups often exhibit overlapping pathophysiologic and histologic features, including inflammatory vascular pathophenotypes (18). Only recently have cognitive impairments, especially in executive function and memory, been described. Such impairments are observed in nearly half of the patients with PH in clinical cohorts (19–21), with cortical and subcortical brain alterations involving cerebral and cerebellar structures observed in a small study using brain MRI (22). While cerebellar disorders are traditionally associated with ataxia and coordination deficits, recent evidence indicates that the cerebellum also plays a critical role in learning (23) and executive function (24) by modulating frontal lobe activity (25–27).

Though cognitive decline in PH has been attributed to psychiatric symptoms (28, 29), cerebral hypoperfusion, or hypoxia (30), these explanations do not fully account for the observed deficits. Emerging data increasingly point to systemic inflammation as a driver of neurocognitive impairment in PH. Myeloid cell infiltration (31) and spinal cord inflammation (32) have been implicated in preclinical PH models. Central neuroinflammation, blood-brain barrier dysfunction, and cognitive decline have also been reported in monocrotaline-induced PAH rats (33–35). However, since monocrotaline is a known neuroinflammatory agent (35), its use in rodent models could confound the interpretation of PAH-related neuroinflammation. While there are emerging data reported in humans (36), there is a lack of direct evidence of neuroinflammation in human P(A)H and its potential impact on brain structure and function, highlighting a critical gap in understanding the neurological consequences of this disease in patients. In that vein, given the known links of lysosomal dysregulation in neurodegeneration (37, 38), a recently defined genetic and metabolic paradigm that links nuclear receptor coactivator 7–driven (NCOA7-driven) lysosomal biology to vascular inflammation and PH (39) could hold relevance to cognitive decline in this disease.

Here, we performed an integrated, multimodal investigation combining brain MRI, blood-based molecular profiling, and functional studies in mouse models to characterize the systemic nature of brain-lung interactions in PH. Our findings support a model in which systemic inflammation may contribute to brain dysfunction in PH, rather than reflecting only localized or secondary effects. Within this framework, lysosomal dysregulation via NCOA7 deficiency emerged as a candidate mechanism linking inflammatory PH biology to neurological manifestations. This work provides a foundation for redefining PH as a multiorgan inflammatory disorder with neurologic consequences.

## Results

### A multicohort human PH study of brain structure and function performed across discovery and validation cohorts.

The United Kingdom Biobank (UKBB) is a large-scale biomedical repository of deidentified data from 502,367 participants, generally representing mild-to-moderate prevalent disease states rather than severe illness (40, 41). It includes approximately 50,000 individuals with brain MRI from the initial imaging visit. Within this population, a PH cohort (*n* = 248) was defined using combinations of ICD-9/ICD-10 and OPCS codes established in prior studies (Figure 1A and Supplemental Table 1; supplemental material available online with this article; https://doi.org/10.1172/JCI195110DS1; Supplemental Methods). To improve case ascertainment beyond ICD coding alone, we applied a prior administrative data approach (42) that incorporated PH diagnosis codes with associated diagnoses and/or PAH-specific vasodilator use. This strategy increased confidence in identifying PH or PAH cases across multiple diagnostic and treatment domains, including connective tissue disease, left heart disease (LHD), and pulmonary vasodilator prescriptions. The resulting cohort represented a PH population spanning WSPH groups, in which inflammatory phenotypes have been broadly described.

The UKBB PH cohort had a median age of 68 years (IQR 61, 73); 44% were female, 41% had a college degree, and 50% had ever smoked. Comorbidities included essential hypertension (26.4%), ischemic or hemorrhagic stroke (1.6%), and diabetes with or without complications (7.5%), with a median BMI of 27.0 (IQR 25.18, 30.62) (Supplemental Table 2). The median time from PH diagnosis to MRI was 7.2 years (IQR 3, 13). Although precise WSPH group assignment was not possible in UKBB, prior ICD diagnoses of LHD, chronic obstructive pulmonary disease (COPD), or interstitial lung disease (ILD) were used to identify likely group 2–3 PH–related disease backgrounds. The blood sampling–MRI interval was similar between PH patients and controls [9.71 years (IQR 8.2, 10.9) vs. 9.34 years (IQR 7.75, 10.53)]. Notably, only 1 participant had a documented erectile dysfunction diagnosis (ICD-10: N48.4), suggesting that any vasodilator exposure in this group most likely reflects PAH-directed therapy rather than treatment for erectile dysfunction.

The UKBB control cohort (*n* = 11,656) was derived by excluding individuals with physician-diagnosed chronic disease, PH, chronic cardiopulmonary diseases listed in Figure 1C, or ICD codes indicating chronic infection or inflammation (Figure 1B and Supplemental Figure 1). The remaining cohort represented generally healthy controls with no known chronic disease and low prevalence of substance use disorder diagnoses (2%). Controls had a median age of 63 years (IQR 57, 69), 54% were female, 47% had a college degree, 51% had ever smoked, and median BMI was 25.5 (IQR 23.1, 27.6) (Supplemental Table 2A). Because more than 95% of both PH and control cohorts were White British (UKBB Data-Field 21000), individuals of other ethnic backgrounds were excluded to reduce population stratification and potential confounding.

The single-center, prospectively recruited UPMC validation cohort included 10 patients with PAH and 10 age-, sex-, and education-matched non-PAH healthy controls for T1/T2-weighted MRI and resting-state functional MRI comparisons (Supplemental Figure 2 and Supplemental Table 3). This focused independent validation cohort prioritized clinical homogeneity of idiopathic PAH over sample size, with stringent eligibility criteria selecting mild-to-moderate disease severity (NYHA Functional Class II–III), preserved hemodynamics, and MRI compatibility. Participants were European Caucasian and predominantly female (80%), with similar median age and education between PAH and controls (40 vs. 39 years; 15 vs. 16 years). No participants carried a diagnosis or were treated for erectile dysfunction. The median time from PAH diagnosis to MRI was 3.7 years (IQR 2.4, 7.2), compared with 7.2 years in UKBB (Supplemental Table 2B). In contrast with the >9-year blood sampling–MRI interval in UKBB, blood draw and MRI were nearly contemporaneous in UPMC: 8 of 10 PAH patients and 7 of 10 controls had same-day sampling and imaging, with only minor deviations in the remaining participants. Thus, UKBB and UPMC provided complementary information on PH-associated brain changes and their blood biomarker associations across different sampling intervals.

### Specific structural and functional connectivity alterations observed in PH/PAH across the left cerebellum/superior frontal gyrus axis.

To identify brain regions affected structurally and functionally by PH, we analyzed T1-weighted MRI (T1w-MRI) and resting-state functional MRI (rfMRI) data using covariate-adjusted ordinary lease squares (OLS) regression, with Benjamini-Hochberg correction for multiple comparisons (Supplemental Methods and Supplemental Table 2). Among 424 T1w-MRI–derived phenotypes, 5 showed significant reductions in the UKBB PH cohort compared with healthy controls: left superior frontal gyrus thickness (L-SFG; –2.08%, Padj = 0.035), left precentral gyrus thickness (L-PG; –2.55%, Padj = 0.035), left paracentral lobule thickness (L-PL; –2.83%, Padj = 0.035), right caudal middle frontal gyrus thickness (R-CMF; –1.83%, Padj = 0.039), and left cerebellar white matter volume (L-CRBL-WM; –4.39%, Padj = 0.049) (Figure 2A and Supplemental Table 4).

To assess functional correlates of these structural deficits, we quantified resting-state functional connectivity among the affected regions by rfMRI. Because blood oxygen level–dependent signal generally reflects neuronal postsynaptic activity (43–45), the entire left cerebellum, rather than only cerebellar white matter, was used for connectivity analysis. PH was associated with significantly reduced L-CRBL–L-SFG connectivity (AUC = –22.8%, Padj = 0.03) (Figure 2, A and B, and Supplemental Table 4), along with reduced covariate-adjusted L-CRBL-WM volume and L-SFG thickness (Figure 2, C and D, and Supplemental Table 4). White matter hyperintensity was also increased in PH (5.6%, Padj = 0.046), including in the cerebellum, consistent with cerebellar white matter injury (Figure 2, E and F). Global connectivity analysis also showed reduced betweenness centrality in L-CRBL and the L-CRBL–L-SFG connection, indicating diminished network hub function and information flow in PH. Specifically, PH was associated with reduced L-CRBL regional betweenness centrality (AUC = –13.8%, *P* = 0.003) and lower L-CRBL–L-SFG edge betweenness centrality (AUC = –10.9%, *P* = 0.025) (Figure 2, G and H).

Reduced right ventricular (RV) ejection fraction (<45%) was observed in only 16 of 232 PH participants with cardiac MRI data (6.9%), consistent with the mild-to-moderate disease severity represented in UKBB (40, 41). L-CRBL–L-SFG abnormalities were not significantly associated with RV systolic volume, RV dilation, or overall cardiac MRI-derived RV function (Supplemental Table 5). Similarly, no association was observed with SNPs carrying known genome-wide associations with PAH risk (46), including rs10103692, rs2856830, rs13266183, or available linked tag SNPs (*R*^2^ > 0.8) in UKBB (Supplemental Table 6). Pathogenic BMPR2 mutations were too infrequent for meaningful association analysis.

Validation analysis in the UPMC PAH cohort focused on brain regions significantly altered in the UKBB PH cohort. UPMC patients with PAH showed replicated reductions in L-SFG thickness (–3.9%, *P* = 0.005), L-CRBL-WM volume (–0.15%, *P* = 0.048), and L-CRBL–L-SFG functional connectivity (AUC = –39.4%, *P* = 0.017) (Figure 3, A–D, and Supplemental Table 7). Because all UPMC patients with PAH were receiving PAH-directed vasodilator therapy, treatment effects could not be isolated within this cohort, and future longitudinal studies of treatment-naive patients with PAH before and after therapy initiation will be needed. Nonetheless, replication of these abnormalities in a clinically phenotyped PAH cohort supports the interpretation that they are not explained by vasodilator exposure alone. Together, these findings demonstrate consistent structural and functional deficits within the L-CRBL–L-SFG axis across discovery and validation cohorts, spanning median diagnosis-to-MRI intervals of 3.7–9.7 years.

We next tested whether cognitive function was reduced in PH. In UKBB, all patients with PH from the initial assessment visit were analyzed, including those without MRI. PH was associated with impaired fluid intelligence, reflecting abstract reasoning, problem-solving, and working memory (Data-Field 20016; β = –0.222, Padj = 0.037), and slower matching performance, reflecting visual attention and processing speed (Data-Field 20023; β = 5.705, Padj = 0.046) (Supplemental Table 8). In the UPMC cohort, NIH Toolbox testing showed that patients with PAH performed worse on the Flanker inhibition test, reflecting interference control and executive function (paired 2-tailed *t* = –12.621, *P* = 0.009) (47, 48), and the Pattern Comparison test, reflecting processing speed and working memory (paired *t* = –12.962, *P* = 0.004) (Supplemental Table 8) (48). Thus, both cohorts showed PH-associated deficits in reasoning, executive function, and processing speed, consistent with prior reports of cognitive impairment in PAH and with the roles of frontal and cerebellar circuits in memory and executive function (21, 25, 49).

### Deficits in the L-CRBL–L-SFG axis are specific for PH.

We next assessed whether L-CRBL–L-SFG deficits could be explained by hemodynamic compromise or hypoxia. In UKBB, available cardiac and blood pressure measures showed only minimal group differences despite some statistically significant findings (Supplemental Table 2A). LV cardiac output, overall cardiac output, and ventricular rate were only modestly elevated in PH, whereas central systolic blood pressure did not differ significantly. These values remained within ranges expected for relatively mild-to-moderate disease and were unlikely to explain the observed brain abnormalities. Similar findings were observed in the UPMC validation cohort, where pulse oximetry values were largely normal (92%–99%) and elevated pulmonary arterial systolic pressures confirmed PAH without evidence of sustained hypoxemia or overt hemodynamic compromise (Supplemental Table 2B and Supplemental Table 3). Together, these physiologic profiles suggest that PH-associated L-CRBL–L-SFG abnormalities are not readily explained by hypoxia, systemic hypotension, or impaired cardiac output alone.

Cardiac hemodynamic disturbances, including reduced cardiac output or systemic hypotension from therapeutic vasodilators, can cause cerebral hypoperfusion and affect brain structure and function. Because PH therapy has also been linked to cognitive changes (20), we examined whether oral vasodilator treatment (UOVT) might contribute to the observed brain alterations. UKBB medication records do not distinguish vasodilator indication or continuous versus intermittent use; therefore, we evaluated a non-PH comparator cohort defined by UOVT exposure alone. Although this cohort was entirely male, it was not defined solely by erectile dysfunction and included diagnoses compatible with other vasodilator indications, including Raynaud’s-related, autoimmune, or vascular conditions — suggesting that vasodilators could have been prescribed for either intermittent or chronic use. The non-PH UOVT cohort showed no significant changes in brain volume or functional connectivity (Figure 4A and Supplemental Table 9), arguing against generalized vasodilator exposure alone as a sufficient explanation for the L-CRBL–L-SFG abnormalities in PH or PAH.

We next identified non-PH UKBB comparator cohorts with comorbidities associated with major PH groups to test whether the PH brain phenotype was reproduced by related conditions (Figure 1C and Supplemental Table 9). These included group 1–associated conditions, including congenital heart disease (CHD), autoimmune diseases, and infection by HIV (HIV); group 2–associated LHD; group 3–associated COPD, ILD, and sleep disordered breathing; and group 5–associated metabolic disease and renal failure. Deep venous thrombosis and pulmonary embolism (group 4) and hematologic disorders (group 5) were considered but excluded because PH-free sample sizes were too small (*n* < 10), primarily because many such individuals also carried a PH diagnosis.

Seven of 9 comparator cohorts showed no significant brain alterations (Figure 4A and Supplemental Table 10). Significant structural changes were observed only in HIV and COPD. HIV was associated with reduced volumes of bilateral ventral diencephalon, right thalamus, pallidum, and hippocampus (Figure 4B and Supplemental Table 10). COPD showed broader reductions involving bilateral ventral diencephalon, thalami, hippocampi, cerebellar white matter, left cerebellum, left amygdala, and brain stem, with several reduced functional connectivity interactions among these regions (Figure 4C and Supplemental Table 10). However, neither COPD nor other major cardiopulmonary comparator cohorts, including LHD and ILD, reproduced the combined L-CRBL–L-SFG structural and functional deficiency pattern observed in PH. To further assess potential confounding by LHD or chronic hypoxic lung disease within the PH cohort, we adjusted for prior LHD, COPD, or ILD diagnosed ≥6 months before PH diagnosis (28/248, 11.3%). This adjustment did not materially change the PH associations, and L-CRBL–L-SFG abnormalities remained significant with the same direction of effect. A broader definition including LHD/COPD/ILD diagnoses any time on or before PH diagnosis (42/248, 16.9%) yielded consistent results: the L-CRBL-WM volume association became borderline (Padj = 0.053) with essentially unchanged effect size (β = −408.73 vs. −408.52), while the remaining L-CRBL–L-SFG structural and functional associations were preserved. These findings support that the PH cerebellar–frontal phenotype was not reproduced in the comparator cohorts tested and was not explained by preceding LHD, COPD, or ILD alone.

Because patients with PH may carry potentially confounding cardiovascular, endocrine, and neuropsychiatric diagnoses, including hypertension, diabetes, anxiety, and depression (50), we performed the same structural and functional connectivity analyses in non-PH UKBB cohorts with these conditions (Figure 1C and Supplemental Table 9). Significant brain volume changes were observed in hypertensive disorders (HD), diabetes mellitus (DM), and depression disorders (DD). HD showed increased right choroid plexus volume and reduced right accumbens volume (Figure 5A and Supplemental Table 10). DM showed broader structural alterations, including increased bilateral choroid plexus volume and reduced volumes or thicknesses across ventral diencephalon, parietal, frontal, thalamic, and related cortical regions, with multiple reduced functional connectivity interactions among these regions (Figure 5B and Supplemental Table 10). DD was associated with reduced bilateral hippocampal volumes (Figure 5C and Supplemental Table 10).

Thus, the deficit in the L-CRBL–L-SFG axis was not reproduced in the comparator cohorts tested and was not associated with other comorbidities linked to PH or with vasodilator use independent of PH.

### L-CRBL–L-SFG axis deficiencies are associated with plasma biomarkers of inflammation.

We next examined plasma biomarker associations with L-CRBL–L-SFG deficiencies to identify molecular pathways potentially underlying these brain alterations. Analyses were performed within the PH cohort to capture PH-specific biomarker–brain relationships rather than case-control differences. Among 30 UKBB blood biochemical markers, 11 were significantly associated with L-CRBL-WM volume or L-SFG thickness (Figure 6A and Supplemental Table 11). IGF-1, testosterone, and hemoglobin A1c (HbA1c) were associated with both regions; urea, cystatin C, urate, SHBG, creatinine, and albumin were associated only with L-CRBL-WM volume; and vitamin D and C-reactive protein (CRP) were associated only with L-SFG thickness. Importantly, 10 of these 11 markers have known links to chronic vascular or neurologic inflammation, including HbA1c and CRP, which have been associated with mild cognitive impairment (MCI) in other contexts (51–53). To test whether these associations reflected metabolic dysregulation alone, we repeated the analysis in non-PH DM participants. In contrast with PH, neither HbA1c nor testosterone was significantly associated with L-CRBL-WM volume or L-SFG thickness, suggesting that these biomarker–brain relationships are not explained by diabetes-related metabolic variation alone.

We next analyzed UKBB plasma proteomic data from the Olink platform. Among 2,923 circulating proteins, multivariate analysis identified 30 proteins associated with L-CRBL-WM volume, 11 with L-SFG thickness, and 13 with L-CRBL–L-SFG connectivity (Supplemental Figure 3 and Supplemental Table 12). Gene Ontology Biological Processes (GOBP) enrichment of inversely associated proteins, representing higher expression with greater L-CRBL–L-SFG deficiency, revealed immune-response pathways across all 3 analyses (Figure 6B and Supplemental Table 13). Notably, top enriched categories included microglial activation and neuroinflammatory responses, with GRN, PTPRC, and CX3CL1 highlighting abnormal microglial states as part of the L-CRBL–L-SFG inflammatory signature. When inversely and positively associated proteins were pooled, additional pathways related to chemokine response, cytokine signaling, cell proliferation, immune-cell chemotaxis, and Wnt signaling were identified (Supplemental Table 13). To assess whether hypoxic mechanisms contributed to these associations, we analyzed 66 curated hypoxia-related Olink proteins (Supplemental Table 14); none showed significant association, contrasting with the enrichment of immune and inflammatory proteins.

Although precise WSPH classification was not feasible in UKBB, we examined whether plasma biomarker associations were detectable within PH strata, including the more prevalent LHD-associated PH subgroup. PH participants were stratified by LHD diagnosis before or at PH diagnosis and analyzed using identical regression frameworks. Routine biochemical markers, including urea, cystatin C, creatinine, urate, HbA1c, CRP, lipid-related markers, and vitamin D, showed significant associations with PH in the same direction in both LHD-associated and non-LHD PH strata (Supplemental Table 15). Similarly, 459 Olink proteins were significantly associated with PH in both strata, supporting shared inflammatory biomarker patterns across LHD-associated and non-LHD PH contexts (Supplemental Table 15).

To independently validate key inflammatory proteins, we selected significant Olink proteins with commercial ELISA kits (CFD, GRN, C1QBP, and CX3CL1) and measured plasma levels by ELISA in the UPMC cohort (Figure 6, C–F). CFD (*P* = 0.0232), GRN (*P* = 0.0082), and CX3CL1 (*P* = 0.0247) were significantly elevated in PAH compared with controls, whereas C1QBP was not significantly different (*P* = 0.4904). Together, UKBB and UPMC data support a robust circulating inflammatory signature across PH contexts, including PAH and LHD-associated PH, despite differences in blood sampling timing relative to brain MRI. These associations suggest that persistent inflammatory signaling may contribute to the structural and functional brain deficiencies observed in PH.

### Intronic NCOA7 SNP rs11154337 is associated with the L-CRBL–L-SFG connectivity strength.

We recently identified the NCOA7–lysosomal–oxysterol axis as a regulator of pulmonary vascular inflammation and mortality in PAH, with the rs11154337 G-allele conferring protection by increasing NCOA7 expression and reducing oxysterol-induced inflammation (39). Because lysosomal dysfunction and oxysterol production are also linked to neuroinflammation and neurodegeneration (37, 38), we hypothesized that NCOA7 deficiency may represent a shared inflammatory mechanism across pulmonary vascular and cerebellar compartments. In UKBB patients with PH, proteins negatively associated with rs11154337_G showed pathway-level enrichment for systemic inflammatory programs, including complement activation, classical complement activation, acute-phase response, and lymphocyte-mediated immunity, whereas regulation of mitochondrial membrane potential was enriched in the positive direction (Figure 6G). Cardiopulmonary comparator groups, including LHD, COPD, and ILD, did not show a fully comparable PH-like inflammatory pattern (Supplemental Table 16), and only in DM were other inflammatory pathways significantly associated with rs11154337_G. Taken together, these data support an association between rs11154337 and systemic immune/inflammatory biology that is primarily represented in PH.

We next tested whether rs11154337 was associated with preservation of the L-CRBL–L-SFG axis. In UKBB patients with PH stratified by genotype [C/C (*n* = 40), G/C (*n* = 94), G/G (*n* = 42)], the G-allele was not significantly associated with L-CRBL-WM volume or L-SFG size but was associated with improved L-CRBL–L-SFG connectivity under an additive model (β = 0.020, *P* = 0.035), with connectivity increasing stepwise by G-allele dosage (Figure 6H). This association was not observed in comparator populations, except for L-CRBL-WM volume in a small CHD cohort with limited power (*n* = 22; Supplemental Table 17). Together, these Olink–genotype and imaging–genotype findings link NCOA7-related inflammatory biology to preservation of cerebellar–frontal connectivity in PH.

### PAH cerebellar tissue shows increased microglial abundance and multicellular induction of NCOA7.

To define inflammatory changes and NCOA7 involvement in PAH cerebellum, we performed immunofluorescence staining of postmortem cerebellar tissue from patients with PAH and healthy controls (Supplemental Table 18). Ionized calcium-binding adapter molecule 1 (IBA1) staining showed significantly increased IBA1^+^ microglia in both molecular and granular layers of PAH cerebellum (Figure 7, A–E). Co-immunofluorescence further showed increased NCOA7 signal within IBA1^+^ microglia in both layers (Figure 7, F–I), as well as in CALBINDIN^+^ Purkinje neurons (Figure 7, J and K) and NEUN^+^ granule neurons (Figure 7, L and M), but not in PARVALBUMIN^+^ molecular layer neurons (Supplemental Figure 4, A and B). NCOA7 was also expressed in CD31^+^ endothelial cells but was not detected at high levels in GFAP^+^ astrocytes or MBP^+^ ODCs (Supplemental Figure 4, A–E). Consistent with increased sterol hydroxylation and downstream vascular inflammation controlled by NCOA7 in PAH (39), CH25H was also significantly upregulated in PAH cerebellum (Figure 7, N and O). Together, these findings indicate that PAH cerebellum is characterized by increased microglial abundance and cell type–selective NCOA7 protein induction across microglial and neuronal compartments. Such upregulation is consistent with our observations in human diseased pulmonary vasculature in PAH (39), where increased NCOA7 ensures appropriate lysosomal function and prevents overexuberant inflammation. This dependence on inducible levels of NCOA7 thus identified these cells as those most likely to be vulnerable in contexts of NCOA7 deficiency.

### An inflammatory PH mouse model recapitulates cerebellar volume loss, microglial expansion, and multicellular NCOA7 induction.

To determine whether the cerebellar abnormalities observed in human PAH were reproduced in an experimental PH model, we used an Il6-Tg mouse model that recapitulates inflammatory PH without confounding from direct ischemic or toxic brain injury, such as monocrotaline exposure (54). Co-immunofluorescence showed increased NCOA7 signal within IBA1^+^ microglia in both layers (Figure 8, A–D), as well as in CALBINDIN^+^ Purkinje neurons (Figure 8, E and F), NEUN^+^ granule neurons (Figure 8, G and H), and PARVALBUMIN^+^ molecular layer neurons (Figure 8, I and J). Together, these findings indicate that the Il6-Tg PH model recapitulates cell type–selective NCOA7 induction across microglial and neuronal compartments observed in human PAH cerebellum.

Next, to investigate mechanisms controlling cerebellar pathology in PH, we used an Ncoa7-knockout Il6 mouse model (Il6-Tg Ncoa7^–/–^). Because full-length Ncoa7 deletion is constitutive, we assessed whether its loss alone caused developmental brain abnormalities. Prior work reported normal brain development in full-length Ncoa7^–/–^ mice (55), and our MRI analyses similarly showed no reduction in total brain volume (1.8%, *P* = 0.765) or cerebellar volume (2.1%, *P* = 0.636) compared with WT mice (Supplemental Figure 5, A and B). We then examined Il6-Tg Ncoa7^–/–^ mice to determine whether Ncoa7 deficiency exacerbated cerebellar pathology in PH (39). Ex vivo T2w-MRI with NeAt atlas-based parcellation (56, 57), adjusted for age and total brain volume, showed reduced cerebellar volume in Il6-Tg mice compared with WT controls (–4.9%, Padj = 0.048; Figure 9A), with a further reduction in Il6-Tg Ncoa7^–/–^ mice compared with Il6-Tg mice (–7.1%, Padj = 0.029; Figure 9A), indicating an interaction between inflammatory PH and Ncoa7 deficiency rather than a primary effect of Ncoa7 loss. Il6-Tg Ncoa7^–/–^ mice also showed greater IBA1 expression in the molecular and granular layers (Figure 9, B–D) compared with Il6-Tg mice, together with increased CH25H expression as a marker of inflammatory activation (Figure 9, E and F). These findings indicate that Ncoa7 modulates cerebellar neuroinflammation and volume loss in PH. To exclude the possibility that Ncoa7 deletion alone causes baseline cardiopulmonary abnormalities that secondarily affect brain structure, we assessed WT and Ncoa7^–/–^ mice by echocardiography and right-heart catheterization. No significant differences were observed in cardiac structure, function, or pulmonary pressures (Supplemental Table 19), indicating that Ncoa7 deletion alone does not induce baseline cardiopulmonary pathology.

### NCOA7 regulates microglial lysosomal function and inflammatory activation.

To define how NCOA7 deficiency may contribute to neuroinflammation, we built on prior evidence that NCOA7 regulates lysosomal acidification and CH25H-dependent oxysterol–bile acid inflammatory signaling (39). Given our finding of cerebellar microglial NCOA7 upregulation in human and mouse PAH models (Figure 7, F–I, and Figure 8, A–D), we focused on microglia as a candidate effector linking NCOA7 deficiency to neuroinflammation. In the HMC3 cultured human microglial cell line, shRNA-mediated NCOA7 knockdown was confirmed by immunoblotting (Figure 10, A and B) and caused lysosomal dysfunction, including reduced lysosomal acidification, diminished cathepsin D activity, and increased intracellular lipid accumulation (Figure 10, C–K). NCOA7-deficient microglia also showed impaired phagocytosis of pHrodo-labeled synaptosomes (Figure 10, L–N) and increased expression of inflammatory cytokines IL1A, IL1B, IL6, and TNFA and chemokines CCL2 and CXCL10 (Figure 10, O–T). These lysosomal and inflammatory phenotypes were independently reproduced using siRNA-mediated NCOA7 silencing (Supplemental Figure 6). Together, these findings identify NCOA7 as a regulator of microglial lysosomal integrity, phagocytic function, and basal inflammatory activation, providing a mechanistic link between NCOA7 deficiency and PH-associated cerebellar neuroinflammation.

### Single-nucleus and spatial transcriptomics reveal region-specific cerebellar vulnerability to PH and Ncoa7 loss.

To investigate the cellular and molecular basis of PH-associated cerebellar alterations and the contribution of Ncoa7 deficiency, we performed single-nucleus RNA-seq (snRNA-seq) of cerebellar tissue from WT, Il6-Tg, and Il6-Tg Ncoa7^–/–^ mice. After quality control and reference-based annotation (58), 248,660 nuclei were retained, resolving major cerebellar neuronal, glial, immune, vascular, ventricular, and stromal populations (Figure 11A). Granule neurons and MLIs were further subdivided into Granule_main/Granule_other and MLI1/MLI2, respectively, with cell type assignments supported by canonical marker gene enrichment from the reference cerebellar atlas (Supplemental Figure 7A). No significant changes in cell type composition were detected across the conditions (Supplemental Table 20). Ncoa7 nuclear transcripts were broadly but lowly detected across diverse cell types (Figure 11B and Supplemental Figure 7, B–I). However, unlike quantitative co-immunofluorescence (Figure 8), nuclear Ncoa7 transcript abundance was not significantly increased in PH, likely reflecting the inability of single-nucleus sequencing to distinguish changes in inflammation-responsive isoform usage and cytoplasmic RNA processing previously linked to increased NCOA7 protein expression (39, 59). Thus, snRNA-seq provided a cell type map of Ncoa7 transcript detection and a cerebellar reference for downstream transcriptional reprogramming and spatial transcriptomics deconvolution.

We then performed cell type–specific differential expression analyses for Il6-Tg versus WT and Il6-Tg Ncoa7^–/–^ versus Il6-Tg, with complete differentially expressed gene (DEG) and gene set enrichment analysis (GSEA) results provided in Supplemental Tables 21 and 22. We focused on DEGs changing in the same direction across both comparisons (stepwise DEGs) in Ncoa7-expressing cell types supported by both snRNA-seq and immunofluorescence: granule neurons, MLIs, Purkinje cells, and microglia (Supplemental Tables 23 and 24). In Granule_main neurons, stepwise increased DEGs were enriched in pathways of mitochondrial RNA metabolism/processing, synapse organization, axon extension, lipid/glycerolipid metabolism, and intracellular protein transport, while decreased DEGs involved dendritic spine morphogenesis, neuron projection/axon development, and synaptic regulation (Figure 11C). Kyoto Encyclopedia of Genes and Genomes analysis additionally identified circadian rhythm among stepwise decreased DEGs (Supplemental Figure 7J). Together, these findings indicate progressive disruption of granule neuron neurite/synaptic regulatory programs in PH, exacerbated with Ncoa7 deficiency, accompanied by metabolic, mitochondrial RNA–processing, and synaptic responses, consistent with the known role of NCOA7 in sterol and lipid regulations (39). MLI1 showed more restricted enrichment for glutamate receptor signaling and chemical synaptic transmission, whereas Purkinje cell and microglial analyses were limited by lower cell recovery and did not yield significant stepwise DEG-GSEA enrichment (Supplemental Table 24).

Next, spatial transcriptomic analyses were conducted to map PH- and Ncoa7 deficiency–associated programs within defined cerebellar microenvironments. This approach enabled transcriptional changes to be localized to anatomical domains enriched for less abundant populations, including Purkinje neurons and microglia (60). Unbiased clustering identified 6 anatomically consistent cerebellar regions (Figure 12A), and integration with the snRNA-seq reference supported these annotations by defining dominant cell type composition and selectively enriched signatures across clusters (Supplemental Figure 7, K and L). Cluster 0 (C0) corresponded to the granule cell layer, cluster 1 (C1) to a molecular layer/interneuron-enriched region, cluster 2 (C2) to the Purkinje cell layer, cluster 3 (C3) to ODC-enriched white matter, cluster 4 (C4) to a choroid plexus-associated ventricular region, and cluster 5 (C5) to a fourth ventricle–adjacent periventricular white matter/medullary velum border. No significant changes in predicted cell fraction were detected (Supplemental Table 25).

We next performed most enriched cell type–targeted DEG-GSEA within each spatial cluster while adjusting for other local cell type signals (Supplemental Tables 26 and 27), enabling identification of programs associated with the principal anatomical/cellular signature of each cerebellar region. In neuron-enriched cortical layers (i.e., C0–2), Il6-Tg versus WT showed upregulated apoptosis/cell death, oxidative stress, lipid/metabolic stress, synaptic/neuronal projection, and proteostasis-related programs, together with downregulated mitochondrial respiration/ATP metabolism, ion transport/excitability, calcium signaling, and neuronal/synaptic regulatory pathways (Figure 12B). Such enrichment was also observed in pathways highlighted by snRNA-seq analysis alone in Granule_main neurons (Figure 11C), suggesting the consistency between the single nuclear and spatial datasets. In Il6-Tg Ncoa7^–/–^ versus Il6-Tg, these changes became broader and with increased lipid/stress, inflammatory, and apoptotic pathway engagement across C0–C2 (Figure 12C). Together, these findings support the MRI and histologic data by showing that PH is associated with anatomically distributed cerebellar transcriptional remodeling, while Ncoa7 deficiency further shifts neuron-enriched compartments toward metabolic and neuronal dysfunction with amplified inflammatory and cell death signaling.

Disease-associated remodeling extended beyond neuron-enriched cortical layers. In particular, under Il6-Tg conditions, C3 showed downregulation of axonal and myelin homeostatic programs, including axonogenesis, axon development, and regulation of supramolecular fiber organization (Figure 12B and Supplemental Table 27). Altered regulation of neuronal apoptotic processes further suggested cell death–associated vulnerability within this compartment. In Il6-Tg Ncoa7^–/–^ mice, this white matter–associated disruption was more pronounced, with reduced aerobic respiration, oxidative phosphorylation, and proteasomal protein catabolism, along with increased apoptotic programs (Figure 12C and Supplemental Table 27). These findings support the notion that chronic neuroinflammation promotes white matter injury and brain volume loss by disrupting glial survival, myelin maintenance, axonal integrity, and lysosomal-autophagic protein/lipid handling (61–65). Together, these data provide a molecular context for MRI-detected cerebellar white matter volume loss in PH, potentially via disrupted axonal and myelin support.

Finally, we examined microglia-associated signals within these spatial domains beyond those captured by the principal anatomical or cellular signatures. Microglia-targeted DEG-GSEA was performed within each spatial cluster after removing pathways enriched in the corresponding most enriched cell type–targeted analysis (Supplemental Tables 28 and 29). In Il6-Tg versus WT, retained microglia-associated programs involved cytokine production, protein-complex organization, protein transport, ubiquitin/proteasome-mediated catabolism, ERAD/unfolded protein response, calcium homeostasis, and synaptic signaling-related processes (Figure 12D). Il6-Tg Ncoa7^–/–^ versus Il6-Tg further involved IL-6 signaling, lysosomal targeting, autophagy, ER-to-Golgi/vesicle transport, oxidative and chemical stress responses, phospholipid/membrane-raft biology, and apoptosis (Figure 12E). These pathways converged on functional axes identified by NCOA7 knockdown in vitro, including lysosomal/autophagic regulation, lipid and membrane metabolism, proteostasis/ER stress, oxidative stress, inflammatory signaling, and synaptic-material handling (Figure 10). Based on this cluster-specific distribution, Ncoa7 deficiency promoted a regionally dependent microglial stress response program, rather than a globally uniform cerebellar response. Taken together, across PH and exacerbated by Ncoa7 deficiency, these findings defined a landscape of cell type–specific neuronal and microglial transcriptional reprogramming involving lysosomal and inflammatory cerebellar pathobiology.

## Discussion

This study identified reproducible structural and functional brain abnormalities in PH and linked them to systemic inflammation across imaging, molecular, and animal model platforms. We observed reduced volume and functional connectivity within the left cerebellar–left superior frontal gyrus (L-CRBL–L-SFG) axis, a finding that was specific to PH and accompanied by cognitive and executive function alterations. Plasma proteomics further associated these brain changes with neuroinflammatory and microglia-related markers, as well as a pro-inflammatory NCOA7 SNP independently linked to PH severity (39). Postmortem analysis and PH mouse models showed cerebellar microglial activation and structural deficits worsened by NCOA7 deficiency. Consistent with NCOA7- and lysosome-dependent dysfunction in cultured human microglia, PH-dependent and Ncoa7-driven global transcriptomic cerebellar reprogramming in mice was marked by microglial and neuronal inflammatory activation as well as neuronal cell death signaling, which correlated with white matter volume loss. Collectively, these findings support PH-associated cerebellar–frontal brain deficiencies linked to chronic neuroinflammation and NCOA7 dysfunction. More broadly, they provide translational evidence for considering PH as a multiorgan inflammatory disorder with neurologic consequences and potential therapeutic targets. Although the UPMC validation cohort was modest in size, the consistent effect direction and anatomical specificity across cohorts and experimental models support biological robustness rather than cohort-specific artifacts.

These findings advance our understanding of neurocognitive deficits in PH by defining alterations in specific brain regions, particularly the cerebellum and superior frontal gyrus. Although UKBB PH analyses also showed structural changes in the precentral and caudal middle frontal gyri, only L-CRBL–L-SFG abnormalities were consistently observed across independent cohorts, supporting their generalizability. The basis for this left-sided vulnerability remains unclear, particularly because handedness did not differ between PH and control groups, and the selective susceptibility of these regions in PH also remains unknown. The cerebellum is classically involved in motor coordination and voluntary movement but is increasingly recognized for higher neurocognitive functions, including cognitive processing and emotional regulation (25–27). Its connectivity with the superior frontal gyrus is relevant to frontal lobe functions such as calculation, verbal memory, and abstract thinking (66). Reduced cerebellar–frontal connectivity has been reported in chronic pain (67), depression (68), and dementia (69). Together, these observations suggest that the L-CRBL–L-SFG abnormalities identified in PH may reflect engagement of broader inflammatory and neurovascular mechanisms that are relevant to cognitive dysfunction across disease contexts.

Beyond explaining cognitive dysfunction in PH, these brain alterations may also inform neural contributions to pulmonary vascular regulation. However, the relationship between frontal or cerebellar activity and vascular control in health or disease remains poorly understood. Although higher cortical and subcortical regions can influence autonomic function (68, 69), whether cerebellar–frontal network alterations affect pulmonary vascular tone, remodeling, or hemodynamic progression in PH is unknown. These findings lay the groundwork for future studies on the development, hemodynamic relevance, cognitive impact, and potential reversibility of brain alterations in PH.

Our findings implicate chronic inflammatory processes in alterations of the L-CRBL–L-SFG axis. Importantly, they argue against altered cardiac output, systemic hypotension, comorbid conditions, vasodilator exposure, or chronic hypoxia as primary explanations for these brain abnormalities. The absence of strong associations with cardiac output or systolic blood pressure likely reflects both the population-based UKBB cohort and the UPMC validation cohort’s exclusion of advanced hemodynamic compromise, enabling detection of neurocognitive and inflammatory changes before end-stage right-heart failure. Similarly, non-PH individuals receiving oral vasodilator therapy did not show comparable abnormalities, and PH mice showed cerebellar deficits without vasodilator exposure, arguing against treatment effects as the sole explanation. Although PAH-directed therapies may have CNS effects that cannot be fully isolated in treated patients, their reported antiinflammatory actions (70–72) would not readily explain the pro-inflammatory cerebellar phenotype observed here. Chronic hypoxic or ischemic injury also appears unlikely as a primary driver, given the lack of prolonged hypoxemic states and absence of hypoxia-related plasma proteomic enrichment (Figure 6), although longitudinal physiologic data will be needed to fully exclude hypoxic contributions. Rather, our data support a broader role for NCOA7 deficiency and rs11154337 across an inflammatory lung/brain axis, linking pulmonary vascular remodeling with cerebellar dysfunction and cognitive decline.

Indeed, when considering shared associations across structural brain and plasma biomarker indices in the UPMC PAH cohort as well as the LHD-associated PH subgroup, our collective findings converge on shared neurobiological mechanisms, apparently active across multiple forms of PH and potentially mediated by overlapping systemic or inflammatory pathways. The absence of comparable rs11154337 associations or PH-like inflammatory patterns in comparator diseases also suggests that the rs11154337–inflammation–L-CRBL–L-SFG relationship is not simply a generic consequence of systemic inflammation but may reflect PH-enriched biology and/or local neuroinflammatory modulation by NCOA7. In support of this possibility, NCOA7 (39) and its homolog OXR1 (73–77) have been implicated in vascular endothelial and cerebellar microglial biology — findings that correspond to our data showing NCOA7-dependent control of microglial lysosomal function, inflammation, and phagocytosis (Figure 10). Furthermore, the transcriptional and inflammatory reprogramming of murine cerebellar neuronal populations that display Ncoa7 upregulation in the setting of PH (Figures 11 and 12) offers intriguing cell type–specific and lysosomal-centric insights into the white matter volume loss observed in human populations (Figures 2 and 3). In addition, given the regionally specific microglial stress response program dictated by Ncoa7 deficiency (Figures 11 and 12), we speculate that such distinct inflammatory brain patterns as dictated by NCOA7 may explain the distinct inflammatory brain patterns reported in the limbic system in atherosclerosis (15, 16) and L-CRBL–L-SFG involvement observed here in PH. Finally, given links among inflammation, NCOA7-dependent oxysterol biology, and neurodegenerative disease, future studies should determine whether tau-related pathology or other dementia-associated mechanisms contribute to PH-associated brain lesions and the broader clinical connection between vascular disease and dementia (69).

From a translational perspective, the association of inflammatory plasma biomarkers with structural and functional brain alterations may inform new diagnostic and prognostic strategies. In PH, molecular and genetic biomarkers of risk or disease severity remain limited, and current standard-of-care assessments are still incomplete (78). Prior studies have linked elevated CRP to MCI (51–53), consistent with our observed negative association between CRP and L-SFG cortical thickness, further supporting a role for systemic inflammation in PH-related brain abnormalities. Because plasma sampling preceded brain MRI by several years in UKBB, these findings raise the possibility that inflammatory biomarkers may predict PH-related brain lesions much later in life rather than simply reflecting contemporaneous injury. Future longitudinal studies will be needed to define when these abnormalities emerge, whether they progress, and whether inflammatory biomarkers identify at-risk patients before overt neuroimaging changes. Integrating these markers with NCOA7-related biomarkers, including our previously developed NCOA7-dependent oxysterol/bile acid poly-metabolite severity score (79), may further improve risk prediction.

Our findings also open avenues for testing whether PH-associated neuroinflammatory changes are therapeutically reversible. First, it remains unclear whether current vasodilators or emerging antiremodeling therapies influence development of L-CRBL–L-SFG abnormalities, requiring better definition of lesion progression and persistence. Second, depending on whether neuroinflammation is driven by peripheral immune activation, resident microglia, or blood-brain barrier dysfunction, effective antiinflammatory strategies may not necessarily need to cross the blood-brain barrier. Third, our prior development of a pharmacologic NCOA7 activator (39) provides a platform to test whether NCOA7 activation can improve cerebellar neuroinflammation, cognitive dysfunction, and pulmonary vascular remodeling in PH. Finally, it remains unknown whether directed neurologic intervention could modify the hemodynamics of PH. If so, behavioral approaches that strengthen neurocognitive connectivity could be integrated into clinical trials (80). More invasive approaches, such as deep-brain stimulation, which has shown benefit in selected neurodegenerative and neuropsychiatric diseases (81), could eventually be explored, although such strategies remain speculative in PH.

Limitations are acknowledged and should prompt future studies. First, UKBB PH classification relied on ICD code–based algorithms, which limits diagnostic precision, particularly for PH subtypes. Although non-PH comparator cohorts with LHD, COPD, or ILD and covariate adjustment for preceding LHD/COPD/ILD argue against these comorbidities alone explaining the L-CRBL–L-SFG abnormalities, the lack of invasive hemodynamic data or expert physician adjudication precluded definitive PH subtype–specific imaging analyses. Second, although the UPMC validation cohort size was modest, it was constructed to minimize heterogeneity. Thus, the consistent effect direction and anatomical specificity across cohorts and experimental models support biological robustness rather than cohort-specific artifacts. Defining subtype-specific mechanisms and effect sizes will require larger cohort studies with invasive phenotyping. Third, plasma sampling preceded brain MRI by several years in UKBB, whereas UPMC sampling was nearly contemporaneous. The convergence of plasma–brain associations across these different intervals suggests that inflammatory biomarkers may persist over time and potentially predict later brain alterations, but longitudinal studies with repeated plasma sampling, MRI, and cognitive testing are needed to define temporal relationships. Fourth, variability in cognitive testing methods limits direct cross-cohort comparison. Fifth, the predominance of White participants in UKBB-derived cohorts limits generalizability to other racial populations. Future studies coupling quantitative indices of PH with diffusion MRI of cortico-cerebellar tracts and task-based fMRI would be ideal to define PH subtype–specific mechanisms, structural connectivity, and active network dysfunction.

Overall, by combining epidemiologic and molecular evidence across human data, histologic analyses, and rodent modeling, our work defines a neuroinflammatory component in PH, centered on a cerebellar–frontal lobe axis, with significant implications for the neurocognitive and systemic manifestations of this morbid disease.

## Methods

### Sex as a biological variable.

Men and women were included in human cohort analyses, and sex was incorporated for statistical adjustment. In PH mouse models, females tend to be significantly more resilient to inflammatory stimuli than males (82). Consequently, male mice tend to develop a more severe and phenotypically consistent pathophenotype of pulmonary vascular remodeling under inflammatory induction. Thus, to ensure a robust, reproducible disease phenotype, this study utilized a single-sex cohort of male mice. Studies are ongoing to define any sex-dependent effects on brain and cognitive function in PH mouse models.

### Statistics.

Animal sample sizes were calculated to detect at least a 25% difference between control and experimental groups with 80% power and a 10% SD. Patient sample sizes for molecular analyses were determined primarily by clinical availability. Case-control comparisons in UKBB and UPMC cohorts were performed using covariate-adjusted OLS regression, as specified in each subsection. UKBB medication records do not specify treatment indication; therefore, potential vasodilator confounding was evaluated using a dedicated oral vasodilator use cohort. As a consequence of PAH, vasodilator use, including PDE5 inhibitors, was not included as a PAH model covariate, to avoid overadjustment. Sensitivity analyses addressed LHD/COPD/ILD confounding by comparing PH-free comparator cohorts and by adjusting for these diagnoses within PH when they preceded PH diagnosis. Lifestyle-related covariates included smoking status, BMI, and education; substance use disorder diagnoses were uncommon in UKBB controls and absent in UPMC. Regression β-coefficients from adjusted OLS models were used as effect estimates. For visualization, adjusted outcome values were calculated as residuals with the group effect reintroduced. For Olink proteomics and blood biomarker analyses, Gaussian GLMs included standardized time offset between biospecimen collection and MRI acquisition; CIs and *P* values were estimated by bootstrapping. SNP associations were tested using additive genetic encoding and the same covariates as MRI models. Ex vivo mouse MRI used covariate-adjusted regression models with empirical *P* values estimated by permutation testing followed by Holm’s correction. For snRNA-seq, cell type–specific differential expression was assessed using the Wilcoxon rank-sum test, with significance defined as adjusted *P* ≤ 0.05. For spatial transcriptomics, spatial domain– and microglia-targeted differential expression was assessed using MAST, with nontarget cell type fractions included as latent variables; pathway enrichment was reported at FDR ≤ 0.05. Normally distributed data (Shapiro-Wilk test) were analyzed using 2-tailed Welch’s unpaired *t* test or 1-way ANOVA (with Bonferroni’s correction). Non-normally distributed data were analyzed using the Mann-Whitney *U* test. Significance was defined as *P* or adjusted *P* ≤ 0.05. Box plots show the IQR, median (line), and whiskers extending to the most extreme observations within 1.5× IQR.

### Study approval.

Human studies were approved by the Institutional Review Board of the UPMC, Pittsburgh, Pennsylvania, USA (IRB #19050364 and #19070314), and all participants provided written informed consent.

### Data availability.

All materials and data from this study are available to researchers upon reasonable request and after issuance of relevant institutional data sharing agreements by contacting the corresponding authors. Human data with identifiers are restricted owing to patient confidentiality. The UKBB data can be accessed through the UKBB Research Analysis Portal (https://www.ukbiobank.ac.uk/enable-your-research). Raw and processed snRNA-seq and spatial transcriptomics data have been deposited in Gene Expression Omnibus under accession numbers GSE334773 and GSE334959, respectively. Analysis code is available with open access at the following GitHub repository: https://github.com/PittVMIOSB/Okawa_et_al (commit ID 3ae4b25). Any additional information required to reanalyze the data reported in this paper is available from the corresponding authors upon request. All raw data values are included in the Supporting Data Values file.

## Manuscript information

### Author contributions.

SO, YS, ANS, LDH, and SYC designed the study. YS, S Banerjee, YYT, JN, SG, SJ, MA, KK, SS, RS, DS, SU, and SSP conducted experiments. SO, ANS, TK, YAA, CP, OF, JMM, TDG, JTB, SBB, SP, S Banerjee, S Bonnet, and TH acquired data. SO, YS, S Banerjee, YYT, JN, TK, OF, NK, TH, and SN analyzed data. SO, YS, ANS, and SYC wrote the manuscript. SO, YS, ANS, TK, MA, JTB, SP, S Bonnet, SN, DS, and SYC edited the manuscript, and SYC supervised the work.

### AI disclosure.

Illustrae was used on August 20–25, 2026, as an aid to generate the Graphical abstract.

### Conflict of interest.

SYC served as a consultant to United Therapeutics and Merck; SYC held research grants from Bayer and United Therapeutics; and SYC is a director, officer, and shareholder of Synhale Therapeutics and Amlysion Therapeutics. SYC is an inventor on patents regarding metabolism and inflammation in PH. These include US 10,925,869 B2, “Compositions and methods for treating pulmonary vascular disease”; PCT/US2015/029286, “Coordinate control of pathogenic signaling by the MIR-130-301 family in pulmonary hypertension and fibroproliferative diseases”; and PCT/US2018/062013, “Compositions and methods for administering a YAP1/WWRT1 inhibiting composition and a GLS1 inhibiting composition.” SYC and LDH are inventors on patents and patent applications regarding the therapeutic targeting of NCOA7. These include US 11,773,391 B2, “Therapeutic and diagnostic target for SARS-CoV-2 and COVID-19,” and PCT application WO2023228154A1, “Methods and compositions for treatment of inflammation and inflammatory conditions.”

### Funding.

This study was supported by the following:

- NIH (R35GM164224), American Heart Association SFRN (26SFRNPVHD1609644), and NASA E9 Space Biology (80NSSC26K0720) grants to SO.
- WoodNext Foundation and American Heart Association SFRN (24SFRNPCN1280228) grants to SYC.
- American Heart Association CDA (24CDA1274310) grant to S Banerjee.
- Collaborative Research Center (1213 Project A10N) and (SA 1923/7-1) grants to RS.
- VA BLRD Research Career Scientist (IK6 BX005647-01) award to DS.
- VA Merit (I01BX006894-01) award to DS and SYC.
- NIH (R01HL161038) grant to SU.
- Collaborative Research Center (1213 Project A01, A05) and European Research Council Consolidator (866051) grants to SSP.

This work is the result of NIH funding, in whole or in part, and is subject to the NIH Public Access Policy. Through acceptance of this federal funding, the NIH has been given a right to make the work publicly available in PubMed Central.

## Supplementary Material

Supplemental data

Unedited blot and gel images

Supplemental table 1

Supplemental table 10

Supplemental table 11

Supplemental table 12

Supplemental table 13

Supplemental table 14

Supplemental table 15

Supplemental table 16

Supplemental table 17

Supplemental table 19

Supplemental table 20

Supplemental table 21

Supplemental table 22

Supplemental table 23

Supplemental table 24

Supplemental table 25

Supplemental table 26

Supplemental table 27

Supplemental table 28

Supplemental table 29

Supplemental table 5

Supplemental table 6

Supplemental table 9

Supporting data values

## Figures and Tables

**Figure 1:**
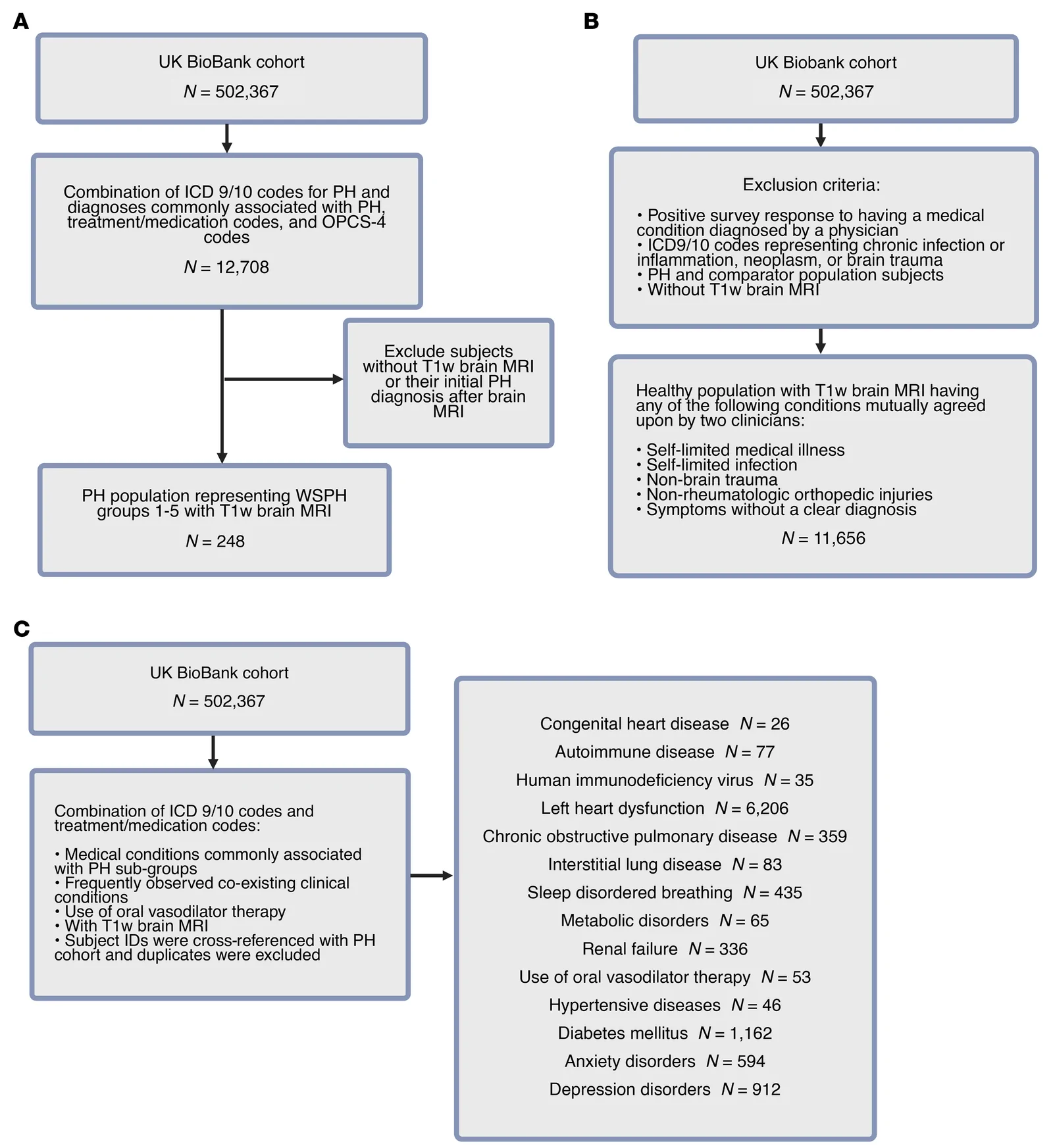
Flowchart to establish UK Biobank cohorts. (**A**) UK Biobank (UKBB) PH cohort. (**B**) UKBB control cohort. (**C**) UKBB comparator cohorts. List of diseases excluded from the control cohort is available in Supplemental Figure 1, and University of Pennsylvania Medical Center (UPMC) patient enrollment scheme is shown in Supplemental Figure 2. Sets of ICD-9/10 codes used to define each cohort are shown in Supplemental Table 1. T1-weighted (T1w) brain MRI.

**Figure 2:**
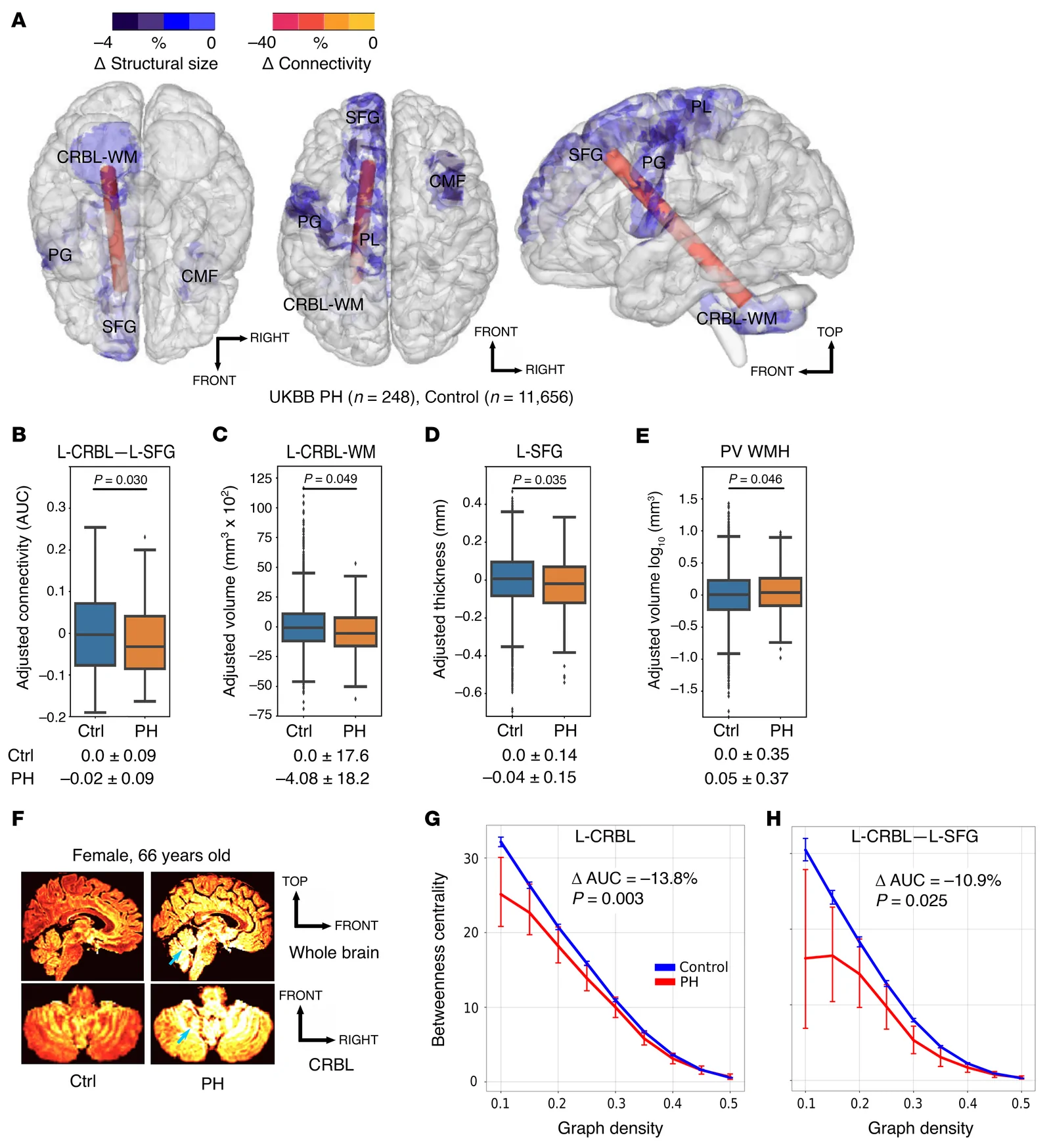
Brain structural and functional connectivity changes in UKBB PH. (**A**) UK Biobank (UKBB) T1-weighted (T1w) MRI and resting-state functional MRI showing PH-associated changes in brain volume, cortical thickness, and functional connectivity. Percentage differences reflect raw group means; significance was assessed by covariate-adjusted ordinary least squares (OLS) regression with Benjamini-Hochberg correction (Padj ≤ 0.05; Supplemental Table 4). Views: ventral, dorsal, and left lateral. CRBL-WM (cerebellar white matter), SFG (superior frontal gyrus), PG (precentral gyrus), PL (paracentral lobule), CMF (caudal middle frontal gyrus). (**B**–**D**) Covariate-adjusted L-CRBL–L-SFG connectivity, L-CRBL-WM volume, and L-SFG thickness in UKBB PH and controls, shown as OLS residuals with the group effect reintroduced. Significance was assessed by OLS regression with Benjamini-Hochberg correction: median (IQR), mean ± SEM underneath; *n* = 248 cases, *n* = 11,656 controls. (**E**) Covariate-adjusted perivascular white matter hyperintensity (PV WMH) from T2-weighted (T2w) FLAIR MRI: median (IQR), mean ± SEM underneath; *n* = 228 cases, *n* = 11,242 controls. (**F**) Representative T2w FLAIR images showing CRBL-WM WMH. (**G** and **H**) Global betweenness centrality across graph density thresholds for L-CRBL and L-CRBL–L-SFG; group differences were tested using AUC by OLS regression with permutation-derived *P* values (mean ± bootstrapped 95% CI; *n* = 193 cases, *n* = 10,242 controls).

**Figure 3:**
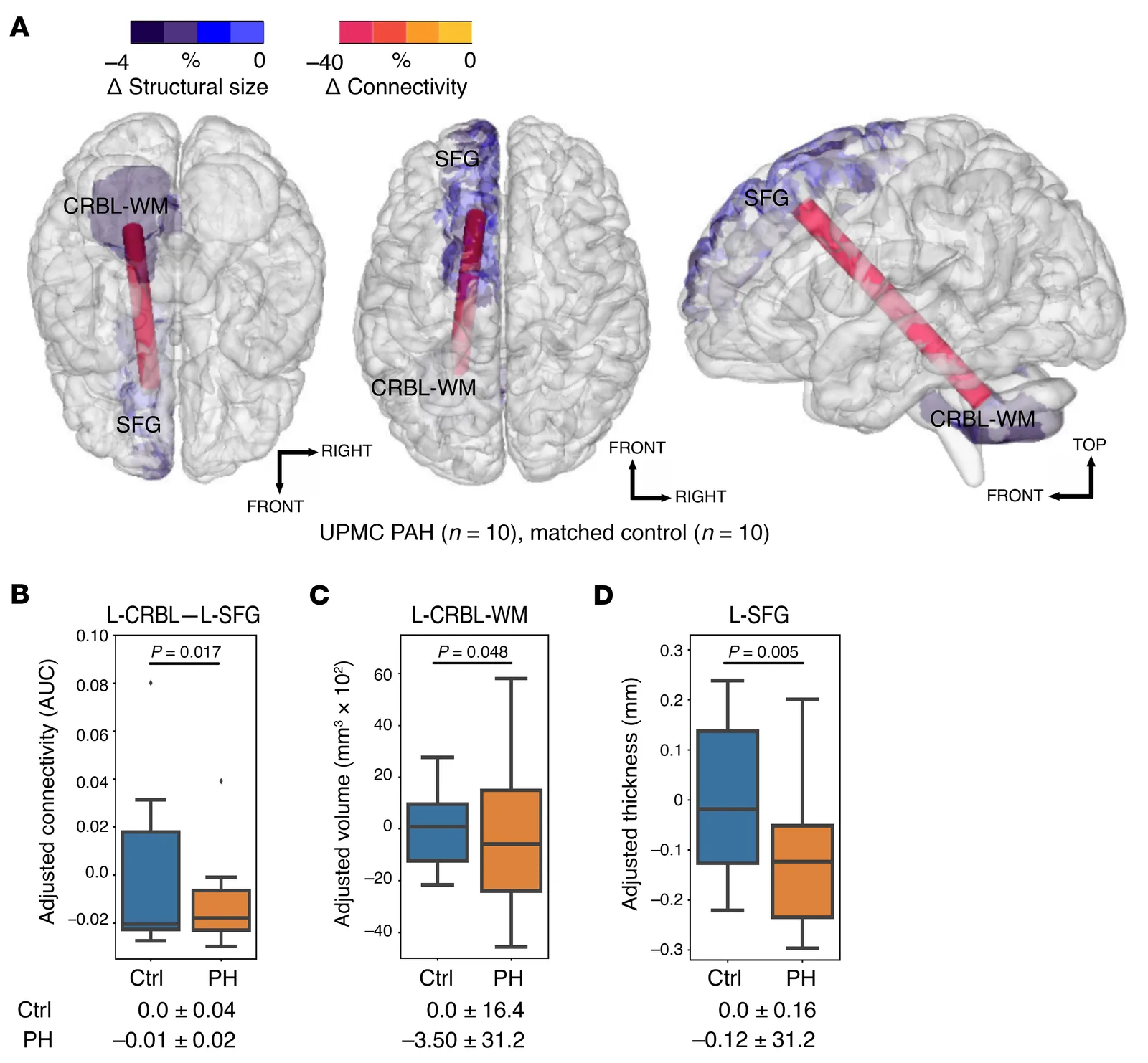
Brain structural and functional connectivity changes in UPMC PAH. (**A**) University of Pennsylvania Medical Center (UPMC) T1-weighted MRI (T1w-MRI) and resting-state functional MRI showing PAH-associated changes in brain volume, cortical thickness, and functional connectivity. Percentage differences reflect raw group means; significance was assessed by covariate-adjusted ordinary least squares (OLS) regression with permutation-derived *P* values (*P* ≤ 0.05; Supplemental Table 7). Views: ventral, dorsal, and left lateral. CRBL-WM, cerebellar white matter; SFG, superior frontal gyrus. (**B**–**D**) Covariate-adjusted L-CRBL–L-SFG connectivity, L-CRBL-WM volume, and L-SFG thickness: median (IQR), mean ± SEM underneath; *n* = 10 cases, *n* = 10 matched controls.

**Figure 4:**
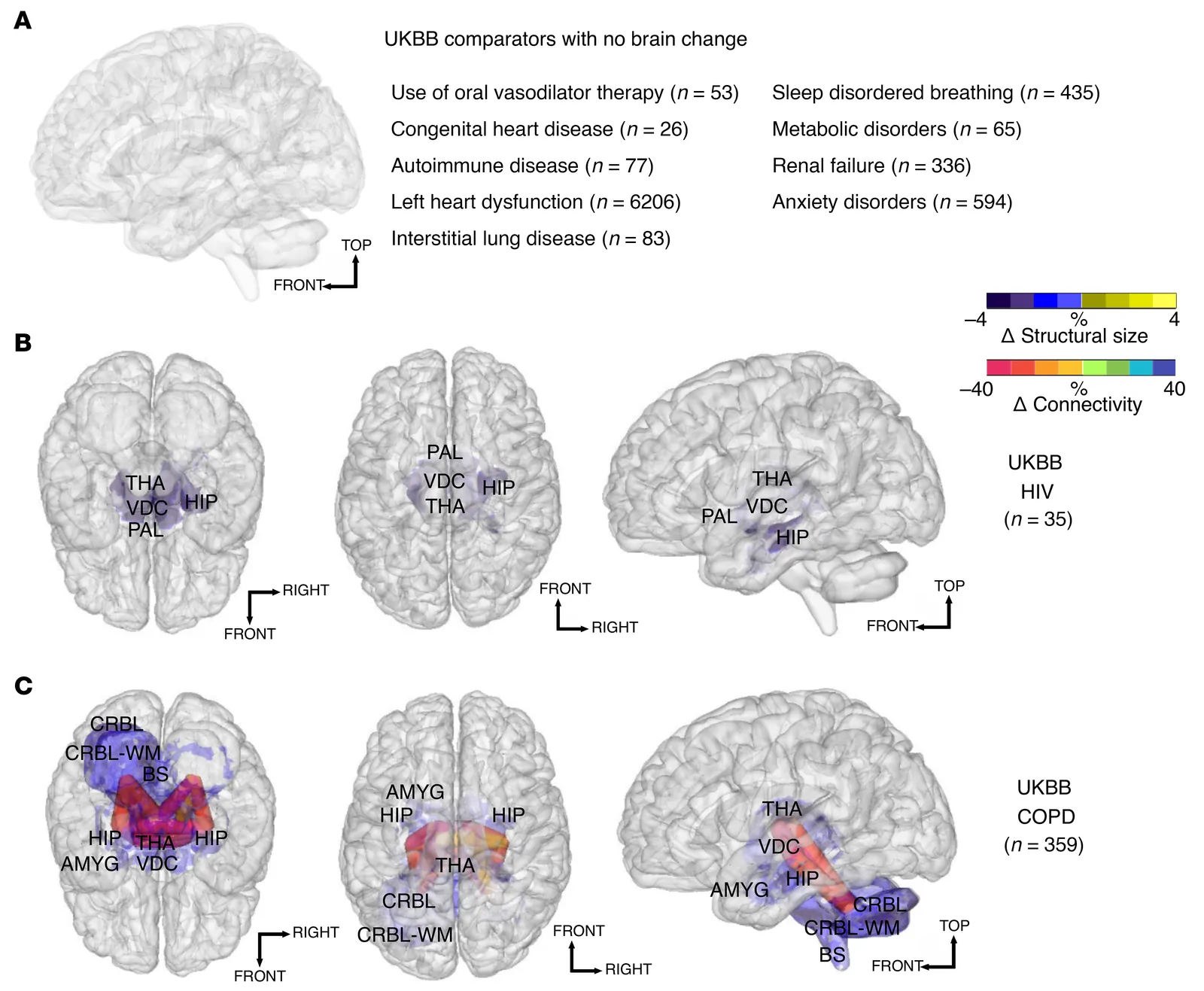
Brain structural and functional connectivity changes in UKBB comparator cohorts. (**A**) By T1-weighted (T1w) MRI and resting state functional MRI (rfMRI) in the UK Biobank (UKBB) population, when comparing patients with a range of chronic illnesses to a healthy control cohort (*n* = 11,656), no significant changes in volume and cortical thickness or functional connectivity were observed across any brain regions. In contrast, as compared with healthy controls, patients with specific conditions displayed significant changes: (**B**) HIV (*n* = 35). (**C**) COPD (*n* = 359). Left: ventral view, middle: dorsal view, right: left lateral view. VDC, ventral diencephalon; THA, thalamus; PAL, pallidum; HIP, hippocampus; CRBL, cerebellum; WM, white matter; BS, brain stem; AMYG, amygdala. Percentage differences reflect raw group means. Significance cutoff, Padj ≤ 0.05; NS, Padj > 0.05, by ordinary least squares (OLS) regression adjusted by Benjamini-Hochberg method (see also Supplemental Table 10).

**Figure 5:**
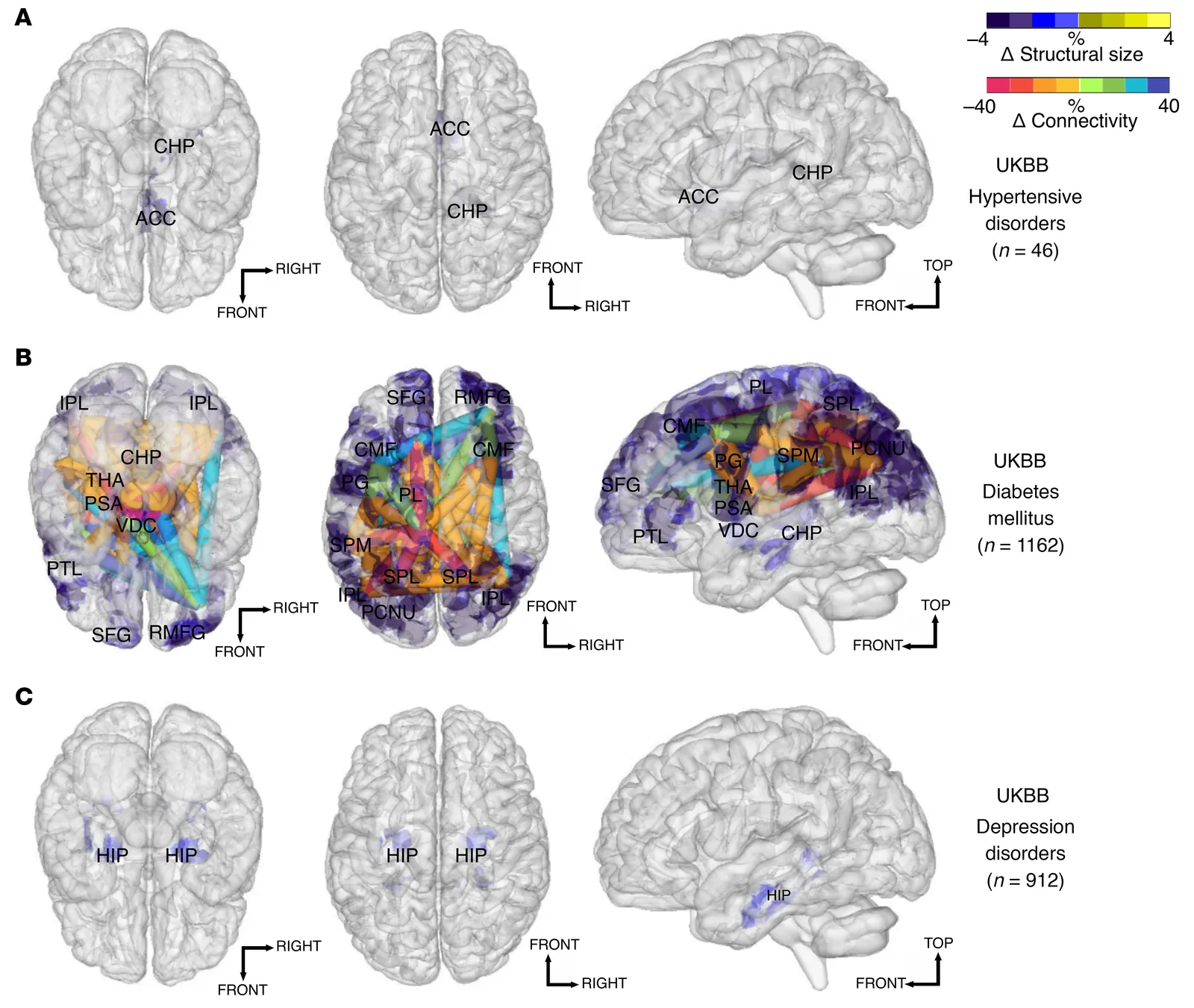
Brain structural and functional connectivity changes in UKBB potential confounding disease cohorts. (**A**) Hypertension (*n* = 46). (**B**) Diabetes mellitus (*n* = 1,162). (**C**) Depression (*n* = 912). Left: ventral view, middle: dorsal view, right: left lateral view. VDC, ventral diencephalon; THA, thalamus; PAL, pallidum; HIP, hippocampus; CHP, choroid plexus; ACC, accumbens; SPL, superior parietal lobe; IPL, inferior parietal lobule; SPM, supramarginal gyrus; SFG, superior frontal gyrus; RMFG, rostral middle frontal gyrus; PCNU, precuneus; PG, precentral gyrus; PSA, pars triangularis; PL, paracentral lobule; CMF, caudal middle frontal gyrus. Percentage differences reflect raw group means. Significance cutoff, Padj ≤ 0.05; NS, Padj > 0.05, by ordinary least squares (OLS) regression adjusted by Benjamini-Hochberg method (see also Supplemental Table 10).

**Figure 6:**
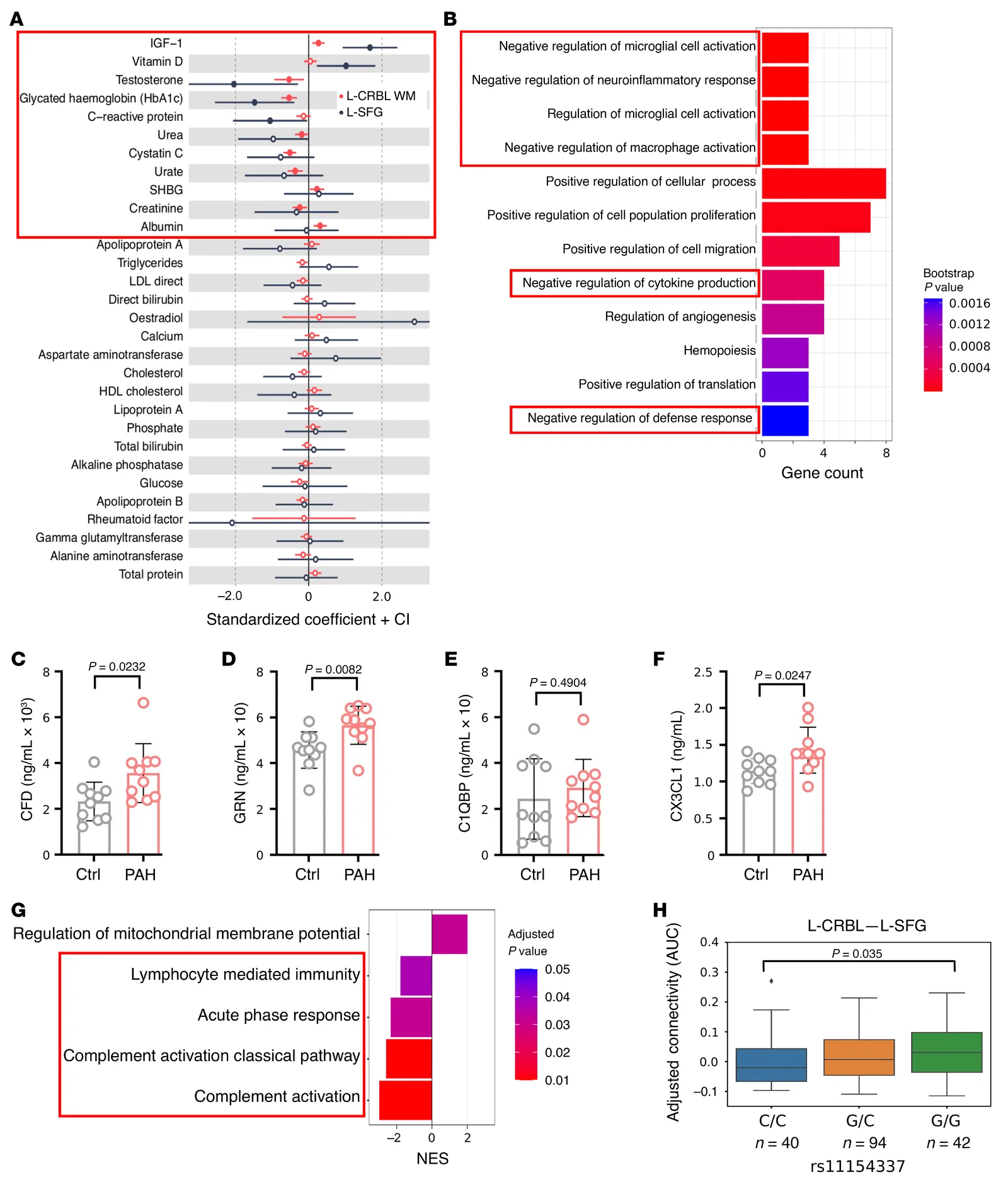
Systemic and cerebellar inflammatory signatures in PAH revealed by blood biomarkers. (**A**) Forest plot showing 30 blood biochemical markers and their statistical associations with L-CRBL-WM volume and L-SFG thickness among UK Biobank (UKBB) PH participants. *X* axis indicates standardized regression β-coefficient + CIs. Significant associations are indicated by filled circles. Significance cutoff, Padj ≤ 0.05; NS, Padj > 0.05, by ordinary least squares (OLS) regression using a generalized linear model (GLM) with bootstrapped *P* values and Benjamini-Hochberg adjustment (original *n* = 27–239; bootstrap resamples = 1,000; see also Supplemental Table 11). Top red square indicates markers significantly associated with at least 1 of the PH anatomic brain abnormalities. (**B**) Bar chart showing Gene Ontology Biological Processes (GOBP) categories enriched among the union of significantly negatively associated plasma proteins by Olink plasma proteomic analysis. Significance cutoff, Padj ≤ 0.05; NS, Padj > 0.05, by enrichR and FDR adjustment (see also Supplemental Figure 3 and Supplemental Tables 12 and 13). Red squares indicate immune response/inflammation categories. (**C**–**F**) Orthogonal ELISA validation of inflammatory plasma proteins in University of Pennsylvania Medical Center (UPMC) PAH individuals (*n* = 10) and age- and sex-matched healthy controls (*n* = 10). Data are mean ± SD. Significance cutoff, *P* ≤ 0.05; NS, *P* > 0.05. *P* values were determined by the Mann-Whitney *U* test (**C**) and 2-tailed Welch’s unpaired *t* tests (**D**–**F**). (**G**) Rank-based GOBP pathway fast gene set enrichment analysis of the rs11154337_G–Olink association in UK Biobank (UKBB) PH participants. Significance cutoff, Padj ≤ 0.05; NS, Padj > 0.05 by FDR adjustment. (**H**) Covariate-adjusted L-CRBL–L-SFG functional connectivity strength for UKBB PH cohort stratified by rs11154337 SNP allele genotype. Significance cutoff, *P* ≤ 0.05; NS, *P* > 0.05, by ordinary least squares (OLS) regress ion: median (IQR); *n* = 40 C/C, *n* = 94 G/C, *n* = 42 G/G alleles.

**Figure 7:**
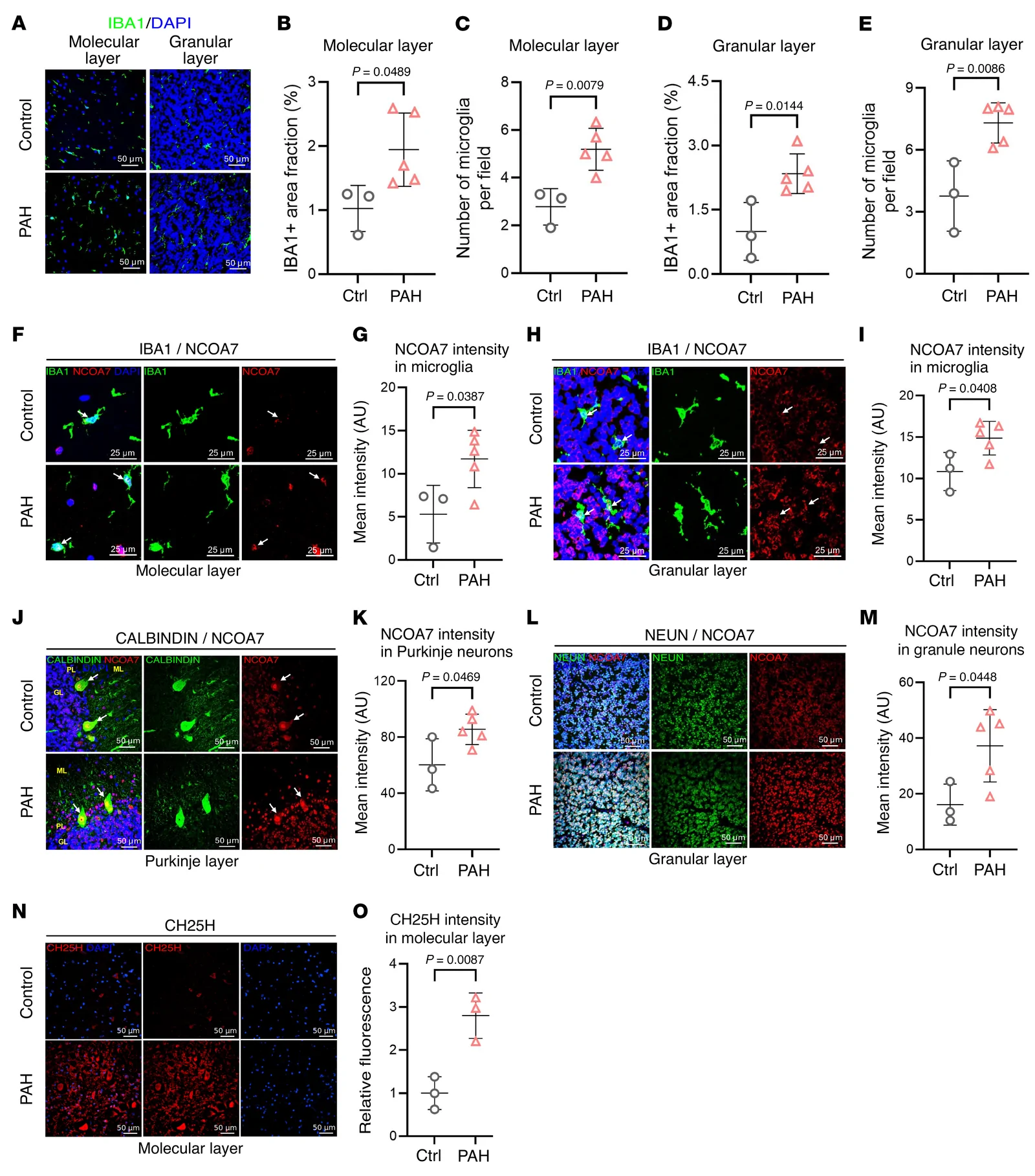
Immunofluorescence analysis of microglia and NCOA7 expression in postmortem human PAH cerebellum. (**A**–**E**) Ionized calcium-binding adapter molecule 1 (IBA1) immunofluorescence and quantification of IBA1^+^ microglia in postmortem human cerebellar tissue from control and PAH individuals. (**F**–**I**) Co-immunofluorescence of IBA1 and NCOA7, with quantification of NCOA7 within IBA1^+^ microglia in the molecular and granular layers. White arrows indicate NCOA7 expression in IBA1^+^ microglia. (**J** and **K**) Co-immunofluorescence and quantification of NCOA7 within CALBINDIN^+^ Purkinje neurons. White arrows indicate NCOA7 expression in CALBINDIN^+^ Purkinje neurons. (**L** and **M**) Co-immunofluorescence and quantification of NCOA7 within NEUN^+^ granule neurons. (**N** and **O**) CH25H immunofluorescence and quantification in the molecular layer. Data are mean ± SD. For **A**–**M**, *n* = 3 controls and *n* = 5 PAH cases; for **N** and **O**, *n* = 3 controls and *n* = 3 PAH cases. Significance cutoff, *P* ≤ 0.05; NS, *P* > 0.05, by 2-tailed Welch’s unpaired *t* test. Scale bars: 50 μm (**A**, **J**, and **L**), 25 μm (**F** and **H**). ML, molecular layer; PL, Purkinje layer; GL, granular layer.

**Figure 8:**
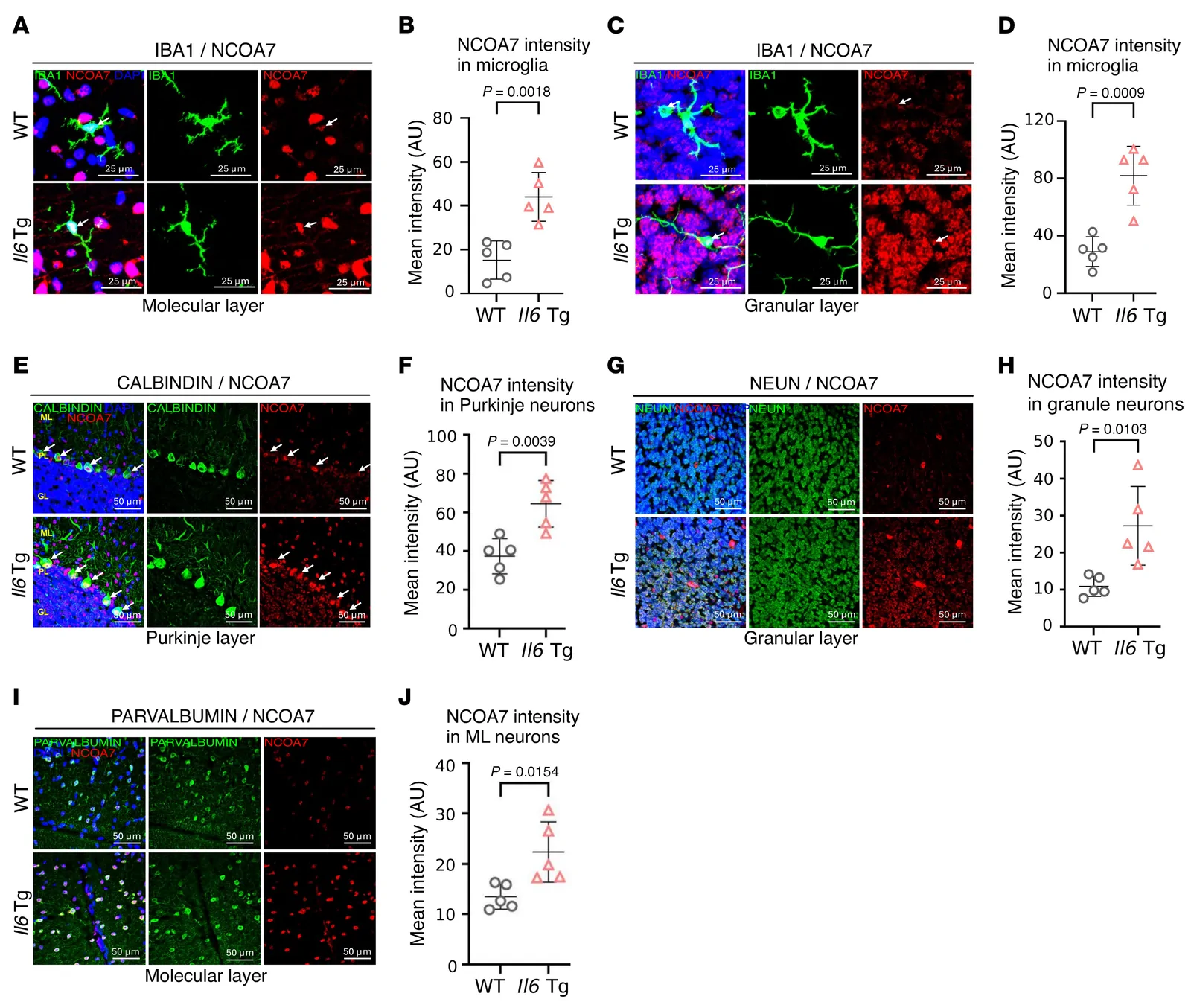
Immunofluorescence analysis of NCOA7 expression in inflammatory PH mouse models. (**A**–**D**) Co-immunofluorescence of ionized calcium-binding adapter molecule 1 (IBA1) and NCOA7 with quantification of NCOA7 within IBA1^+^ microglia in the molecular and granular layers. (**E** and **F**) Co-immunofluorescence and quantification of NCOA7 within CALBINDIN^+^ Purkinje neurons. (**G** and **H**) Co-immunofluorescence and quantification of NCOA7 within NEUN^+^ granule neurons. (**I** and **J**) Co-immunofluorescence and quantification of NCOA7 within PARVALBUMIN^+^ molecular layer neurons. Data are mean ± SD (*n* = 5 WT and *n* = 5 Il6-Tg), and *P* values were determined by 2-tailed Welch’s unpaired *t* test. Arrows indicate representative cells positive for both markers. Scale bars: 50 μm (**E**, **G**, and **I**) and 25 μm (**A** and **C**). ML, molecular layer; PL, Purkinje layer; GL, granular layer.

**Figure 9:**
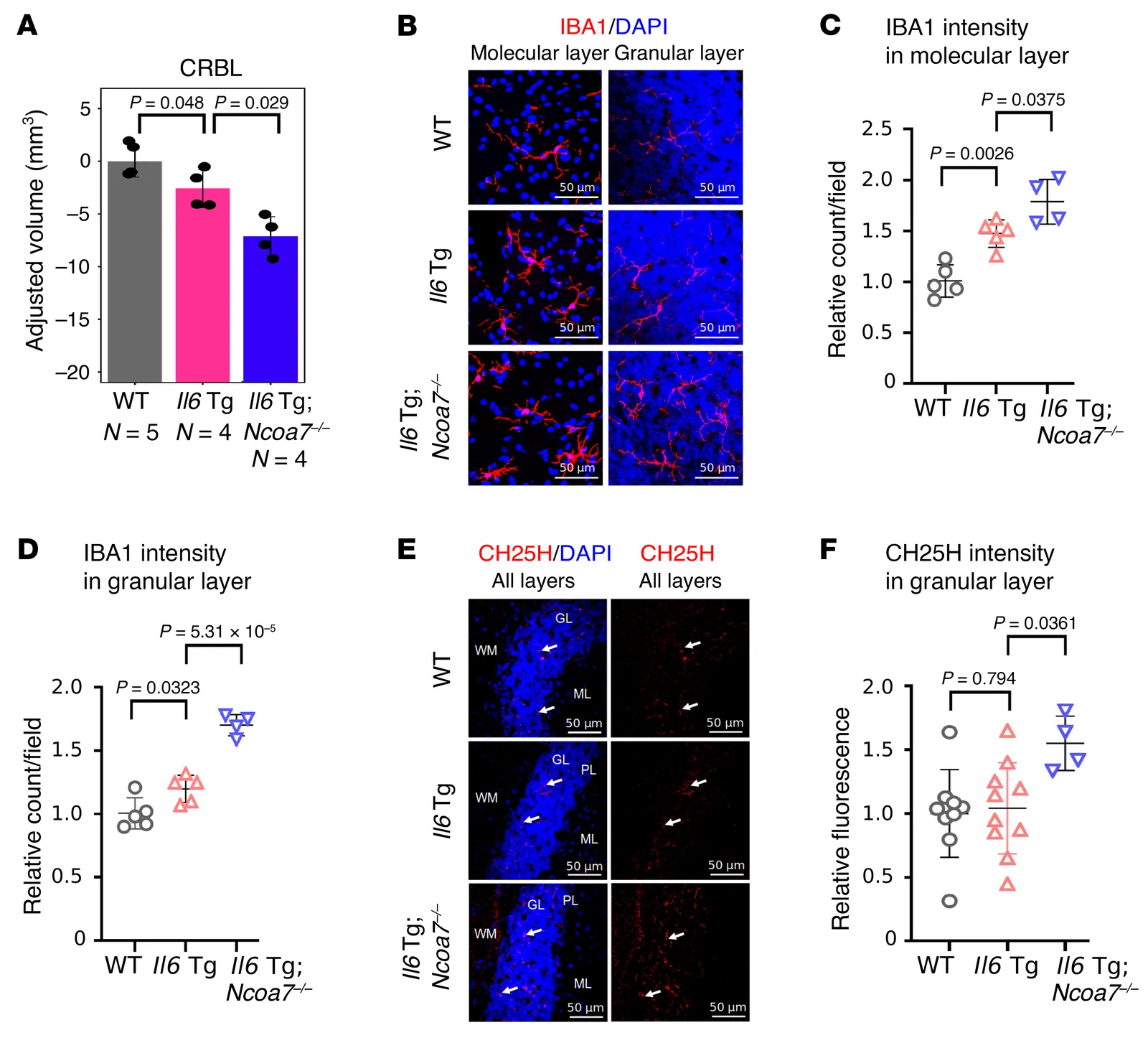
Evaluation of brain structure and histology in an inflammatory PH mouse model with Ncoa7 deficiency. (**A**) Covariate-adjusted cerebellar volume by ex vivo T2-weighted (T2w) MRI in WT, Il6-Tg, and Il6-Tg Ncoa7^–/–^ mice using ordinary least squares (OLS) regression with permutation-derived *P* value with Holm’s correction. (**B**) Representative cerebellar ionized calcium-binding adapter molecule 1 (IBA1) immunofluorescence in WT, Il6-Tg, and Il6-Tg Ncoa7^–/–^ mice. (**C** and **D**) Quantification of IBA1 expression in the molecular and granular layers, respectively. (**E**) Representative cerebellar CH25H immunofluorescence and (**F**) quantification of CH25H expression in the granular layer. Data are mean ± SEM (**A**) or mean ± SD (**C**, **D**, and **F**). For **A**, *n* = 5 WT, *n* = 4 Il6-Tg, and *n* = 4 Il6-Tg Ncoa7^–/–^; for **B**–**D**, *n* = 5 WT, *n* = 5 Il6-Tg, and *n* = 4 Il6-Tg Ncoa7^–/–^; and for **E** and **F**, *n* = 9 WT, *n* = 10 Il6-Tg, and *n* = 4 Il6-Tg Ncoa7^–/–^. *P* values for **C**, **D**, and **F** were determined by 1-way ANOVA with post hoc Bonferroni’s testing. Significance cutoff, Padj ≤ 0.05; NS, Padj > 0.05. Scale bars: 50 μm (**B** and **F**). ML, molecular layer; PL, Purkinje layer; GL, granular layer; WM, white matter.

**Figure 10:**
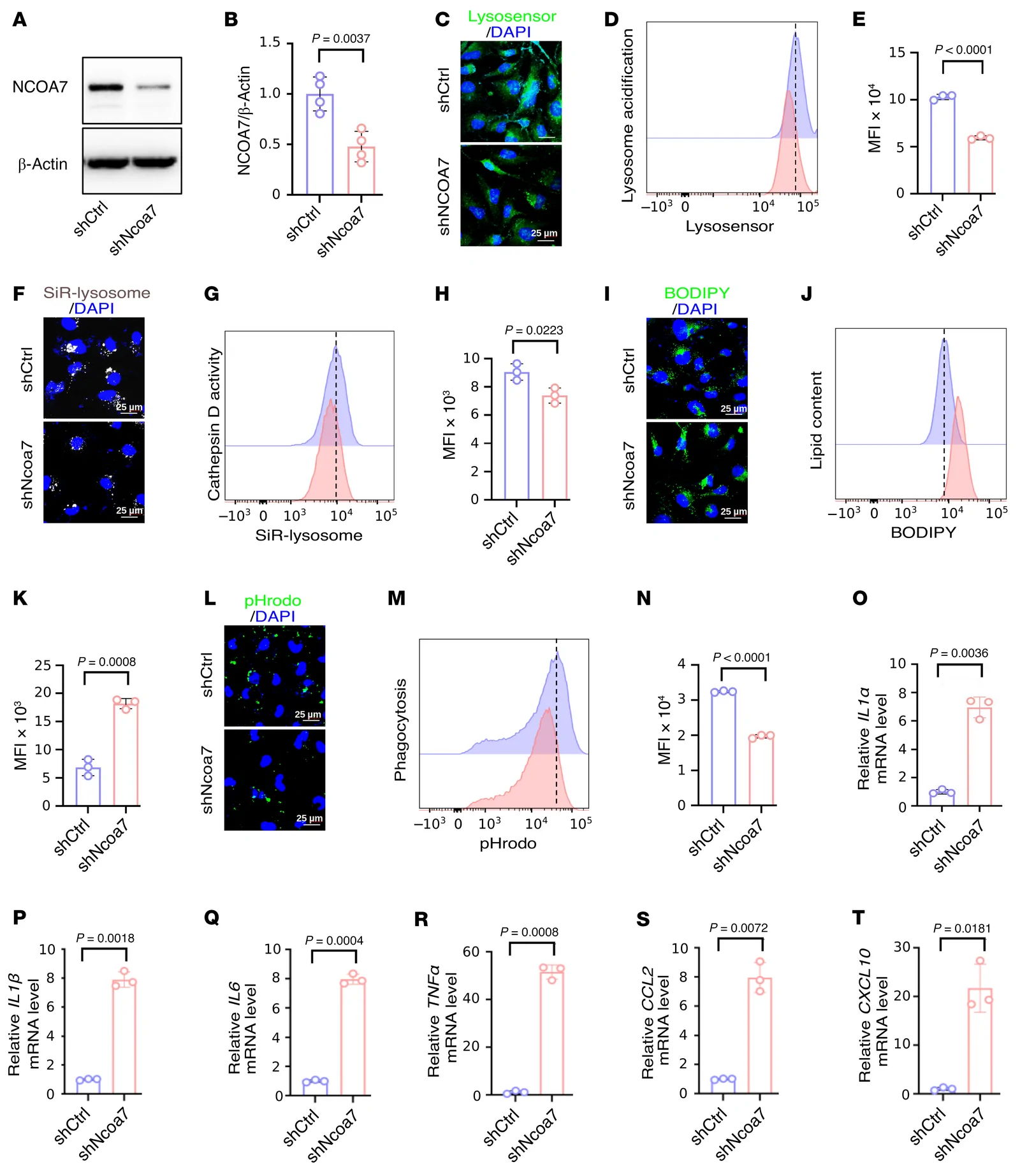
Functional consequences of NCOA7 loss in cultured human microglial cells. (**A**) Immunoblot of cultured HMC3 cells under shRNA control (shCtrl) and shRNA-mediated NCOA7 knockdown (shNCOA7) conditions, with (**B**) corresponding densitometric quantification (mean ± SD; *n* = 4 shCtrl, *n* = 4 shNCOA7). (**C**–**N**) Functional assays in HMC3 cells under shCtrl (*n* = 3) and shNCOA7 (*n* = 3) conditions. Representative images and quantification are shown for lysosomal acidification (LysoSensor; **C**–**E**), lysosomal protease activity/lysosome signal (SiR-lysosome; **F**–**H**), intracellular lipid accumulation (BODIPY; **I**–**K**), and phagocytic uptake of pHrodo-labeled brain synaptosomes (**L**–**N**). For each assay, panels show representative fluorescence images with DAPI counterstain (**C**, **F**, **I**, and **L**), flow cytometry histograms (**D**, **G**, **J**, and **M**), and corresponding quantitative analyses (mean ± SD) (**E**, **H**, **K**, and **N**). Significance cutoff, *P* ≤ 0.05; NS, *P* > 0.05, by 2-tailed Welch’s unpaired *t* test. (**O**–**T**) Relative mRNA expression levels of IL1α, IL1B, IL6, TNFα, CCL2, and CXCL10 in shCtrl- and shNCOA7-treated HMC3 cells (mean ± SD; *n* = 3 shCtrl, *n* = 3 shNCOA7). Significance cutoff, *P* ≤ 0.05; NS, *P* > 0.05 by 2-tailed Welch’s unpaired *t* test.

**Figure 11:**
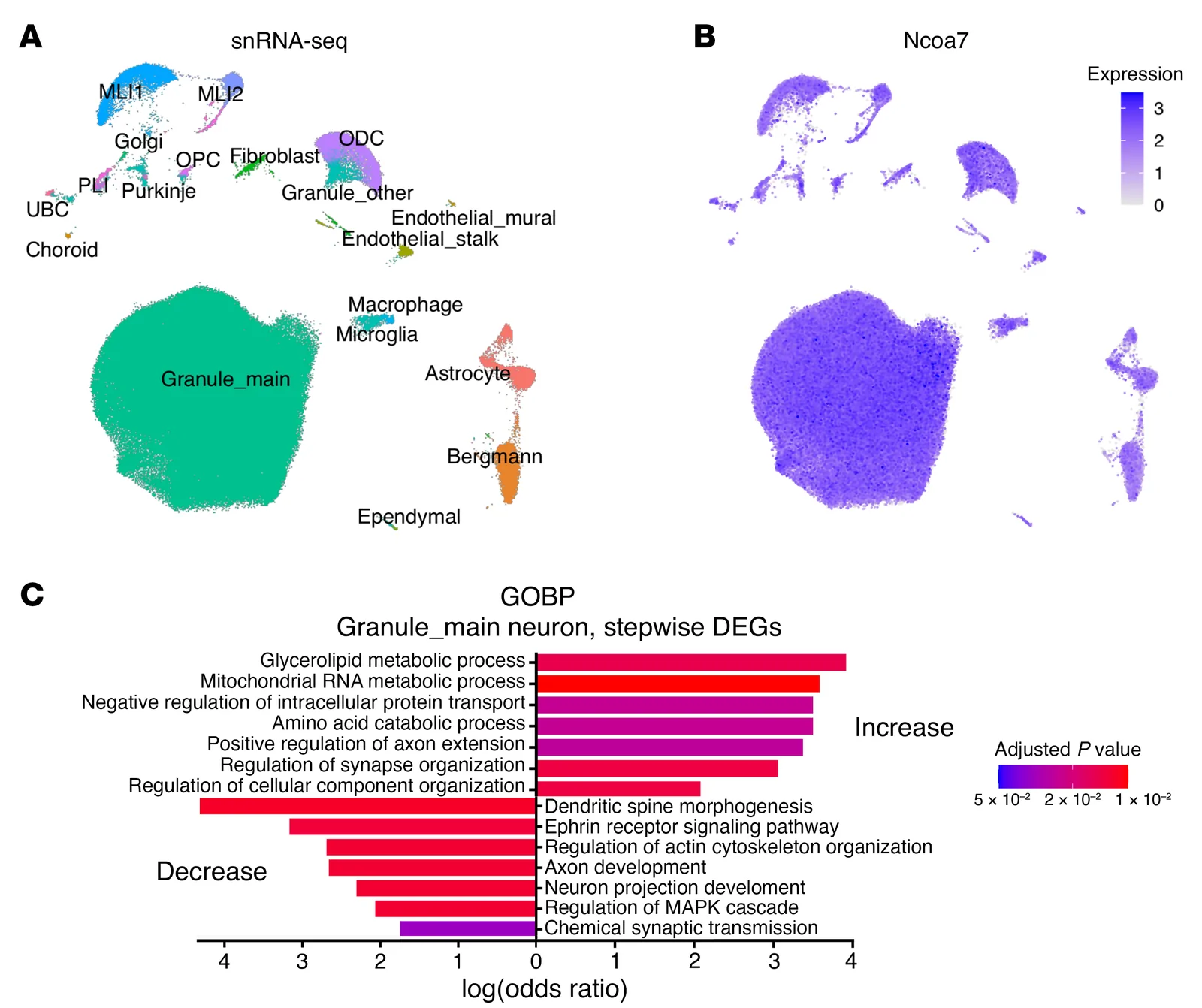
Single-nucleus transcriptomic analyses of PH- and Ncoa7-dependent cerebellar remodeling. (**A**) Uniform manifold approximation and projection (UMAP) visualization of cerebellar single-nucleus RNA-seq (snRNA-seq) nuclei from WT (*n* = 3), Il6-Tg (*n* = 4), and Il6-Tg Ncoa7^–/–^ (*n* = 4) mice annotated by reference-based label transfer. MLI, molecular layer interneuron; ODC, oligodendrocyte; OPC, oligodendrocyte progenitor cell; PLI, Purkinje layer interneuron; UBC, unipolar brush cell. (**B**) Feature plot showing Ncoa7 transcript detection across annotated snRNA-seq cell types. (**C**) Gene Ontology Biological Process (GOBP) enrichment of stepwise differentially expressed genes (DEGs) in Granule_main neurons across Il6-Tg vs. WT and Il6-Tg Ncoa7^–/–^ vs. Il6-Tg comparisons. Significance cutoff, Padj ≤ 0.05; NS, Padj > 0.05 by FDR adjustment.

**Figure 12:**
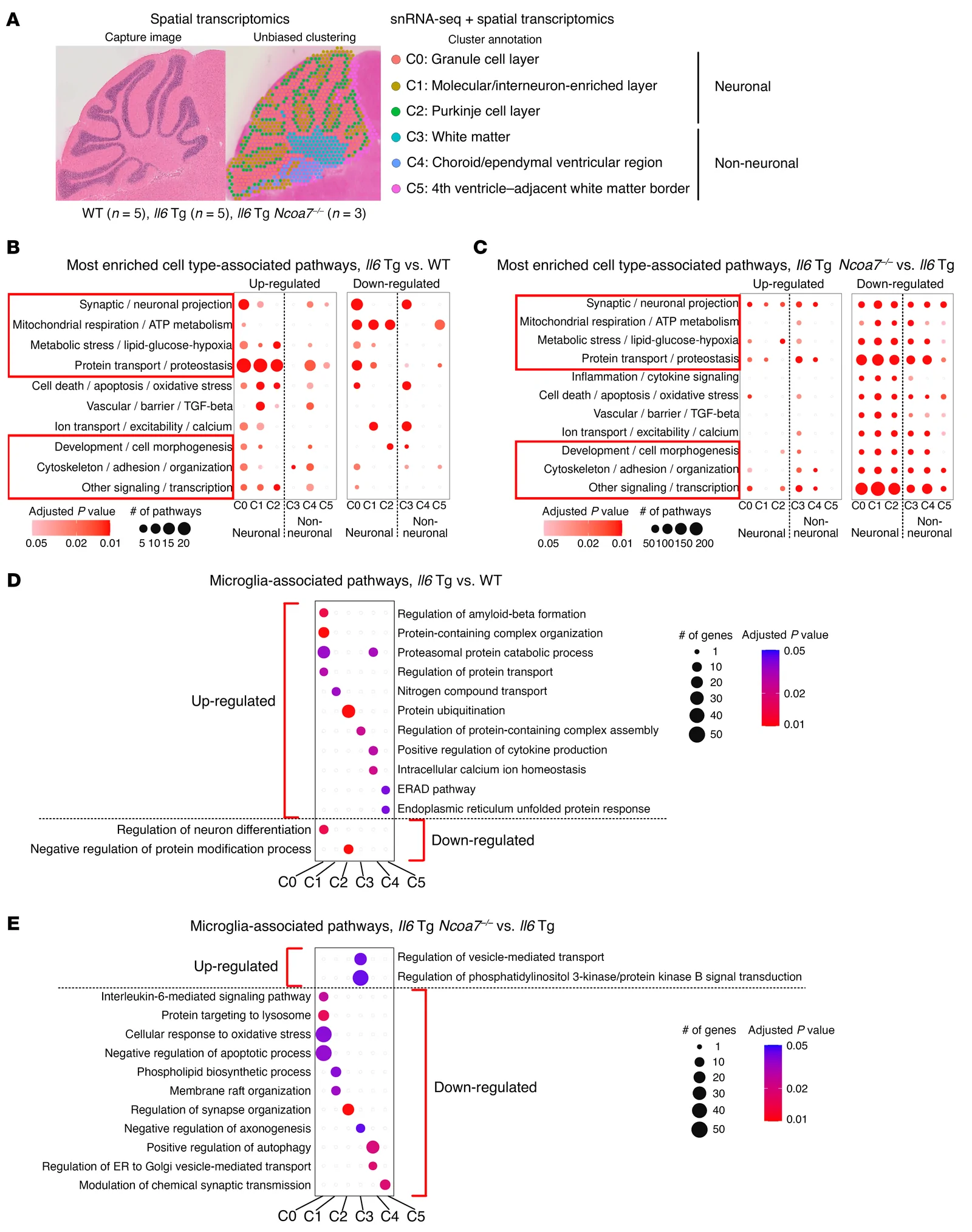
Spatial transcriptomic analyses of PH- and Ncoa7-dependent cerebellar remodeling. (**A**) Spatial transcriptomic captured image and unbiased clustering of mouse cerebellar sections. Subsequent cell type annotation of each cluster was carried out by integrating the snRNA-seq reference (see Supplemental Methods) (*n* = 5 WT, *n* = 5 Il6-Tg, and *n* = 3 Il6-Tg Ncoa7^–/–^). (**B** and **C**) Most enriched cell type–targeted spatial DEG-GSEA within each spatial cluster for Il6-Tg vs. WT and Il6-Tg Ncoa7^–/–^ vs. Il6-Tg, respectively, after adjustment for other local cell type signals; pathways were grouped into biological supercategories. Red squares indicate pathways also enriched in snRNA-seq Granule_main neurons. (**D** and **E**) Microglia-targeted spatial DEG-GSEA for Il6-Tg vs. WT and Il6-Tg Ncoa7^–/–^ vs. Il6-Tg, respectively, showing selected up- or downregulated pathways retained after removal of pathways identified in the corresponding most enriched cell type–targeted analysis. For all analyses, significance was defined as FDR-adjusted Padj ≤ 0.05; NS, Padj > 0.05. Cluster labels are abbreviated C0–C5.

## Version 1 11/02/2026

Electronic publication
