# Supplementary material for: Cerebellar and frontal lobe alterations in pulmonary hypertension implicate an inflammatory lung vascular/brain axis
Source: J Clin Invest. 2026 Nov 2;136(21):e195110. doi: 10.1172/JCI195110 (PMC13722401; doi:10.1172/JCI195110)
Supplement: Supplementary file 1 [file jci-136-195110-s031.pdf]

Supplemental Information

Cerebellar and frontal lobe alterations in pulmonary hypertension implicate an inflammatory lung vascular-brain axis

Satoshi Okawa, Yinan Shi, Somanshu Banerjee, Yi Yin Tai, Jamal Nabhanizadeh, Stefan Guenther, Anisha N. Shah, Siyi Jiang, Timothy Kim, Yassmin Al Aaraj, Caroline Pidro, Metin Aytekin, Omolara Fatukasi, Nishita Kalepalli, Joseph Mettenburg, Timothy Girard, James T. Becker, Kathiravan Kaliyappan, Shanshan Song, Sandra Breuils Bonnet, Steeve Provencher, Sébastien Bonnet, Theodore Huppert, Lloyd D. Harvey, Seyed Mehdi Nouraie, Rajkumar Savai, Dandan Sun, Soban Umar, Soni S. Pullamsetti, Stephen Y. Chan

Supplemental Figures

Medical condition diagnosed by a physician:

Heart attack
Angina
Stroke
High blood pressure
Deep venous thrombosis
Pulmonary embolus
Emphysema
Chronic bronchitis
Asthma
Hay Fever
Allergic rhinitis
Eczema
Diabetes
Cancer

ICD codes: neoplasm, chronic infections, systemic endocrine disorders, neurologic and psychiatric disorders, diseases of the cardiac or respiratory system, stroke, chronic oral infection, cirrhosis, inf ammatory rheumatologic conditions, maternal diseases complicating pregnancy, brain injury, organ transplant

Supplemental Figure 1. Definition of the UK Biobank (UKBB) control cohort. A list of diseases excluded from UKBB data fields and associated codes used in this process is detailed in Supplemental Table 1.

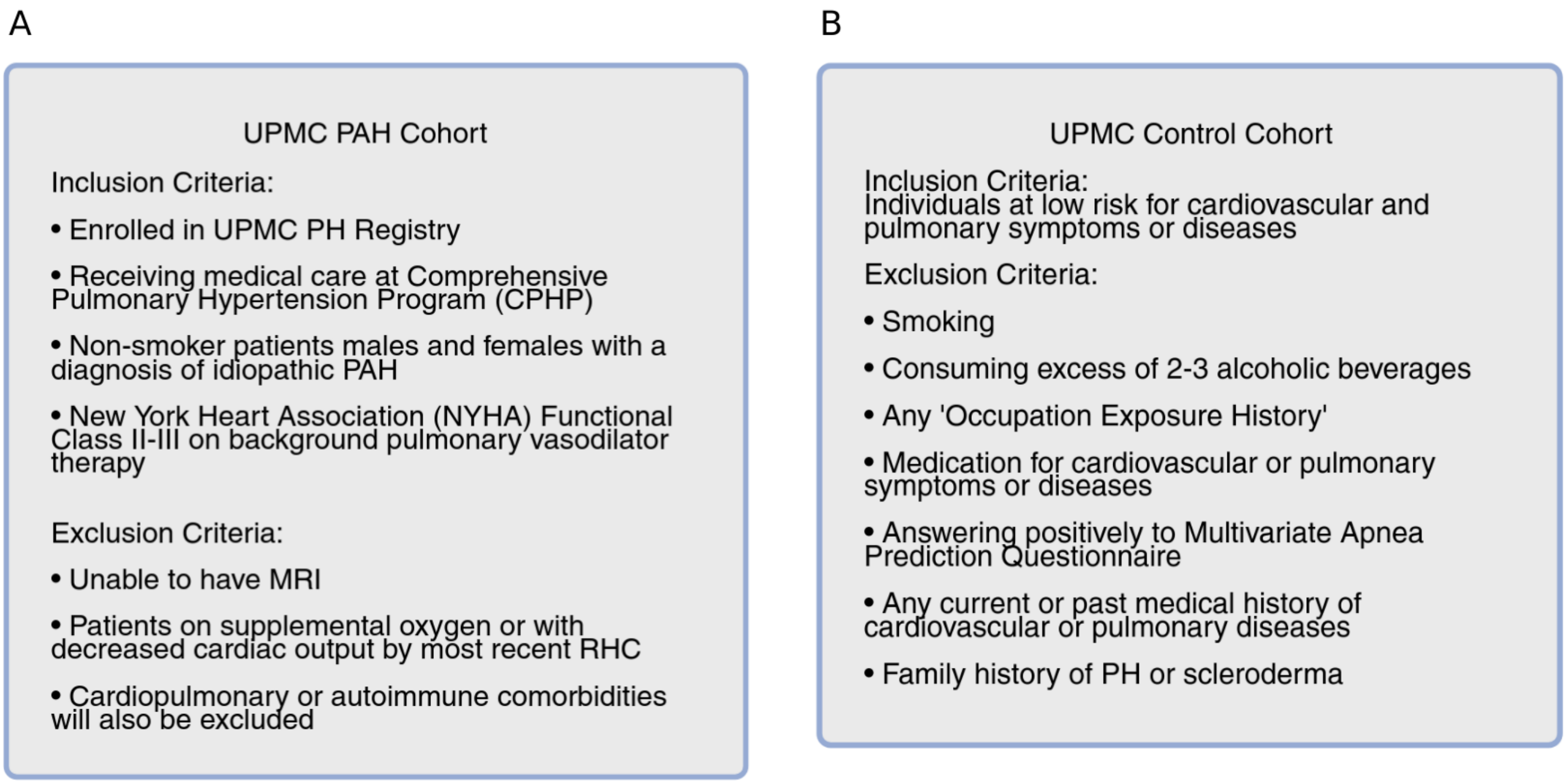

Supplemental Figure 2. Definition of the UPMC population. Inclusion and exclusion criteria for recruitment of (**A**) PAH patients and (**B**) control subjects at UPMC.

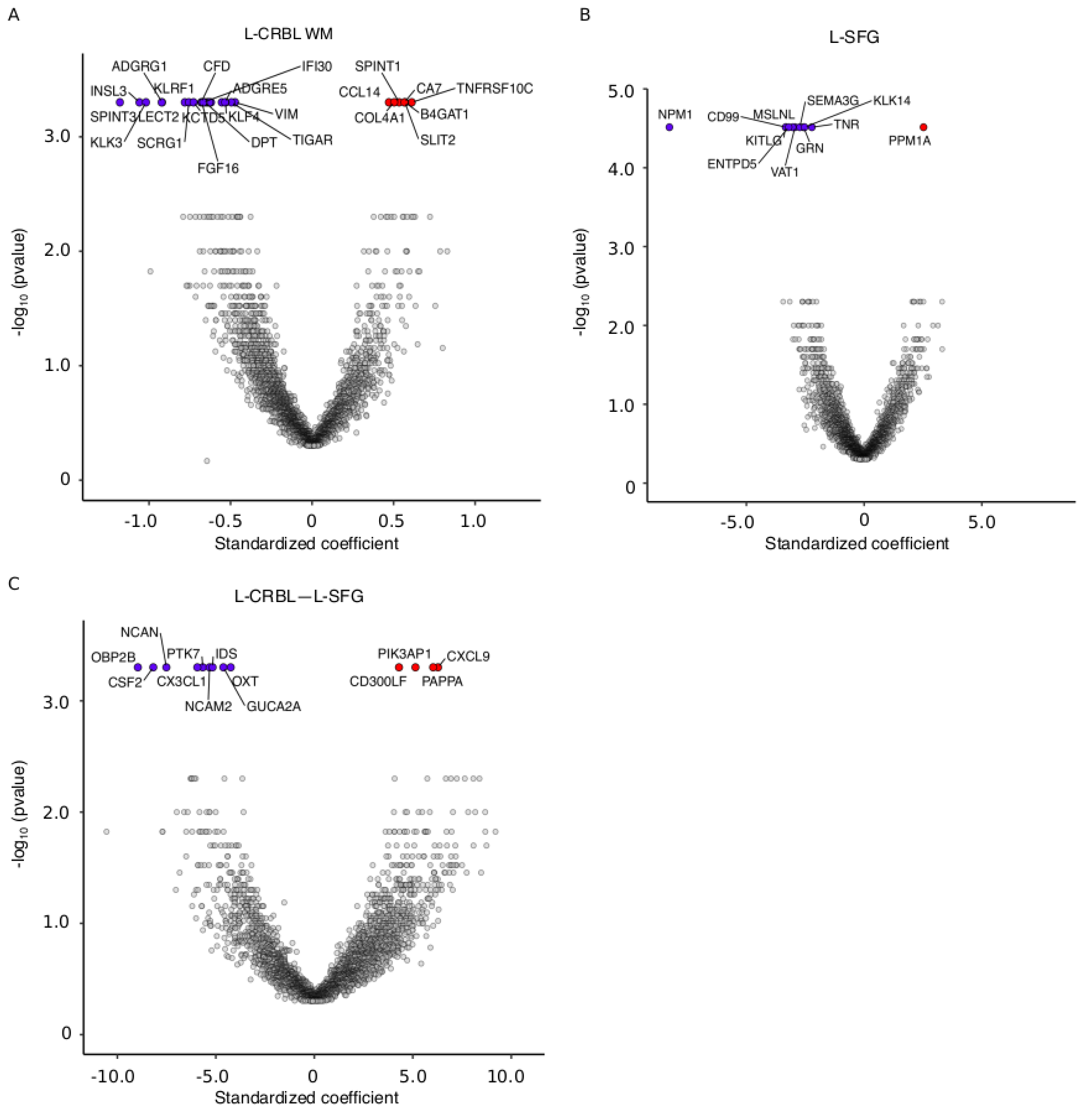

Supplemental Figure 3. Association of the UK Biobank plasma proteomic landscape with the L-CRBL—L-SFG (left-cerebellum—left-superior frontal gyrus) axis. A volcano plot shows associations between individual plasma protein expression levels and (**A**) L-CRBL-WM (white matter) volume, (**B**) L-SFG thickness, and (**C**) L-CRBL—L-SFG functional connectivity strength. Each point represents a single quantified plasma protein. X-axis indicates standardized regression coefficient

beta (β) and Y-axis represents raw p-values on a $-\log_{10}$ scale. Red denotes positive associations that passed the significance threshold. Statistical significance was determined based on the Benjamini-Hochberg–adjusted p-value ($P_{adj} \leq 0.05$); proteins with $P_{adj} > 0.05$ were considered not significant (ns), regardless of raw p-value. Regression was performed using a generalized linear model (GLM) with bootstrapped p-values and Benjamini-Hochberg adjustment (original n = 10-46; bootstrap resamples = 1,000; see also Supplemental Table 12).

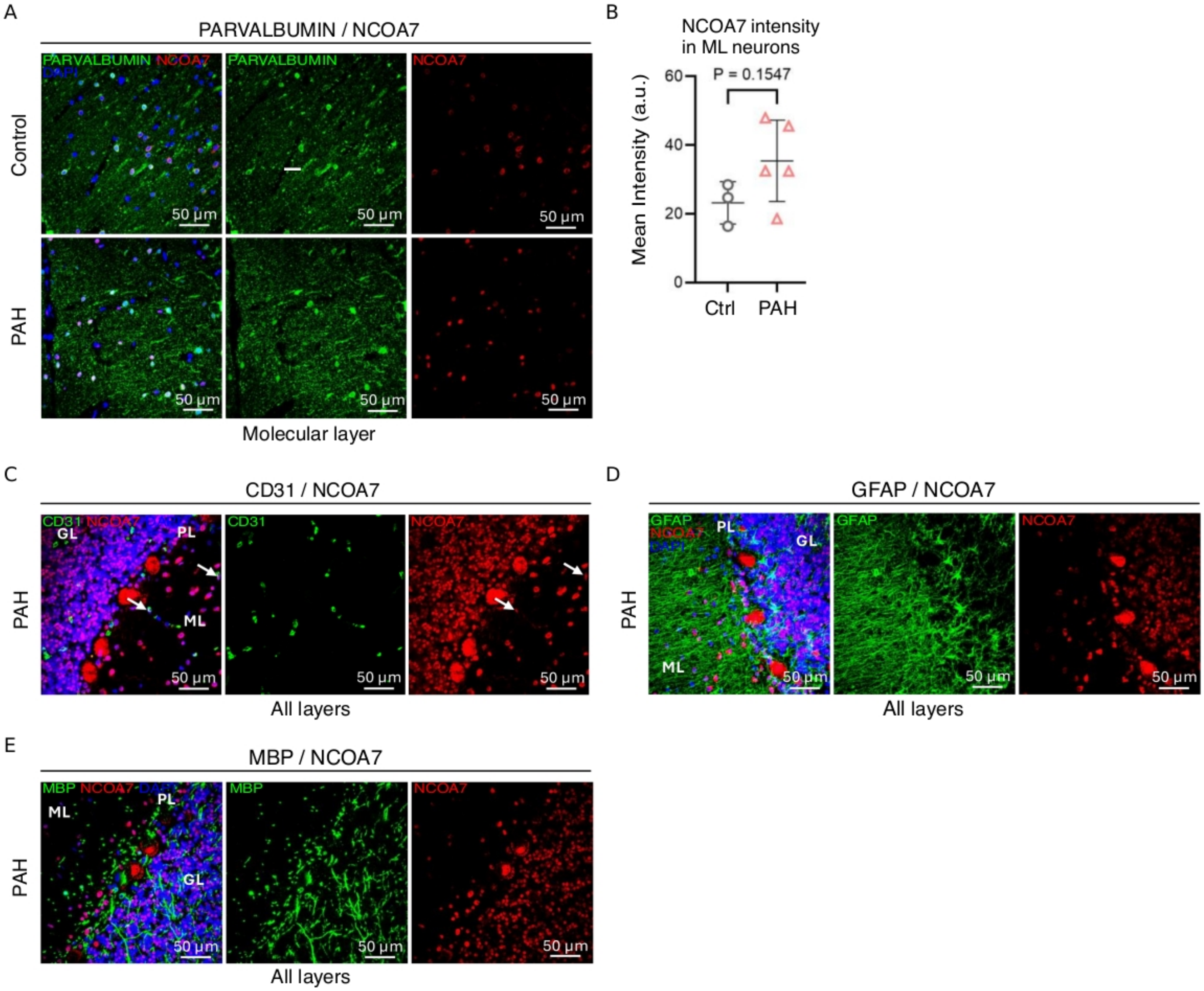

Supplemental Figure 4. Co-immunofluorescence of NCOA7 and cell type markers in postmortem human cerebellar tissue from PAH subjects. (**A**) Representative co-immunofluorescence images of NCOA7 and PARVALBUMIN in the cerebellar molecular layer. (**B**) Quantification of NCOA7 within PARVALBUMIN^{+} molecular-layer neurons (mean ± SD; n = 3 controls, n = 5 cases). Significance cutoff, $P \leq 0.05$; ns, $P > 0.05$, by two-tailed Welch's unpaired t-test. (**C-E**) Representative co-immunofluorescence images of NCOA7 and (**C**) CD31, (**D**) GFAP, and (**E**) MBP. Scale bars, 50 µm. ML, Molecular Layer; PL, Purkinje layer; GL, Granular Layer. White arrows indicate NCOA7 expression in CD31-positive endothelial cells.

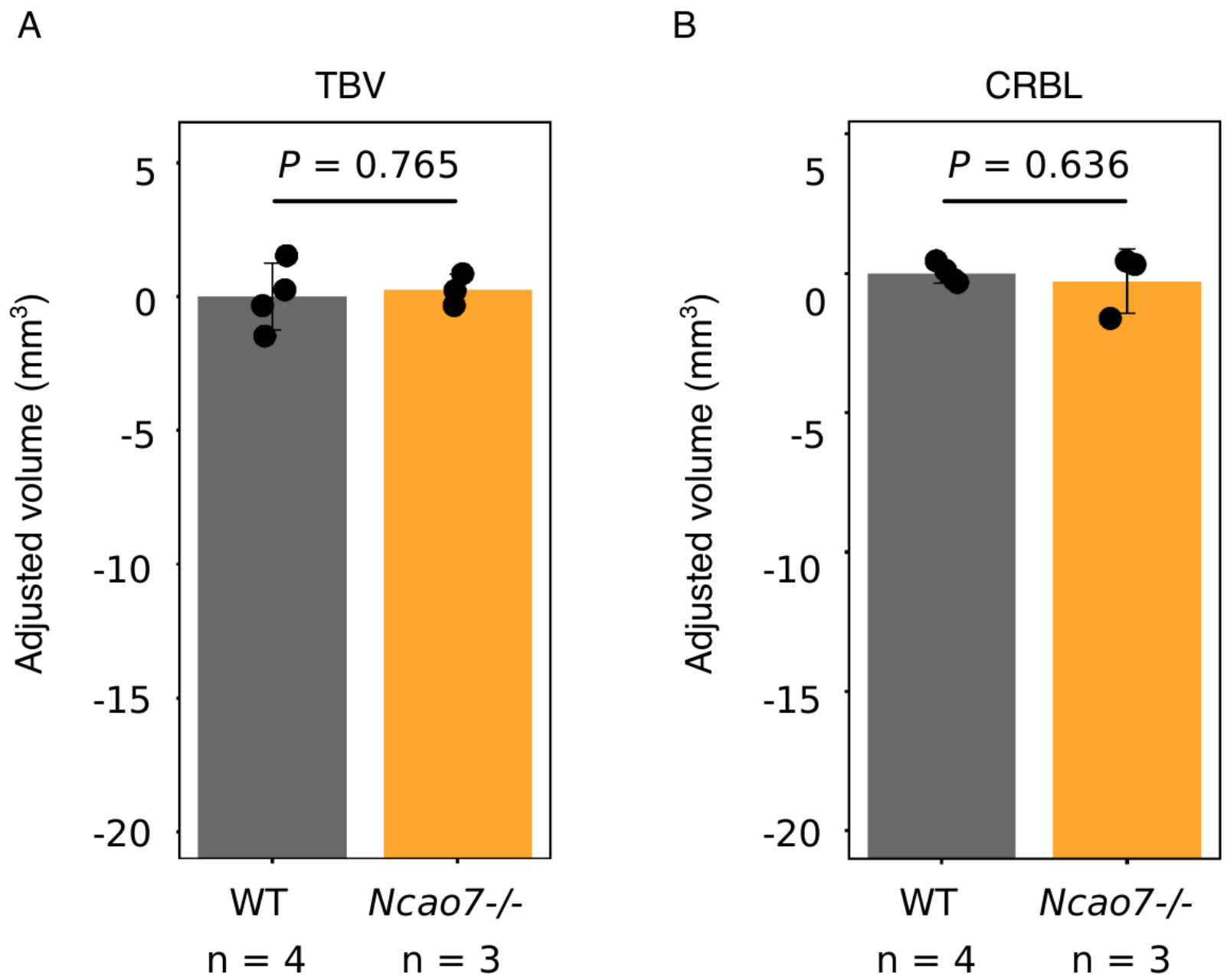

Supplemental Figure 5. *Ex vivo* MRI-based assessment of brain volume in WT and *Ncoa7*$^{-/-}$ mice. (**A**) Quantification of total brain volume and of (**B**) cerebellar volume (mean ± SEM; n = 4 WT, n = 3 Ncoa7-/-). Significance cutoff, P ≤ 0.05; ns, P > 0.05, by ordinary least squares regression with permutation-based p-value estimation.

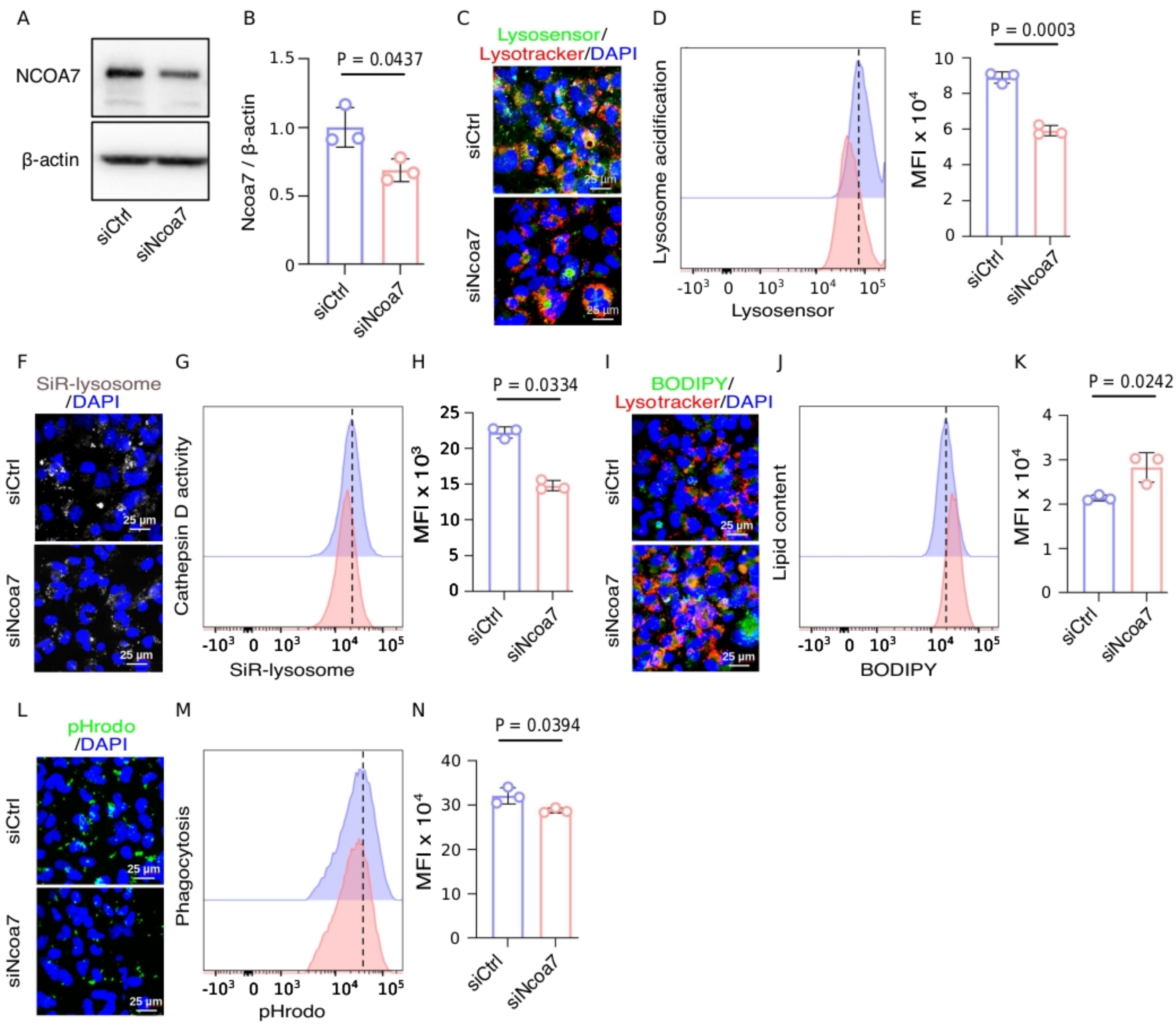

Supplemental Figure 6. HMC3 microglial cell functional assessment after siRNA NCOA7 knockdown. (**A**) Representative immunoblots of HMC3 cells under siRNA control (siCtrl), and siRNA-mediated NCOA7 knockdown (siNCOA7) conditions with (**B**) corresponding densitometric quantification (mean ± SD; n = 3 siCtrl, n = 3 siNCOA7). Significance cutoff, P ≤ 0.05; ns, P > 0.05, by two-tailed Welch's unpaired t-test. (**C–N**) Functional assays in HMC3 cells under siCtrl (n = 3) and siNCOA7 (n = 3) conditions. Representative images and quantification are shown for lysosomal acidification (LysoSensor; **C–E**), lysosomal protease activity/lysosome signal (SiR-lysosome; **F–H**), intracellular lipid accumulation (BODIPY; **I–K**), and phagocytic uptake of pHrodo-labeled brain

synaptosomes (**L–N**). For each assay, panels show representative fluorescence images with DAPI counterstain (**C, F, I, L**), flow cytometry histograms (**D, G, J, M**), and corresponding quantitative analyses (mean ± SD) (**E, H, K, N**). Significance cutoff, $P \leq 0.05$; ns, $P > 0.05$, by two-tailed Welch's unpaired t-test.

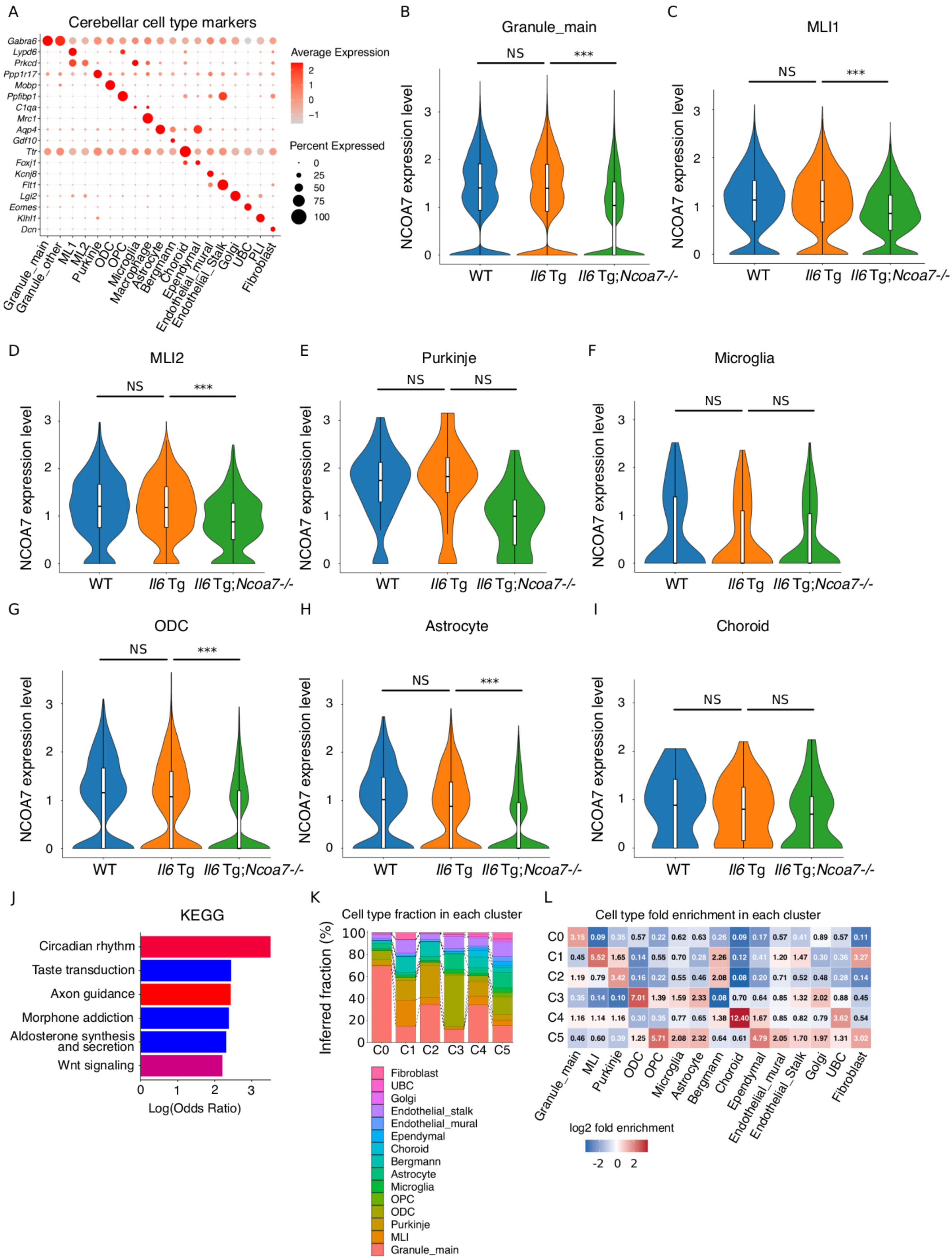
A
Cerebellar cell type markers
Gabra6
Lypd6
Prkcd
Ppp1r17
Mobp
Ppfibp1
C1qa
Mrc1
Aqp4
Gdf10
Ttr
Foxj1
Kcnj8
Flt1
Lgi2
Eomes
Klhl1
Dcn
Average Expression
Percent Expressed
Granule_main
Granule_other
ML1
ML2
Purkinje
ODC
OPC
Microglia
Macrophage
Astrocyte
Bergmann
Choroid
Ependymal
Endothelial_mural
Endothelial_Stalk
Golgi
UBC
PLI
Fibroblast
B
Granule_main
C
MLI1
D
MLI2
E
Purkinje
F
Microglia
G
ODC
H
Astrocyte
I
Choroid
NS
***
NCOA7 expression level
WT
Il6 Tg
Il6 Tg;Ncoa7-/-
J
KEGG
Circadian rhythm
Taste transduction
Axon guidance
Morphone addiction
Aldosterone synthesis and secretion
Wnt signaling
Log(Odds Ratio)
K
Cell type fraction in each cluster
Inferred fraction (%)
C0 C1 C2 C3 C4 C5
Fibroblast
UBC
Golgi
Endothelial_stalk
Endothelial_mural
Ependymal
Choroid
Bergmann
Astrocyte
Microglia
OPC
ODC
Purkinje
MLI
Granule_main
L
Cell type fold enrichment in each cluster
C0 3.15 0.09 0.35 0.57 0.22 0.62 0.63 0.26 0.09 0.17 0.57 0.41 0.89 0.57 0.11
C1 0.45 5.52 1.65 0.14 0.55 0.70 0.28 2.26 0.12 0.41 1.20 1.47 0.30 0.36 3.27
C2 1.19 0.79 3.42 0.16 0.22 0.55 0.46 2.08 0.08 0.20 0.71 0.52 0.48 0.28 0.14
C3 0.35 0.14 0.10 7.01 1.39 1.59 2.33 0.08 0.70 0.64 0.85 1.32 2.02 0.88 0.45
C4 1.16 1.14 1.16 0.30 0.35 0.77 0.65 1.38 12.40 1.67 0.85 0.82 0.79 3.62 0.54
C5 0.46 0.60 0.39 1.25 5.71 2.08 2.32 0.64 0.61 4.79 2.05 1.70 1.97 1.31 3.02
Granule_main
MLI
Purkinje
ODC
OPC
Microglia
Astrocyte
Bergmann
Choroid
Ependymal
Endothelial_mural
Endothelial_Stalk
Golgi
UBC
Fibroblast
log2 fold enrichment

Asdfshh

Supplemental Figure 7. snRNA-seq and spatial transcriptomic analyses of mouse cerebellum. (**A**) Dot plot showing canonical marker-gene enrichment supporting reference-based annotation of cerebellar snRNA-seq cell types. (**B–I**) Violin plots showing Ncoa7 transcript levels across selected snRNA-seq cell types and conditions (median (IQR); n = 3 WT, n = 4 *Il6* Tg, n = 4 *Il6* Tg;*Ncoa7-/-*). NS: not significant, ***: adjusted P-value ≤ 0.005. (**J**) Bar plot showing KEGG pathway enrichment among stepwise decreased differentially expressed genes (DEGs) in Granule_main neurons. Significance cutoff, *Padj* ≤ 0.05; ns, *Padj* > 0.05 by FDR-adjustment. (**K**) Stacked bar plot showing Robust Cell Type Decomposition (RCTD)-inferred cellular composition of spatial transcriptomic clusters. (**L**) Heatmap showing fold enrichment of snRNA-seq-derived cell-type deconvolution across spatial clusters, calculated as the mean cell-type fraction within each cluster divided by the mean fraction in all remaining clusters. Cluster labels are abbreviated as C0-C5.

Supplemental Tables and Supplemental Table Legends

Supplemental Table 1. Diagnosis codes used to define PH, control, and other comparator cohorts in UK Biobank. ICD-9/ICD-10 (International Classification of Diseases 9th and 10th revisions) and OPCS (Office of Population Censuses and Surveys) codes are shown. Disease abbreviations are as follows: Use of oral vasodilator treatment (UOVT), congenital heart disease (CHD), autoimmune diseases (AI), human immunodeficiency virus (HIV), left heart dysfunction (LHD), chronic obstructive pulmonary disease (COPD), interstitial lung disease (ILD), and sleep disordered breathing (SDB), metabolic disease (MD), renal failure (RF), hypertensive disorders (HD), diabetes mellitus (DM) and depression disorders (DD). Data provided in Excel format. In the Inclusion/Exclusion column, 1 indicates inclusion and -1 indicates exclusion.

Supplemental Table 2. UK Biobank (UKBB) and UPMC cohort characteristics. (A) UKBB PH and control cohorts. (**B**) UPMC PAH and matched control cohorts. For UKBB analyses comparing PH and control cohorts, the following covariates were used: age, sex, education, smoking history (ever smoked), BMI, circulatory disease – essential (primary) hypertension, circulatory disease – extravascular cranial, and endocrine diseases – diabetes (see Supplemental methods). For UPMC analyses, covariates included age, sex, and education. LV (left ventricle), PWA (pulse wave analysis). Values represent the median [25th–75th percentile], unless otherwise specified.

A

**UKBB**	**Case**	**Control**	**P**
	248	11656	
Age (years)	68 [61 73]	63 [57 69]	2.51E-11
Sex [female], n (%)	108 [44%]	6334 [54%]	8.23E-04
Education [college degree], n (%)	101 [41%]	5423 [47%]	6.76E-02
Ethnic group [white British], n (%)	248 [100%]	11656 [100%]	1.00E+00
Ever smoked, n (%)	123 [50%]	5891 [51%]	7.69E-01
BMI	27.0 [25.18 30.62]	25.5 [23.1 27.6]	4.89E-17
Handedness [right], n (%)	216 [87%]	10373 [89%]	4.31E-01
LV cardiac output	6.31 [5.62 6.65]	5.21 [4.45 6.01]	2.15E-04
Cardiac output	4.6 [4.0 5.3]	4.6 [3.9 5.3]	9.90E-03
Ventricular rate	61.5 [56.0 68.75]	60.0 [54.0 66.0]	1.47E-02
Central systolic blood pressure during PWA	132.0 [124.0 143.25]	132.0 [121.0 144.0]	1.07E-01
MRI time from initial diagnosis (years)	7.2 [3.0 13.0]		
Circulatory disease – Essential (primary) hypertension, n (%)	65 [26.4%]	0 [0.0%]	1.45E-18
Circulatory disease – Extravascular cranial, n (%)	4 [1.6%]	0 [0.0%]	4.53E-02
Endocrine diseases – Diabetes, n (%)	19 [7.5%]	0 [0.0%]	9.39E-06

B

**UPMC**	**Case**	**Control**	**P**
	10	10	
Age (years)	40 [38 47]	39 [36 48]	0.791
Sex [female] (%)	8 [80%]	8 [80%]	1
Education [college degree] (years)	15 [12 16]	16 [14 17]	0.336
Race [white]	10 [100%]	10 [100%]	1
Mean pulmonary artery pressure	44.5 [37.5 50.5]	NA	NA
Pulse oximetry (%)	97 [95 98]	NA	NA
Pulmonary arterial systolic pressure	75 [56.8 79.5]	NA	NA
Pulmonary vascular resistance	8.6 [7.2 10.8]	NA	NA
MRI time from initial diagnosis (years)	3.7 [2.4 7.2]		

Supplemental Table 3. Demographic and clinical characteristics of UPMC PAH patients. mPAP (mean pulmonary artery pressure), PO (pulse oximetry), PASP (pulmonary arterial systolic pressure), PVR (pulmonary vascular resistance), WU (Wood Units).

Ethnicity	Days from date of diagnosis to MRI	Age (y)	Sex (M/F)	mPAP (mm Hg)	PO (%)	PASP (mm Hg)	PVR (WU)
White	343	26	F	34	98	30	6.50
White	1598	39	F	45	98	55	7.48
White	4592	43	F	34	96	49	2.17
White	1356	58	M	53	98	92	10.57
White	1348	39	M	44	99	78	9.70
White	3589	46	F	49	95	77	11.43
White	2981	51	F	55	94	90	5.90
White	748	48	F	36	92	62	7.41
White	1353	35	F	42	95	73	4.94
White	161	38	F	51	99	80	19.8

Supplemental Table 4. Significantly altered brain structural regions and their functional connectivity in UK Biobank PH cohort (PH: $N = 248$; Control: $N = 11{,}656$). Structural and functional MRI metrics that showed significant differences between PH and control subjects based on ordinary least squares (OLS) regression are listed. Effect sizes (Beta), confidence intervals (CI), and adjusted p-value (Padj) are reported from covariate-adjusted models with Benjamini–Hochberg correction for multiple testing. Significance cutoff, $Padj \leq 0.05$; ns, $Padj > 0.05$. Percent differences represent raw group-level mean differences (unadjusted), reported to aid interpretability of group-level magnitude. AUC (area under curve), DF (data-field). See Figure 2, A–D for visualization of adjusted outcomes.

IDP (image-derived phenotype) [unit]	Measure	Beta	Padj	CI 025	CI 975	Case – Control (%)	UKBB DF
L-SFG (left superior frontal gyrus) [mm]	thickness	-0.0366	0.035	-0.057	-0.016	-2.08	27199
L-PG (left precentral gyrus) [mm]	thickness	-0.0479	0.035	-0.074	-0.022	-2.55	27195
L-PL (left paracentral lobule) [mm]	thickness	-0.0472	0.035	-0.075	-0.019	-2.83	27188
R-CMF (right caudal middle frontal gyrus) [mm]	thickness	-0.0392	0.035	-0.061	-0.017	-1.83	27268
L-CRBL-WM (left cerebellum white matter) [mm^3]	volume	-408.178	0.049	-668.75	-147.60	-4.39	26556
L-CRBL—L-SFG (rfMRI functional connectivity) [unitless]	AUC	-0.0175	0.030	-0.032	-0.003	-22.846	-

Supplemental Table 5. Associations between L-CRBL—L-SFG axis brain abnormalities and heart MRI-derived features available in UK Biobank (UKBB). Associations were computed among the UKBB PH subjects for which both brain and heart MRI were available. L-CRBL, left cerebellum; L-CRBL-WM, left cerebellar white matter; L-SFG, left superior frontal gyrus; LV, left ventricle; RV, right ventricle; PWA, pulse wave analysis; CI, confidence interval; Beta, regression coefficient; N, sample size; Padj, FDR-adjusted P value. Significance cutoff, $Padj \leq 0.05$; ns, $Padj > 0.05$, by ordinary least squares regression. Data provided in Excel format.

Supplemental Table 6. Associations between L-CRBL—L-SFG axis brain abnormalities and single nucleotide polymorphisms identified by previous genome-wide association studies. The associations were computed among the UK Biobank PH subjects for which both brain MRI and whole genome sequencing data were available. Additive genetic modeling was used. L-CRBL, left cerebellum; L-CRBL-WM, left cerebellar white matter; L-SFG, left superior frontal gyrus; SNP, single nucleotide polymorphism; GWAS, genome-wide association study; Beta, regression coefficient; P, nominal P value; Padj, Benjamini-Hochberg-adjusted P value; CI_025 and CI_975, lower and upper 95% confidence interval bounds; N, sample size. Significance cutoff, $Padj \leq 0.05$; ns, $Padj > 0.05$, by ordinary least squares regression. Data provided in Excel format.

Supplemental Table 7. Significantly altered brain structural regions and their functional connectivity in the UPMC PAH cohort (PAH: *N* = 10; matched Control: *N* = 10). Beta, regression coefficient; P, nominal P value; Padj, FDR-adjusted P value; CI_025 and CI_975, lower and upper 95% confidence interval bounds; N, sample size, and p-values are derived from covariate-adjusted ordinary least squares (OLS) regression with permutation-derived p-values. Significance cutoff, $P \leq 0.05$; ns, $P > 0.05$. Percent differences reflect raw group-level mean differences (unadjusted), included to aid interpretability of group-level magnitude. AUC (area under curve). See Figure 3, A–D for visualization of adjusted outcomes.

**IDP (image-derived phenotype) [unit]**	**Measure**	**Beta**	**P**	**CI 025**	**CI 975**	**Case – Control (%)**
L-SFG (left superior frontal gyrus) [mm]	thickness	-0.126	0.005	-0.198	-0.053	-3.90
L-CRBL-WM (left cerebellum white matter) [mm^3]	volume	-349.871	0.048	-699.895	-0.153	-0.18
L-CRBL—L-SFG (rfMRI functional connectivity) [unitless]	AUC	-0.223	0.0169	-0.223	-0.030	-39.411

Supplemental Table 8. Cognitive testing results between PH and control subjects across the UK Biobank (UKBB) and UPMC cohorts. In UKBB, FI13 (fluid intelligence score as the sum of number of correct answers to 13 questions, the higher the better), and MTM (mean time in milliseconds to correctly identify matches, where lower values reflect faster processing speed. Beta, regression coefficient; P, Padj, Benjamini-Hochberg-adjusted P value; CI_025 and CI_975, lower and upper 95% confidence interval bounds; N, sample size. Significance cutoff, $Padj \leq 0.05$; ns, $Padj > 0.05$, by ordinary least squares regression. In UPMC, FI (Flanker inhibitory control and attention test) is scored on a 0–100 scale, with higher scores indicating better cognitive performance. PC (pattern comparison processing speed test) is reported as an age-adjusted standard score (unitless), where higher values indicate faster processing speed. Significance cutoff, $P \leq 0.05$; ns, $P > 0.05$, by paired Student's t-test. Ctrl (control).

**UKBB**	**PH (mean ± sd)**	**Ctrl (mean ± sd)**	**Beta**	**Padj**	**CI 025**	**CI 975**	**N (PH)**	**N (Ctrl)**
FI13	5.79 ± 2.03	6.28 ± 2.1	-0.222	0.037	-0.407	-0.037	615	44416
MTM	572.69 ± 118.2	546.09 ± 107.6	5.705	0.046	0.105	11.305	1803	141484
**UPMC**	**PH (mean ± sd)**	**Ctrl (mean ± sd)**	**Paired t**	**Padj**	**CI 025**	**CI 975**	**N (PH)**	**N (Ctrl)**
FI	82.6 ± 3.5	95.2 ± 1.2	-12.621	0.025	-16.808	-8.434	12	12
PC	93.4 ± 4.8	106.3 ± 3.5	-12.962	0.041	-18.760	-6.481	12	12

Supplemental Table 9. UK Biobank comparator cohort statistics. IQR (interquartile range). Data provided in Excel format. N, sample size; BMI, Body Mass Index.

Supplemental Table 10. Altered brain structural regions and their functional connectivity across UK Biobank comparator cohorts. R- (right), L- (left), VDC (ventral diencephalon), THA (thalamus), PAL (pallidum), HIP (hippocampus), CRBL (cerebellum), WM (white matter), BS (brain stem), AMYG (amygdala), CHP (choroid plexus), ACC (accumbens), SPL (superior parietal lobe), IPL (inferior parietal lobule), SPM (supramarginal gyrus), SFG (superior frontal gyrus), RMFG (rostral middle frontal gyrus), PCNU (precuneus), PG (precentral gyrus), PSA (pars triangularis), PL (paracentral lobule), CMF (caudal middle frontal gyrus). CI (confidence interval). Significance cutoff, $Padj \leq 0.05$; ns, $Padj > 0.05$, by ordinary least squares regression and Benjamini-Hochberg adjustment. VDC, THA, PAL, HIP, CRBL, BS, AMYG, CHP, ACC and PCNU were measured in volume (mm^3); SPL, IPL, SPM, SFG, RMFG, PG, PSA, PL and CMF are in thickness (mm), functional connectivity is in AUC (area under curve) (unitless). Data provided in Excel format.

Supplemental Table 11. Associations between L-CRBL—L-SFG axis brain abnormalities and blood biochemical markers available in UK Biobank (UKBB). Associations were computed among the UKBB PH subjects for which both brain MRI and markers were available. L-CRBL, left cerebellum; L-CRBL-WM, left cerebellar white matter; L-SFG, left superior frontal gyrus; HbA1c, glycated hemoglobin; Beta, regression coefficient; P, nominal P value; Padj, Benjamini–Hochberg-adjusted P value; CI_025 and CI_975, lower and upper 95% confidence interval bounds; N, sample size; std, standard deviation. Significance cutoff, $Padj \leq 0.05$; ns, $Padj > 0.05$, by ordinary least squares regression via GLM with bootstrapped p-values. Data provided in Excel format.

Supplemental Table 12. Associations among L-CRBL—L-SFG axis brain abnormalities and plasma Olink proteomic data available in UK Biobank (UKBB). Associations were computed

among UKBB PH subjects for which both brain MRI and Olink data were available. Data are plotted in Supplemental Figure 3. L-CRBL, left cerebellum; L-CRBL-WM, left cerebellar white matter; L-SFG, left superior frontal gyrus; NPX, normalized protein expression; Beta, regression coefficient; P, nominal P value; Padj, Benjamini–Hochberg-adjusted P value; CI_025 and CI_975, lower and upper 95% confidence interval bounds; N, sample size. Significance cutoff, *Padj* ≤ 0.05; ns, *Padj* > 0.05, by ordinary least squares regression via GLM with bootstrapped p-values. Data provided in Excel format.

Supplemental Table 13. GO pathway enrichment analysis among Olink proteins significantly associated with L-CRBL—L-SFG axis brain abnormalities in the UK Biobank (UKBB) PH cohort. Proteins were binned into two sets with significant positive and negative associations, and pathway enrichment was performed for each set or both sets together. GO, Gene Ontology; L-CRBL, left cerebellum; L-CRBL-WM, left cerebellar white matter; L-SFG, left superior frontal gyrus; P, nominal P value; Padj, FDR-adjusted P value; FDR, false discovery rate. Overlap indicates the number of input proteins overlapping a GO term relative to the total number of proteins annotated to that term; odds ratio indicates enrichment strength; Genes lists the overlapping proteins. Significance cutoff, *Padj* ≤ 0.05; ns, *Padj* > 0.05, by enrichR and FDR-adjustment. Data provided in Excel format.

Supplemental Table 14. Hypoxia-related proteins available in the UK Biobank (UKBB) plasma Olink data. Association was tested for L-CRBL—L-SFG axis brain abnormalities within the UKBB PH cohort. Data provided in Excel format.

Supplemental Table 15. Plasma biochemical and Olink proteomic associations in LHD-associated and non-LHD PH strata in UK Biobank. PH subjects were stratified by the presence or absence of a left heart disease (LHD) diagnosis occurring before or at the time of PH diagnosis. Associations between PH status and blood biochemical markers or circulating Olink proteins were computed separately in LHD-associated and non-LHD PH strata using logistic regression. OR, odds

ratio; P, nominal P value; Padj, Benjamini–Hochberg-adjusted P value; OR_025 and OR_975, lower and upper 95% confidence interval bounds for OR; Total N, total sample size; Case N, number of PH cases; std, standard deviation. Significance cutoff, Padj ≤ 0.05; ns, Padj > 0.05, and Benjamini-Hochberg adjustment. Data provided in Excel format.

Supplemental Table 16. Pathway-level rs11154337_G–Olink associations in PH and comparator populations. Rank-based (Fast Gene Set Enrichment Analysis) FGSEA using the full rs11154337_G–Olink protein association spectrum within UK Biobank PH subjects and comparator populations. NES, normalized enrichment score; ES, enrichment score; P, nominal P value; Padj, FDR-adjusted P value; GO, Gene Ontology. AI (Autoimmune diseases), LHD (Left Heart Dysfunction), COPD (Chronic Obstructive Pulmonary Disease), ILD (Interstitial Lung Disease), SDB (Sleep Disordered Breathing), MD (Metabolic Disorders), RF (Renal Failure), HD (Hypertensive Diseases), DM (Diabetes Mellitus), AD (Anxiety Disorders), DD (Depression Disorders). CHD (Congenital Heart Disease), HIV (Human Immunodeficiency Virus) and UOVT (Use of oral Vasodilator therapy) did not have enough sample counts. Data provided in Excel format.

Supplemental Table 17. Associations between rs11154337 and L-CRBL—L-SFG axis measures in comparator populations. Associations between rs11154337 genotype and L-CRBL-WM volume, L-SFG thickness, and L-CRBL—L-SFG functional connectivity were tested in UK Biobank (UKBB) comparator populations using the same regression framework as in the PH cohort. L-CRBL, left cerebellum; L-CRBL-WM, left cerebellar white matter; L-SFG, left superior frontal gyrus; GLM, generalized linear model; Beta, regression coefficient; P, nominal P value; Padj, Benjamini–Hochberg-adjusted P value; CI_025 and CI_975, lower and upper 95% confidence interval bounds; N, sample size. Significance cutoff, Padj ≤ 0.05; ns, Padj > 0.05, by ordinary least squares regression via GLM with bootstrapped p-values and Benjamini-Hochberg adjustment. Data provided in Excel format.

Supplemental Table 18. Human PAH and control subjects providing cerebellar tissue.

Human Sample	Age	Sex	Diagnosis
Control 1	69	F	
Control 2	70	F	
Control 3	29	M	
PAH 1	68	F	Systemic Sclerosis
PAH 2	47	M	Portopulmonary Hypertension
PAH 3	54	F	Systemic Lupus Erythematosus
PAH 4	43	F	Systemic Lupus Erythematosus
PAH 5	81	M	Heritable PAH

Supplemental Table 19. Transthoracic echocardiography and right heart catheterization of WT and *Ncoa7-/-* mice. Cardiac structure, systolic function, and pulmonary hemodynamic parameters were assessed. Measurements include heart rate; left ventricular (LV) internal diameter, volume, and wall thickness at systole (s) and diastole (d); LV mass; stroke volume; ejection fraction; fractional shortening; cardiac output; right ventricular (RV) metrics; Fulton's index (RV/[LV + septum]); pulmonary artery acceleration time (PAT); pulmonary ejection time (PET); and pulmonary valve (PV) peak velocity. Values are shown for individual biological replicates. Statistical comparisons between groups were performed using age-adjusted linear models; both raw and age-adjusted, FDR-adjusted *P* values are reported. WT, wild type; P, P value; Padj, FDR-adjusted P value; N, sample size. Data provided in Excel format.

Supplemental Table 20. snRNA-seq cell-type composition analysis across WT, Il6 Tg (PH), and Il6 Tg;Ncoa7-/- (PH_KO) mouse cerebellum. Columns show the cell type, condition 1 (cond1), condition 2 (cond2), difference in mean cell-type percentage between groups (diff_percent), nominal P value from pairwise (p value), and Benjamini-Hochberg-adjusted P value (Adjusted p-value). Significance cutoff, Adjusted p_value ≤ 0.05; ns, Adjusted p_value > 0.05 by Student's t-test. Data provided in Excel format.

Supplemental Table 21. snRNA-seq differentially expressed genes (DEGs) identified across cerebellar cell types in *Il6* Tg (PH) vs WT and *Il6* Tg;*Ncoa7-/-* (PH_KO) vs *Il6* Tg (PH). Individual worksheet tabs labeled by cell type and comparison. Each sheet reports the gene symbol, nominal P value (p_val), percentage of expressing cells in group 1 (pct.1), percentage of expressing cells in group 2 (pct.2), Benjamini-Hochberg-adjusted P value (p_val_adj), and average log2 fold change (avg_log2FC). Significance cutoff, p_val_adj≤ 0.05; ns, p_val_adj > 0.05. Data provided in Excel format.

Supplemental Table 22. snRNA-seq DEG-based GSEA across cerebellar cell types in *Il6* Tg (PH) vs WT and *Il6* Tg;*Ncoa7-/-* (PH_KO) vs *Il6* Tg (PH). Individual worksheet tabs labeled by cell type, comparison, and up- or down-differentially expressed genes (DEGs) used for gene set enrichment analysis (GSEA). Each sheet reports enriched Gene Ontology Biological Process (GOBP) terms, gene-set overlap, nominal P value, adjusted P value, odds ratio, combined enrichment score, and the contributing genes driving each enriched pathway. Significance cutoff, Adjusted P value ≤ 0.05; ns, Adjusted P value > 0.05. Data provided in Excel format.

Supplemental Table 23. snRNA-seq stepwise DEGs across cerebellar Granule_main, MLI1, and MLI2 cell types in *Il6* Tg (PH) vs WT and *Il6* Tg;*Ncoa7-/-* (PH_KO) vs *Il6* Tg (PH) mice. Stepwise increased differentially expressed genes (DEGs) were defined as genes increased in *Il6* Tg vs WT and further increased in *Il6* Tg;*Ncoa7-/-* vs *Il6* Tg, whereas stepwise decreased DEGs were defined as genes decreased in *Il6* Tg vs WT and further decreased in *Il6* Tg;*Ncoa7-/-* vs *Il6* Tg. Each sheet reports the gene symbol, *Il6* Tg vs WT log-fold change (FC), *Il6* Tg;*Ncoa7-/-* vs *Il6* Tg logFC, approximate *Il6* Tg;*Ncoa7-/-* vs *Il6* Tg logFC, nominal P values (p_val), and adjusted P values (p_val_adj) for both pairwise comparisons. Purkinje cells and microglia did not contain genes meeting criteria for stepwise DEGs. Significance cutoff, p_val_adj≤ 0.05; ns, p_val_adj > 0.05. Data provided in Excel format.

Supplemental Table 24. snRNA-seq stepwise DEG-based GSEA across cerebellar cell types in *Il6* Tg (PH) vs WT and *Il6* Tg;*Ncoa7-/-* (PH_KO) vs *Il6* Tg (PH) mice. Individual worksheet tabs labeled by cell type, comparison, and stepwise increase- or decrease-differentially expressed genes (DEGs) used for gene set enrichment analysis (GSEA). Each sheet reports enriched Gene Ontology Biological Process (GOBP) terms, gene-set overlap, nominal P value, adjusted P value, odds ratio, combined enrichment score, and the contributing genes driving each enriched pathway. MLI2 did not

have any enriched pathway. Significance cutoff, Adjusted P value ≤ 0.05; ns, Adjusted P value > 0.05. Data provided in Excel format.

Supplemental Table 25. Spatial transcriptomic cell-type composition analysis based on RCTD-estimated whole-section cell-type fractions across WT, *Il6* Tg (PH), and *Il6* Tg;*Ncoa7-/-* (PH_KO) mouse cerebellum. Columns show the cell type, condition 1 (cond1), condition 2 (cond2), difference in mean estimated fraction between groups (diff_mean), nominal P value (p_value), and FDR-adjusted P value (Adjusted p_value). Significance cutoff, Adjusted p_value ≤ 0.05; ns, Adjusted p_value > 0.05 by Wilcoxon rank sum test. Data provided in Excel format.

Supplemental Table 26. Spatial, most-enriched cell-type-targeted DEGs across each cerebellar spatial cluster in *Il6* Tg (PH) vs WT and *Il6* Tg;*Ncoa7-/-* (PH_KO) vs *Il6* Tg (PH) mice. Individual worksheet tabs are labeled by spatial cluster, comparison, and differentially expressed gene (DEG) direction. DEGs were identified within each spatial cluster after excluding the dominant Robust Cell Type Decomposition (RCTD)-inferred cell-type fraction and adjusting for the indicated non-dominant local cell-type fractions as latent variables. Each sheet reports the gene symbol, number of spots in each comparison group (n_ident_spots), excluded dominant cell-type fraction, latent variables used (latent_vars_used), nominal P value (p_val), average log2 fold change (avg_log2FC), percentage of expressing spots (pct) in each group, and adjusted P value (p_val_adj). Significance cutoff, p_val_adj≤ 0.05; ns, p_val_adj > 0.05. Data provided in Excel format.

Supplemental Table 27. Spatial, most-enriched cell-type-targeted DEG-based GSEA across each cerebellar spatial cluster in *Il6* Tg (PH) vs WT and *Il6* Tg;*Ncoa7-/-* (PH_KO) vs *Il6* Tg (PH) mice. The file contains two worksheets, *Il6* Tg vs WT and *Il6* Tg;*Ncoa7-/-* vs *Il6* Tg. For each comparison, enriched Gene Ontology Biological Process (GOBP) terms are reported by spatial cluster and differentially expressed gene (DEG) direction (direction), together with the assigned major

biological classification module (module), gene-set overlap, nominal P value (p_value), adjusted P value (adjusted_p_value), odds ratio, combined enrichment score, and contributing genes driving each enriched pathway. Adjusted P value ≤ 0.05; ns, Adjusted P value > 0.05. Data provided in Excel format.

Supplemental Table 28. Spatial, microglia-targeted DEGs across each cerebellar spatial cluster in *Il6* Tg (PH) vs WT and *Il6* Tg;*Ncoa7-/-* (PH_KO) vs *Il6* Tg (PH) mice. Individual worksheet tabs are labeled by spatial cluster, comparison, and differentially expressed gene (DEG) direction. DEGs were identified within each spatial cluster after excluding Robust Cell Type Decomposition (RCTD)-inferred microglia fraction and adjusting for the indicated local cell-type fractions as latent variables. Each sheet reports the gene symbol, number of spots in each comparison group (n_ident_spots), excluded dominant cell-type fraction (excluded_dominant_fraction), latent variables used (latent_vars_used), nominal P value (p_val), average log2 fold change (avg_log2FC), percentage of expressing spots in each group (pct), and adjusted P value (p_val_adj). Significance cutoff, p_val_adj≤ 0.05; ns, p_val_adj > 0.05. Data provided in Excel format.

Supplemental Table 29. Spatial, microglia-targeted DEG-based GSEA across each cerebellar spatial cluster in *Il6* Tg (PH) vs WT and *Il6* Tg;*Ncoa7-/-* (PH_KO) vs *Il6* Tg (PH) mice. Individual worksheet tabs are labeled by spatial cluster, comparison, and differentially expressed gene (DEG) direction. Reported pathways represent microglia-targeted enrichments retained after removing pathways also enriched in the corresponding dominant cell type-targeted DEG-based gene set enrichment analysis (GSEA). Each sheet reports enriched Gene Ontology Biological Process (GOBP) terms, gene-set overlap, nominal P value, adjusted P value, odds ratio, combined enrichment score, and the contributing genes driving each enriched pathway. Adjusted P value ≤ 0.05; ns, Adjusted P value > 0.05. Data provided in Excel format.

Supplemental Table 30. Antibodies used for immunofluorescent (IF) staining of explanted human lungs and mouse tissues. Primary antibodies are listed by target organism, antibody name, catalog number, vendor, application, and dilution. Fluorophore-conjugated or species-appropriate secondary antibodies were used to detect unconjugated primary antibodies.

Target organism	Primary Antibodies	Catalog No.	Vendor	IF	Dilution
Human, Mouse	NCOA7-594	sc-393427 AF594	Santa Cruz Biotechnology	IF	1:25
Human, Mouse	Goat anti-Calbindin	PA5-46936	Themo Fisher Scientific	IF	1:100
Human, Mouse	Goat anti-NeuN	ab236870	Abcam	IF	1:100
Human, Mouse	Chicken anti-Parvalbumin	PA5-143579	Themo Fisher Scientific	IF	1:100
Human, Mouse	Rabbit anti-IBA1	17198S	Cell Signaling Technology	IF	1:200
Human	Goat anti-CD31	AF3628	R&D	IF	1:100
Human	Goat anti-GFAP	PA5-143587	Themo Fisher Scientific	IF	1:100
Human	Chicken anti-MBP	SKU: MBP	Antibodies	IF	1:25
Mouse	Rabbit anti-CH25H	PA5-72349	Invitrogen	IF	1:200
	Rabbit anti-NCOA7	23092-1-AP	Proteintech	IB	1:1000
	Anti-β-Actin−Peroxidase antibody	A3854	Sigma Aldrich	IB	1:5000
**Target organism**	**Secondary antibodies**	**Catalog No.**	**Company**	**IF/IB**	**Dilution**
None	Donkey anti-Goat IgG (H+L) Highly Cross-Adsorbed Secondary Antibody, Alexa Fluor™ Plus 488	A32814	Themo Fisher Scientific	IF	1:1000
None	Donkey anti-Goat IgG (H+L) Highly Cross-Adsorbed Secondary Antibody, Alexa Fluor™ Plus 647	A32849TR	Themo Fisher Scientific	IF	1:1000
None	Donkey anti-Rabbit IgG (H+L) Highly Cross-Adsorbed Secondary Antibody, Alexa Fluor™ 488 - A-21206	A-21206	Themo Fisher Scientific	IF	1:1000
None	Donkey anti-Rabbit IgG (H+L) Highly Cross-Adsorbed Secondary Antibody, Alexa Fluor™ Plus 647 - A32795TR	A32795TR	Themo Fisher Scientific	IF	1:1000
None	Alexa Fluor® 488 AffiniPure® Donkey Anti-Chicken IgY (IgG) (H+L)	703-545-155	Jackson ImmunoResearch	IF	1:1000
None	Anti-rabbit IgG, HRP-linked Antibody	7074	Cell Signaling Technology	IB	1:5000

Supplemental Methods

UK Biobank data

The United Kingdom Biobank (UKBB) is a large prospective population of more than 500,000 participants aged 37–73 years, recruited between 2006 and 2010. Participants were recruited from a range of backgrounds and attended one of 22 assessment centers where they completed baseline touchscreen questionnaires on sociodemographic features, behavior, lifestyle, mental health, and several cognitive tests. Blood, urine and saliva samples were collected, and physical measurements were taken. In addition, UKBB aims to acquire longitudinal neuroimaging data for ca. 100,000 participants with ~45,000 separate MRI sessions for ~40,000 participants released so far. This research has been conducted using the UK Biobank Resource under UPMC Application number: 48054.

UPMC data

PAH patients were enrolled at UPMC from 2017–2018. All patients received care through the Comprehensive Pulmonary Hypertension Program (CPHP) and were recruited from the UPMC PH Registry/CPHP using stringent eligibility criteria to establish a clinically homogeneous validation cohort with moderate PAH severity and limited comorbidity burden (Supplemental Figure 2, A and B). All diagnoses were based on expert physician assessment and right heart catheterization data. This design excluded patients with advanced hemodynamic failure, markedly reduced cardiac output, or those requiring supplemental oxygen, to minimize the possibility that advanced RV failure, low-output physiology, or end-stage cardiopulmonary disease would independently drive neurocognitive or brain imaging abnormalities. Thus, the UPMC cohort was not selected to represent the full hemodynamic spectrum of PAH, but rather to evaluate brain structural and functional changes in PAH without major confounding from advanced cardiac decompensation.

PAH cases were matched 1:1 with non-PAH healthy controls by age, sex, and education level. Participants completed NIH Toolbox cognitive testing before T1-weighted structural MRI and resting-state fMRI. Controls had no cardiovascular or pulmonary disease history or risk factors and were excluded for excess alcohol use, sleep apnea symptoms, cardiac/pulmonary medication use, relevant personal history, or family history of pulmonary hypertension or scleroderma (Supplemental Figure 2B). PAH demographic, hemodynamic, medication, and functional data were manually curated from UPMC EHR records. Comorbidity burden was minimal, with only one PAH participant having essential hypertension and none having diabetes, coronary artery disease, or prior stroke.

UK Biobank cohort definition

***PH cohort*:** The population of PH subjects from UKBB was algorithmically defined by using an International Classification of Disease 9th and 10th Revisions (ICD-9, ICD-10) for PH, in combination with ICD codes representing diagnoses commonly associated with PH, treatment/medications codes relevant to PH and associated diseases and Office of Population Censuses and Surveys Classification of Interventions and Procedures, version 4 (OPCS-4) codes (1). Further characterization of PH subjects into WHO groups was limited as invasive cardiac monitoring data were not part of the UKBB protocol. Subjects without an MRI scan at Instance 2 or with a PH diagnosis date occurring after the MRI scan were excluded (1-4), (https://www.aapc.com/blog/49254-4-tips-for-accurate-pulmonary-hypertension-coding/). All the used ICD/OPSC codes are listed in Supplemental Table 1. Cognitive test performance of PH subjects was analyzed using the baseline (Instance 0) assessment data.

***Control cohort*:** To define the healthy control cohort we first excluded any participants who answered ‘yes’ or ‘preferred not to answer’ to the question ‘Do you have “X” condition as diagnosed by a physician?’ (Data-Field 20003) (Supplemental Figure 1). The ICD-9/10 codes for the remaining participants were then reviewed and the following codes were also excluded: malignant neoplasm,

chronic infections, systemic endocrine disorders, neurologic and psychiatric disorders, disease of the cardiac or respiratory system, chronic oral infection, cirrhosis, inflammatory rheumatologic conditions, maternal diseases complicating pregnancy, brain injury and organ transplant (Supplemental Figure 1; Supplemental Table 1). These codes were selected for exclusion because they could serve as potential confounders of brain MRI outcomes, represent risk factors for PH, or indicate conditions associated with chronic inflammation. Oral infections were excluded because of their anatomical proximity to the brain, their association with vascular inflammation, and their link to increased risk of cardiac and respiratory all-cause mortality (5). The ICD-9/10 codes of the remaining participants were adjudicated by a two-physician panel for consensus opinion so that the 'generally healthy' control cohort represented participants without chronic disease diagnosis or carrying a self-limited medical illness or infection, non-brain trauma, non-rheumatologic orthopedic injuries or nonspecific symptoms without a clear diagnosis.

Comparator cohorts: The comparator cohorts were derived by medical conditions associated with increased risk of PAH (congenital heart disease (CHD), autoimmune diseases (AI), HIV, liver disease (LD), use of methamphetamine (UM)) or clinically common conditions observed in PH patients (left heart dysfunction (LHD), chronic obstructive pulmonary disease (COPD), interstitial lung disease (ILD), sleep disordered breathing (SDB), metabolic disease (MD), renal failure (RF), deep venous thrombosis (DVT), pulmonary embolism (PE), hypertensive disorders (HD), diabetes mellitus (DM), anxiety disorders (AD), and depression disorders (DD)). A population receiving oral cyclic guanosine monophosphate phosphodiesterase (cGMP) inhibitor therapy (PDE5Is) (use of oral vasodilator therapy (UOVT)) was also identified to address the potential confounding impact of vasodilatory therapy on brain structure and function in PH patients. These cohorts were curated from the general UKBB population first by using a combination of relevant ICD-9/10, OPCS and treatment/medication codes (Supplemental Table 1). Subsequently, any participant without a baseline brain MRI scan was excluded. The remaining subject IDs were cross-referenced with the PH cohort, and any overlaps

were excluded to ensure that the comparator cohorts were free from any PH influence. Due to the small sample sizes (< 10), LD, UM, DVH and PE were excluded from further analyses.

MRI data acquisition and processing

UKBB cohort*:* A detailed description of the MRI data acquisition and preprocessing is described in (6). Briefly, 3T Siemens Skyra scanners with the standard Siemens 32-channel head coil were used for acquiring both T1w structural and rfMRI brain images. T1w-MRI scans were acquired using a 3D MPRAGE acquisition at 1 mm isotropic resolution in 208x256x256 matrix with a 256 mm superior-inferior field of view (6). The raw images were processed using FSL (7), which included defacing, gradient distortion correction (GDC), skull stripping, and both linear (FLIRT) and nonlinear (FNIRT (https://www.fmrib.ox.ac.uk/datasets/techrep/tr07ja2/tr07ja2.pdf)) registration to the MNI152 standard space. Brain segmentation and volume estimation were conducted using FreeSurfer (7-10).

rfMRI scans were acquired using a multi-band gradient echo EPI sequence, obtaining a total of 490 timepoints in approximately 6 minutes. The spatial resolution was 2.4 mm isotropic voxels in 88x88x64 matrix with acquisition parameters of TE/TR = 39/0.735 s, multi-band factor of 8, no in-plane acceleration, and a flip angle of 52 degrees. The raw images were processed in the following way: motion correction using MCFLIRT, grand-mean intensity normalization, high-pass temporal filtering, EPI unwarping, GDC unwarping, and removal of remaining structured artefacts by ICA+FIX (8, 11-13).

UPMC cohort: T1w-MRI scans were acquired by 3T Siemens Skyra scanners with the 64-channel head coil. Images were acquired using a 3D MPRAGE acquisition at 1 mm isotropic resolution in a 208x256x256 matrix with a 256 mm superior-inferior field of view. For the image analysis, we followed the protocol described in (14). Briefly, FSL was first used to perform defacing, field map correction, alignment to the Anterior Commissure–Posterior Commissure line (ACPC), and skull stripping. Then

FreeSurfer was used for brain segmentation, registration to the MNI152 standard space, and volume and thickness estimation.

rfMRI scans were acquired using a multi-band gradient echo EPI sequence, obtaining a total of 480 time points in approximately 8 minutes. The spatial resolution was 2.0 mm isotropic voxels in a 110x110x60 matrix with acquisition parameters of TE/TR = 30ms/1.0 s, a multi-band factor of 5, no in-plane acceleration, and a flip angle of 55 degrees. The raw images were first processed with FSL, including field map correction. Then, we performed the alignment of images to corresponding T1w space, BOLD slice time correction and motion correction. ICA+FIX was run to remove remaining structured artifacts.

Brain structural volume analysis

The UKBB populations were used as the unbiased discovery cohorts. Structural IDPs of interest were obtained from the FreeSurfer default aparc+aseg brain atlas. The case-versus-control difference in the cross-sectional IDP was modeled using an Ordinary Least Squares (OLS) regression model as a function of the case-versus-control binary variable, age, sex, education, smoking history, BMI, and total intracranial volume (TIV). By convention, analysis of neurocognitive and cardiopulmonary data includes adjustment for education, smoking and BMI, thus its inclusion in this model, therefore, these variables were included as covariates. Our model was further adjusted for common medical conditions that can confound structural brain imaging, namely, essential hypertension (EH), ischemic and hemorrhagic stroke (IHS) and diabetes with and without complications (Dww/oC) to account for potential confounding by co-existent disease within the PH cohort. We encoded them as binary variables where 1 indicates the previous diagnosis for that condition. Thus, the model equation is:

$$\text{IDP} = \text{Case-versus-control} + \text{Age} + \text{Sex} + \text{Education} + \text{Smoked} \\ + \text{BMI} + \text{TIV} + \text{EH} + \text{IHS} + \text{Dww/oC} \qquad (1)$$

where Education and Smoked are binary variables with 1 indicating the attainment of a college degree and past smoking history, respectively. Regression was performed with the ols function of the statsmodels Python package. The Benjamini-Hochberg multiple test correction was applied with the adjusted p-value cutoff at ≤ 0.05. A multivariate regression approach was used instead of case-control matching due to the need for a high number of covariate adjustments to account for the high population heterogeneity in UKBB. This approach is favorable in general over case-control matching due to larger sample sizes and decreased variance, as well as increased robustness to repeated and a different set of controls (15).

The validation analysis in the UPMC PAH cohort was conducted for the brain regions with significant volume alterations in the UKBB PH cohort. Case data were compared to age-, sex-, and education-matched non-PAH controls in a 1:1 ratio using OLS regression with a clustered sandwich estimator, adjusting for total brain volume as a covariate. Given the small sample size and high inherent noise in MRI data, p-values were estimated via a random permutation test. In this approach, case-control pairs were randomly reassigned while preserving each sample's class (i.e., case or control) to maintain the 1:1 matching ratio. The OLS regression was then applied to each permutation. This procedure was repeated 1,000 times, and the proportion of permutation-derived p-values less than the originally observed p-value was used as the final estimated p-value. Since this was a hypothesis-driven test of a small number of significantly altered brain regions (≤ 5) in the UKBB PH cohort, multiple test correction was not applied to ensure more interpretability and avoid unnecessary Type II errors (16-19).

Visualization of brain maps was performed using BrainNetViewer (20).

rfMRI brain functional connectivity analysis

The UKBB cohort was used to identify significant changes in functional connectivity among regions with significant volume alterations identified by the T1w-MRI analysis. For each pair of regions, the Pearson correlation coefficient was calculated across all time points in the rfMRI data. Each weighted

connectivity graph was then transformed into a binary graph by applying a threshold to the absolute correlation coefficient. Functional connections exceeding this threshold were considered present. As the overall connectivity strength varies with individuals as well as disease conditions, it is common practice to set an individual-specific threshold to maintain uniform graph density, thereby ensuring comparability. Instead of applying a single arbitrary graph density threshold, we implemented a range of thresholds from 0.1 to 0.5, in increments of 0.05, following the previously established method (21), *i.e.,* Graph density 0.1 means maintaining top 10% strongest rfMRI functional connections, 0.2 means top 20%, and so on. Two graph theoretical measures were computed for each region; clustering coefficient (transitivity) $\frac{2t_v}{k_v(k_v-1)}$, and betweenness centrality (https://www.sciencedirect.com/science/article/pii/0378873378900217) $\sum_{s,t} \frac{SP_{st}(v)}{SP_{st}}$, where v is a region of interest, t_v is the number of triangles around v, SP_{st} is the number of shortest paths from regions s to t, and $SP_{st}(v)$ is the number of shortest paths from s to t going through v, and were integrated over the above-mentioned density threshold ranges to compute the area under the curve (AUC). In addition, the AUC for each functional connectivity strength (absolute correlation coefficient) was also computed. The AUC calculation was done using the AUC function implemented in the DescTools R package. Then, OLS regression was carried out using model equation (1) where IDP is the AUC of each metric. Given that the AUC is not normally distributed, the p-value was estimated using a random permutation approach. Here, case and control labels were randomly reassigned while preserving the original sample sizes. OLS regression was then applied to each permutation. This process was repeated 10,000 times, and the proportion of permutation-derived regression β coefficients that were either less than or greater than the originally observed β (whichever smaller proportion) was used as

the final estimated p-value. As this was a hypothesis-driven test of a small number of significantly altered brain regions (≤ 3) in the UKBB PH cohort, multiple test correction was not applied.

The significant change in AUC of each functional connectivity strength observed in the UKBB PH cohort was validated in the UPMC PAH cohort. The same OLS regression and permutation-based p-value estimation used in the structural MRI analysis were applied here, with the same age-, sex-, and education-matched PAH and non-PAH controls in a 1:1 ratio. Since only one significant functional connectivity change was identified in the UKBB PH cohort, no adjustment for multiple testing was applied. The significance threshold was set at p-value ≤ 0.05.

Blood biomarker and Olink proteomics association analysis

The statistical associations between the UKBB blood biochemical markers (Category: 17518) and Olink proteomics data (Data-Field: 30900), and L-CRBL-WM volume, L-SFG thickness and L-CRBL—L-SFG connectivity strength among PH subjects were computed by modified model equation (1):

$$\begin{aligned} \mathrm{IDP} = {} & \mathrm{ProteinExpression} + \mathrm{Age} + \mathrm{Sex} + \mathrm{Education} + \mathrm{Smoked} \\ & + \mathrm{BMI} + \mathrm{TIV} + \mathrm{EH} + \mathrm{IHS} + \mathrm{Dww/oC} + \mathrm{TimeOffset} \end{aligned} \qquad (2)$$

where the new terms ProteinExpression and TimeOffset are the protein expression data, and the time difference between the protein expression measurement date and MRI measurement date, respectively. All plasma protein analyses were conducted within disease-defined cohorts rather than pooled case–control samples, to ensure that estimated associations reflected within-disease biological variability and were not driven by between-group differences in comorbidity prevalence. The latter term was introduced since the protein samples were obtained at UKBB instance 0 whereas the MRI data acquisition was conducted at instance 2. To account for the differences in numerical scales between these two terms, they were standardized across samples prior to the regression analysis. Regression was performed with the glm function of the statsmodels Python package using the

Gaussian conditional distribution family. Given the high variability in the estimated regression coefficients and the relatively small sample size compared to the number of covariates, confidence intervals were estimated using a bootstrapping approach. In each iteration, samples were randomly resampled with replacement to match the original sample size, and regression analysis was conducted. This process was repeated 1,000 times to generate confidence intervals and estimate the p-value for the ProteinExpression variable's β coefficient. The smaller proportion of bootstrapped βs that were either less than or greater than the originally observed β was used as the final estimated p-value. The Benjamini-Hochberg multiple test correction was applied with the adjusted p-value cutoff at ≤ 0.05.

Significantly associated Olink proteins were used as input gene sets for the enrichR R package (22) using the "GOBP_2023" database. The FDR adjusted p-value significance cutoff was set to < 0.05. Hypoxia-related proteins were manually curated from (23, 24) along with canonical hypoxia inducible factors (Supplemental Table 14).

SNP association analysis

Individual SNP data were obtained from UKBB. The association between a SNP and L-CRBL-WM volume, L-SFG thickness and L-CRBL—L-SFG connectivity strength among UKBB PH subjects was computed by modified model equation (1):

$$\begin{aligned} \text{IDP} = \text{SNP} + \text{Age} + \text{Sex} + \text{Education} + \text{Smoked} \\ + \text{BMI} + \text{TIV} + \text{EH} + \text{IHS} + \text{Dww/oC} \end{aligned} \tag{3}$$

where the SNP term takes on three discrete values representing the allele combination (i.e. additive model). In the case of rs11154337, it is either C/C, G/C, or G/G encoded as 0, 1, and 2, respectively. Regression was performed with the ols function of the statsmodels Python package. Subsequent rank-

based FGSEA was performed using the FGSEA R package (https://bioconductor.org/packages/release/bioc/html/fgsea.html).

MRI data acquisition, processing and analysis of *ex vivo* mouse brain

We followed our established protocol (25). Mice were anesthetized with 3% isoflurane, and transcardially perfused with 4% paraformaldehyde (PFA) and decapitated. Brains were maintained within the skull to avoid anatomical deformation and were fixed in 4% PFA for 48h, then stored in PBS solution at 4 °C. MRI was performed at 500 MHz using a Bruker AV3HD 11.7 T/89 mm vertical bore small animal MRI scanner, equipped with a 20-mm quadrature radiofrequency (RF) coil and Paravision 6.0.1 software (Bruker Biospin, Billerica, MA). Following positioning and pilot scans, T2w images were acquired using a Rapid Acquisition with Relaxation Enhancement (RARE) sequence, with the following parameters: Echo Time/Repetition Time (TE/TR) = 20/4000 ms, averages = 8, 160 × 160 matrix, 25 slices with a 0.5 mm slice thickness, a RARE factor = 4, and a field of view (FOV) of 16 × 16 mm.

The raw images were converted into NIFTI format. Skull stripping was manually performed using ITK-SNAP (26). Registration of raw images to the *ex vivo* NeAt brain atlas (27, 28) was carried out using FSL (29) and ANTs (30). Cerebellar volume was quantified with voxels in sample native space. Differential volume analysis among three mouse model types (WT, *Il6* Tg and *IL6* Tg; *NCOA7*-/-) was performed by using an OLS model with age and total brain volume (TBV) as covariates. Due to the unmatched, small sample size, we first adjusted for TBV following the approach by (31-33):

$$V_{adjusted} = V_{raw} - \beta_{WT}\left(TBV_{raw} - TBV_{WTmean}\right)$$

where V is cerebellar volume, β_{WT} is estimated by:

$$V_{rawest} = \beta_{WT} \left(TBV_{raw} - TBV_{WTmean} \right)$$

Then, the OLS coefficient and p-value for sample treatment difference were estimated against $V_{adjusted}$ using age as the only covariate. As in the human case, due to the small sample size and low signal-to-noise ratio in MRI data, p-values were estimated via a random permutation test. Sample labels were randomly reassigned while preserving the original sample sizes. OLS regression was then applied to each permutation, and this process was repeated 1,000 times. The final estimated p-value was determined by calculating the proportion of permutation-derived regression β coefficients that were either less than or greater than the originally observed β (the smaller one was used).

Cognitive test analysis

UKBB cognitive function summary data (Category: 1005) were analyzed for their associations with PH diagnosis using model equation (1) but without TIV, where IDP was replaced with each of the summary measures. To ensure the maximum available sample numbers, we used the Instance 0 (initial assessment visit) samples for this analysis. The Benjamini-Hochberg multiple test correction was applied with the adjusted p-value cutoff at ≤ 0.05.

The validation analysis in the UPMC PAH cohort focused on tests that showed significant changes in the UKBB PH cohort. Although identical tests were not available in the UPMC cohort, the closest comparable test types (Flanker inhibitory control test, composite of fluid cognition, and pattern comparison processing speed test) were selected for validation purposes. Age-, sex-, and education-matched non-PAH control data were also obtained in a 1:1 case-control ratio. Significance was assessed using a paired *t*-test. As in the UPMC T1w-MRI analysis, this analysis involved a small number of hypothesis-driven tests (≤ 3), so multiple test correction was not applied.

Cerebellar immunofluorescence

For human tissue studies, in accordance with federal regulations for the protection of human subjects (45 CFR 46), deidentified human cerebellar tissue sections were obtained through a departmental honest broker from the UCLA Translational Pathology Core Laboratory (IRB#11-002504). Paraffin-embedded human cerebellar sections (5 μm thickness) were deparaffinized, rehydrated, and subjected to heat-induced antigen retrieval using citrate buffer (pH 6.0; C9999, Sigma-Aldrich). For mouse tissue studies, OCT-embedded brain sections were washed three times with PBS to remove residual embedding medium and subsequently subjected to heat-induced antigen retrieval using the same citrate buffer (pH 6.0; C9999, Sigma-Aldrich). Following antigen retrieval, tissue sections were permeabilized with 0.2% Triton X-100 in PBS for 30 min at room temperature and then blocked in PBS containing 5% donkey serum (D9663, Sigma Aldrich) for 1 h. Sections were incubated overnight at 4°C with primary antibodies (Supplemental Table 30) diluted in blocking buffer. After three washes with PBS with 0.05% Tween 20 (PBST), sections were incubated with species-appropriate fluorescent secondary antibodies for 1 h at room temperature. Following three additional washes with PBS, nuclei were counterstained with DAPI (62248, Thermo Fisher Scientific) for 10 min. Sections were mounted using Fluoromount-G™ Mounting Medium (00-4958-02, Thermo Fisher Scientific) and coverslipped. Fluorescence images were acquired using a Nikon A1 confocal microscope (Nikon Instruments, Tokyo, Japan). Quantitative image analyses were performed using ImageJ software (National Institutes of Health, Bethesda, MD, USA). Details of primary and secondary antibodies are provided in Supplemental Table 30.

ELISA

Human control and PAH plasma samples were obtained from the UPMC; detailed patient information is provided in Supplemental Table 3. Plasma levels of CX3CL1 (DCX310, R&D Systems), CFD (DFD00, R&D Systems), progranulin (DPGRN0, R&D Systems), and C1QBP (NBP3-39916, Novus Biologicals) in control and PAH subjects were measured using commercially available ELISA kits according to the manufacturers' instructions.

Cell culture and treatment

HMC3 cells (CRL-3304, ATCC) were cultured in Eagle's Minimum Essential Medium (EMEM) supplemented with 10% fetal bovine serum (FBS). For NCOA7 silencing using shRNA lentiviral particles, HMC3 cells were seeded in 6-well plates and cultured to approximately 50% confluency. Cells were transduced with either control shRNA lentiviral particles (shcontrol; sc-108080, Santa Cruz Biotechnology) or NCOA7-targeting shRNA lentiviral particles (shNCOA7; sc-95482-V, Santa Cruz Biotechnology) in the presence of 5 µg/mL polybrene (sc-134220, Santa Cruz Biotechnology). After transduction, the viral-containing medium was removed and replaced with complete growth medium without polybrene. Three days post-transduction, cells were split at a 1:3 ratio into fresh complete medium and cultured for an additional 2 days. For selection of transduced cells, puromycin dihydrochloride (3 µg/mL; sc-108071, Santa Cruz Biotechnology) was added to eliminate non-transduced cells. Experiments were conducted after puromycin selection. For NCOA7 silencing using siRNA, HMC3 cells were transfected at approximately 70–80% confluency with either negative control siRNA (siNC) (AM4635, Thermo Fisher Scientific) or NCOA7-targeting siRNA (siNCOA7) (4392421, ID: s43908; Thermo Fisher Scientific). Transfections were performed using Lipofectamine 2000 (11668019, Thermo Fisher Scientific), with siRNA complexes prepared in Opti-MEM reduced-serum medium according to the manufacturer's instructions. Experiments were conducted for 48 hours post-transfection.

SiR-Lysosome assay

As previously described (34), the SiR-Lysosome assay (CYSC012, Cytoskeleton), a cell-permeable peptide conjugated to a silicon rhodamine (SiR) dye, was used to assess active cathepsin D in HMC3 cells. Cells were incubated with SiR-Lysosome at a final concentration of 1 µM in the presence of the calcium channel blocker verapamil (10 µM) to enhance signal intensity for 30 min at 37 °C. Cells were then analyzed by flow cytometry and fluorescence microscopy. For flow cytometric analysis, cell

suspensions were analyzed using an LSR flow cytometer (Becton Dickinson), and data were processed with FlowJo software. For confocal microscopy, cells were rinsed three times with ice-cold PBS, fixed in 4% paraformaldehyde (PFA), and imaged using a confocal microscope.

Lysosomal acidification assay

HMC3 with different treatment were incubate 1 μM LysoSensor™ Green DND-189 (L7535, Thermo Fisher Scientific) for 1.5 hour following the previous approach (34). Cells were then analyzed by flow cytometry and fluorescence microscopy. For flow cytometric analysis, cell suspensions were analyzed using an LSR flow cytometer (Becton Dickinson), and data were processed with FlowJo software (Tree Star). For confocal microscopy, cells were rinsed three times with ice-cold PBS, fixed in 4% PFA, and imaged using a confocal microscope.

Lysosomal lipid content assay

Following the previous approach (34), HMC3 with different treatments were incubate 2.5 μM BODIPY for 2 hours. Cells were then analyzed by flow cytometry and fluorescence microscopy. For flow cytometric analysis, cell suspensions were analyzed using an LSR flow cytometer (Becton Dickinson), and data were processed with FlowJo software. For confocal microscopy, cells were rinsed three times with ice-cold PBS, fixed in 4% paraformaldehyde (PFA), and imaged using a confocal microscope.

Phagocytosis assay

Mouse brain synaptosomes were isolated according to the established protocol (35) and labeled with pHrodo™ dye (P36012, Thermo Fisher Scientific). HMC3 cells were treated with the labeled synaptosomes at a final concentration of 1 μg per 100 μL for 3 hours. At the end of the treatment, the culture medium was removed, and cells were washed three times with PBS to remove non-internalized particles. Cells were then analyzed by flow cytometry and fluorescence microscopy. For

flow cytometric analysis, cell suspensions were analyzed using an LSR flow cytometer, and data were processed with FlowJo software. For confocal microscopy, cells were rinsed three times with ice-cold PBS, fixed in 4% PFA, and imaged using a confocal microscope.

Immunoblotting

Cells were lysed in RIPA buffer (89901, Thermo Fisher Scientific) supplemented with a protease and phosphatase inhibitor cocktail (78441, Thermo Fisher Scientific). The lysates were centrifuged at 12,000 × g for 10 minutes at 4°C, and the supernatants were collected. Protein concentrations were determined using the BCA Protein Assay Kit (23227, Thermo Fisher Scientific) to ensure equal loading across samples. Proteins were mixed with loading buffer, separated on 12% TGX polyacrylamide gels (1610175, Bio-Rad) via electrophoresis at 150 V, and transferred onto 0.2 μm PVDF membranes. Membranes were blocked with 5% nonfat milk in Tris-Buffered Saline with Tween 20 (TBST) and incubated overnight at 4°C with the specified primary antibodies: (Supplemental Table 30). After washing, membranes were incubated with appropriate horseradish peroxidase-conjugated secondary antibodies for 1 hour at room temperature. Protein bands were visualized using the ChemiDoc Imaging System (Bio-Rad), and band intensities were quantified using ImageJ software, with β-actin serving as the loading control. The source/vendor and catalog number for all commercial primary and secondary antibodies used in the study are provided in Supplemental Table 30.

RNA isolation and real-time PCR

Cells were lysed in QIAzol Lysis Reagent (79306, Qiagen), and total RNA was extracted using the RNeasy Kit (74004, Qiagen). Complementary DNA (cDNA) was synthesized using the High-Capacity cDNA Reverse Transcription Kit (4368813, Thermo Fisher Scientific). Quantitative real-time PCR (qRT-PCR) was performed using an Applied Biosystems QuantStudio™ 6 Flex Real-Time PCR System.

Mouse models

Animal studies were approved by the Division of Laboratory Animal Resources and the Institutional Review Board at the University of Pittsburgh. C57Bl6 *Ncoa7* knockout (*Ncoa7*$^{-/-}$) mice (C57BL/6N-Ncoa7 tm1.1(KOMP)Vlcg/TcpMmucd) were obtained from the Mutant Mouse Resource & Research Centers (MMRRC). *Ncoa7*$^{-/-}$ mice were crossbred with interleukin-6 transgenic (*Il6* Tg) mice, an established inflammatory PH model (36), to generate the *Il6* Tg × *Ncoa7*$^{-/-}$ strain. Male mice were studied at 15–16 weeks of age.

IBA1 Z-stack confocal microscopy

Confocal images of Iba1+ stained mouse brain sections (40 µm thick) were captured using a Nikon A1 confocal microscope with a 40× lens. A Z-stack of 20 images was acquired at 2 µm intervals. For Iba1+ cell counting, only cells with a clearly identifiable soma or nucleus were included, while microglial cells lacking a visible soma—likely originating from adjacent tissue sections—were excluded. For each mouse, more than seven images were acquired in a blinded manner, and cell counts were performed separately in the molecular layer (ML) and granular layer (GL).

snRNA-seq analysis

Single nuclei were isolated from mouse brain tissues following a previously published method (37). Mouse cerebellar tissue was harvested and immediately dissected on ice. The tissue was transferred to a Dounce homogenizer containing 1 mL of Buffer D and homogenized with 10 strokes using pestle A followed by 10 strokes using pestle B. The homogenate was transferred to a 15 mL tube and mixed with an additional 1.5 mL of Buffer D and 2.3 mL of Buffer C by gentle inversion. For density-gradient purification, 1.5 mL of the homogenate mixture was carefully layered onto 340 µL of Buffer E in a 2 mL RNase-free microcentrifuge tube. Samples were centrifuged at 13,000 × g for 20 min at 4°C. Following centrifugation, the supernatant was carefully removed, and the resulting nuclei pellet was resuspended in 340 µL of Buffer D per tube. Nuclei suspensions from all tubes were pooled and

passed through a sterile 30 μm cell strainer to remove debris and aggregates. The filtered nuclei suspension was centrifuged at 700 × g for 4 min at 4°C. The supernatant was discarded, and the nuclei pellet was gently resuspended in Washing Buffer for subsequent single-nucleus RNA sequencing library preparation. Buffer C consisted of 50% OptiPrep, 10 mM Tris-HCl (pH 7.4), 10 mM NaCl, 5 mM $MgCl_2$, 0.1% Nonidet P-40 Substitute, 0.5 mM spermidine, 0.15 mM spermine, 1 mM DTT, 20 U/mL Superase-In RNase inhibitor, and EDTA-free protease inhibitor. Buffer D consisted of 0.25 M sucrose, 10 mM Tris-HCl (pH 7.4), 10 mM NaCl, 5 mM $MgCl_2$, 0.1% Nonidet P-40 Substitute, 0.5 mM spermidine, 0.15 mM spermine, 1 mM DTT, 20 U/mL RNasin ribonuclease inhibitor, 10 U/mL Superase-In RNase inhibitor, and EDTA-free protease inhibitor. Washing Buffer consisted of 1% bovine serum albumin (BSA), 1 mM DTT, 20 U/mL RNasin ribonuclease inhibitor, and 10 U/mL Superase-In RNase inhibitor prepared in RNase-free PBS.

Library preparation was performed according to the manufacturer's instructions (GEM-X Universal 3' Gene Expression v4 4-plex On-chip Multiplexing protocol, 10x Genomics). Briefly, nuclei from different samples were labeled with sample-specific OCM barcodes and pooled prior to GEM generation. The pooled nuclei were resuspended in the master mix and loaded together with partitioning oil and barcoded gel beads into the GEM-X chip to generate Gel Beads-in-Emulsion (GEMs). The poly(A) RNA from lysed nuclei contained within each GEM was reverse transcribed to cDNA containing a Cell Barcode and Unique Molecular Identifier (UMI). The pooled barcoded cDNA was then purified, amplified by PCR, and fragments of the appropriate size were selected using SPRI-based size selection for subsequent library construction. During library construction, the cDNA was fragmented and subjected to end repair, A-tailing, adaptor ligation, and PCR amplification to incorporate P5 and P7 sequences, i5 and i7 sample indices, and the TruSeq Read 2 primer sequence. Final libraries were sequenced on an Illumina platform.

The snRNA-seq data processing and analyses were performed using Seurat (38) across WT, *Il6* Tg (PH), and *Il6* Tg;*Ncoa7-/-* (PH_KO) conditions. Initial quality control retained nuclei with 1,200–7,000 detected features, 3,000–30,000 total counts, and ≤5% mitochondrial RNA. Doublets were identified separately for each sample using *scDblFinder* after normalization, variable-feature selection, scaling, PCA, nearest-neighbor graph construction, and clustering; nuclei classified as singlets were retained for downstream analysis. After doublet removal and final quality control, 248,660 nuclear profiles were retained, with a median capture of 2,301 unique features per nucleus. These included 69,197 WT, 74,101 *Il6* Tg, and 105,362 *Il6* Tg; *Ncoa7-/-* nuclei, with minimal inter-individual batch effects. Cell identities were assigned by reference-based label transfer in Seurat using an annotated mouse cerebellum snRNA-seq atlas (39). Briefly, anchors between the reference atlas and query dataset were identified using *FindTransferAnchors* with log-normalized RNA expression, PCA-based reference reduction, and the first 30 dimensions, followed by label transfer using *TransferData*.

To ensure conservative annotation, transferred labels were evaluated at the unsupervised cluster level across multiple clustering resolutions. Final annotations were assigned only when the dominant transferred label accounted for ≥90% of nuclei within a cluster. Clusters not meeting this criterion were subsetted and reclustered, and subcluster-level labels were retained only when the dominant transferred label accounted for ≥90% of nuclei. Nuclei from clusters or subclusters not meeting this purity criterion were left unassigned and excluded from cell-type-specific downstream analyses. Annotated cell types included granule neurons, molecular layer interneurons (MLIs), Purkinje cells, Golgi cells, unipolar brush cells (UBCs), Bergmann glia, astrocytes, oligodendrocytes, OPCs, microglia, macrophages, endothelial cells, ependymal cells, choroid plexus cells, Purkinje layer interneurons (PLIs), and fibroblasts.

Purkinje cells were recovered at lower numbers than PLIs and granule neurons, consistent with known technical and biological features of cerebellar snRNA-seq. Purkinje nuclei are large and fragile and are

more susceptible to mechanical lysis during nuclei isolation (40, 41), and prior work has noted that standard snRNA-seq protocols are biased against Purkinje cell recovery unless specialized nuclei-isolation conditions are used (42). In addition, granule neurons are the most abundant neuronal population in the cerebellum and greatly outnumber Purkinje cells (43, 44).

Cell-type-specific differential expression analysis was performed using the Wilcoxon rank-sum test (adjusted P-value ≤ 0.05). Pathway enrichment on DEGs was performed using EnrichR Gene Ontology Biological Process (GOBP) and KEGG analyses (22). Pathways supported by fewer than three genes were excluded, and FDR adjustment was recalculated across the remaining pathways. Enriched pathways were reported using an adjusted P-value cutoff of 0.05.

Spatial transcriptomics generation

Spatial transcriptomics was performed on 10-μm fresh-frozen mouse cerebellar sections using the 10x Genomics Visium CytAssist workflow according to manufacturer protocols. Sections were methanol-fixed, H&E-stained, imaged, destained, transferred to Visium slides, and processed for library preparation and Illumina sequencing using the GEM-X Universal 3′ Gene Expression v4 4-plex on-chip multiplexing workflow.

Raw sequencing data were processed using 10x Genomics Space Ranger to perform demultiplexing, read alignment, UMI counting, tissue-image alignment, spot calling, and generation of spatial gene-expression matrices. Spatial spots with fewer than 200 detected features or fewer than 500 counts were excluded. Spatial transcriptomic data from nine cerebellar samples (5 WT, 5 PH, 3 PH_NCOA7-KO) were then analyzed in Seurat. All spatial spots were first jointly integrated to establish a common latent space across slides and conditions. Cerebellar regions were manually delineated using anatomical location, extracted, and re-integrated to improve resolution of cerebellar layer- and region-specific transcriptional patterns. Unsupervised graph-based clustering was performed at resolution

0.2. Cell-type composition was inferred using Robust Cell Type Decomposition (RCTD) (45) with the matched cerebellar snRNA-seq reference. For final deconvolution, Purkinje layer interneurons (PLIs) were excluded because their strongest inferred enrichment occurred in a fourth ventricle–adjacent cluster rather than the Purkinje cell layer, suggesting anatomically implausible cross-mapping or ambient RNA contribution from this rare reference population. Macrophages were also excluded from fraction inference because of their low abundance and close transcriptomic similarity to microglia, which could otherwise confound estimation of microglia-associated spatial signals. The resulting deconvolution resolved major cerebellar neuronal, glial, ventricular, vascular, and stromal signatures across spatial clusters.

Spatial domain- and cell-type–targeted differential expression analyses were performed using the integrated Visium Seurat object with RCTD-derived cell-type fractions. For each spatial cluster and pairwise comparison (*Il6* Tg vs WT, and *Il6* Tg; *Ncoa7-/-* vs *Il6* Tg), differential expression was tested using MAST on normalized spatial expression values. To identify transcriptional programs associated with the principal enriched cellular/anatomical signature of each spatial domain, the dominant RCTD fraction for that cluster was treated as the target signal and therefore excluded from covariate adjustment to avoid over-adjustment of the biological compartment of interest. Non-dominant RCTD cell-type fractions with sufficient abundance and variability were included as latent variables to reduce confounding from neighboring or admixed cell populations within each spatial spot. In parallel, microglia-targeted analyses were performed within each spatial cluster by treating the RCTD microglia fraction as the target signal and adjusting for other available non-target cell-type fractions. Pathway enrichment was performed using EnrichR Gene Ontology Biological Process analysis (22). Enriched pathways were reported at FDR ≤ 0.05.

Supplemental References

1. Exposto F, et al. Burden of pulmonary arterial hypertension in England: retrospective HES database analysis. *Ther Adv Respir Dis.* 2021;15:1753466621995040.
2. Gelzinis TA, et al. Pulmonary hypertension in 2021: part I—definition, classification, pathophysiology, and presentation. *J Cardiothorac Vasc Anesth.* 2022;36(6):1552–1564.
3. Link J, et al. International Classification of Diseases coding changes lead to profound declines in reported idiopathic pulmonary arterial hypertension mortality and hospitalizations: implications for database studies. *Chest.* 2011;139(3):497–504.
4. Al-Omary M, et al. Pulmonary hypertension due to left heart disease: diagnosis, pathophysiology, and therapy. *Hypertension.* 2020;75(6):1397–1408.
5. Kotronia E, et al. Oral health and all-cause, cardiovascular disease, and respiratory mortality in older people in the UK and USA. *Sci Rep.* 2021;11(1):16452.
6. Alfaro-Almagro F, et al. Image processing and quality control for the first 10,000 brain imaging datasets from UK Biobank. *Neuroimage.* 2018;166:400–424.
7. Smith SM, et al. Advances in functional and structural MR image analysis and implementation as FSL. *Neuroimage.* 2004;23(Suppl 1):S208–S219.
8. Jenkinson M, et al. Improved optimization for the robust and accurate linear registration and motion correction of brain images. *Neuroimage.* 2002;17(2):825–841.
9. Jenkinson M, Smith S. A global optimisation method for robust affine registration of brain images. *Med Image Anal.* 2001;5(2):143–156.
10. Fischl B. FreeSurfer. *Neuroimage.* 2012;62(2):774–781.
11. Beckmann CF, Smith SM. Probabilistic independent component analysis for functional magnetic resonance imaging. *IEEE Trans Med Imaging.* 2004;23(2):137–152.
12. Salimi-Khorshidi G, et al. Automatic denoising of functional MRI data: combining independent component analysis and hierarchical fusion of classifiers. *Neuroimage.* 2014;90:449–468.

13. Griffanti L, et al. ICA-based artefact removal and accelerated fMRI acquisition for improved resting state network imaging. *Neuroimage.* 2014;95:232–247.

14. Glasser MF, et al. The minimal preprocessing pipelines for the Human Connectome Project. *Neuroimage.* 2013;80:105–124.

15. Brazauskas R, Logan BR. Observational studies: matching or regression? *Biol Blood Marrow Transplant.* 2016;22(3):557–563.

16. Bender R, Lange S. Adjusting for multiple testing—when and how? *J Clin Epidemiol.* 2001;54(4):343–349.

17. Rothman KJ. No adjustments are needed for multiple comparisons. *Epidemiology.* 1990;1(1):43–46.

18. Perneger TV. What’s wrong with Bonferroni adjustments. *BMJ.* 1998;316(7139):1236–1238.

19. Althouse AD. Adjust for multiple comparisons? It’s not that simple. *Ann Thorac Surg.* 2016;101(5):1644–1645.

20. Xia M, et al. BrainNet Viewer: a network visualization tool for human brain connectomics. *PLoS One.* 2013;8(7):e68910.

21. Nestor LJ, et al. Cannabis-dependent adolescents show differences in global reward-associated network topology: a functional connectomics approach. *Addict Biol.* 2020;25(2):e12752.

22. Kuleshov MV, et al. Enrichr: a comprehensive gene set enrichment analysis web server 2016 update. *Nucleic Acids Res.* 2016;44(W1):W90–W97.

23. Watts D, et al. Hypoxia pathway proteins are master regulators of erythropoiesis. *Int J Mol Sci.* 2020;21(21):8131.

24. Denko NC, et al. Investigating hypoxic tumor physiology through gene expression patterns. *Oncogene.* 2003;22(37):5907–5914.

25. Liu Q, et al. Attenuating vascular stenosis-induced astrogliosis preserves white matter integrity and cognitive function. *J Neuroinflammation.* 2021;18(1):187.

26. Yushkevich PA, et al. User-guided 3D active contour segmentation of anatomical structures: significantly improved efficiency and reliability. *Neuroimage.* 2006;31(3):1116–1128.
27. Ma Y, et al. A three-dimensional digital atlas database of the adult C57BL/6J mouse brain by magnetic resonance microscopy. *Neuroscience.* 2005;135(4):1203–1215.
28. Ma Y, et al. In vivo 3D digital atlas database of the adult C57BL/6J mouse brain by magnetic resonance microscopy. *Front Neuroanat.* 2008;2:1.
29. Jenkinson M, et al. FSL. *Neuroimage.* 2012;62(2):782–790.
30. Avants BB, et al. A reproducible evaluation of ANTs similarity metric performance in brain image registration. *Neuroimage.* 2011;54(3):2033–2044.
31. Arndt S, et al. Problems with ratio and proportion measures of imaged cerebral structures. *Psychiatry Res.* 1991;40(1):79–89.
32. Sanfilipo MP, et al. Correction for intracranial volume in analysis of whole brain atrophy in multiple sclerosis: the proportion vs. residual method. *Neuroimage.* 2004;22(4):1732–1743.
33. Voevodskaya O, et al. The effects of intracranial volume adjustment approaches on multiple regional MRI volumes in healthy aging and Alzheimer's disease. *Front Aging Neurosci.* 2014;6:264.
34. Harvey LD, et al. Lysosomal dysfunction and inflammatory sterol metabolism in pulmonary arterial hypertension. *Science.* 2025;387(6732):eadn7277.
35. Matera A, et al. Protocol to assess engulfment and degradation of synaptosomes by murine microglia in vitro. *STAR Protoc.* 2025;6(3):103936.
36. Castroflorio E, et al. The Ncoa7 locus regulates V-ATPase formation and function, neurodevelopment and behaviour. *Cell Mol Life Sci.* 2021;78(7):3503–3524.
37. Kocabas AM, Marin-Valencia I. Protocol for enhancing RNA yield and quality from single nuclei isolated from mouse brain tissue. *STAR Protoc.* 2024;5(4):103495.
38. Butler A, et al. Integrating single-cell transcriptomic data across different conditions, technologies, and species. *Nat Biotechnol.* 2018;36(5):411–420.

39. Kozareva V, et al. A transcriptomic atlas of mouse cerebellar cortex comprehensively defines cell types. *Nature.* 2021;598(7879):214–219.

40. Bartelt LC, et al. Antibody-assisted selective isolation of Purkinje cell nuclei from mouse cerebellar tissue. *Cell Rep Methods.* 2024;4(7):100816.

41. Shen Y. Sequencing the landscape of cerebellar gene expression. *J Mol Cell Biol.* 2019;11(8):633–635.

42. Bartelt LC, et al. Dysregulation of zebrin-II cell subtypes in the cerebellum is a shared feature across polyglutamine ataxia mouse models and patients. *Sci Transl Med.* 2024;16(772):eadn5449.

43. Wagner MJ, Luo L. Neocortex-cerebellum circuits for cognitive processing. *Trends Neurosci.* 2020;43(1):42–54.

44. Becker EBE, et al. Generating cerebellar organoids from pluripotent stem cells. *Dis Model Mech.* 2026;19(1):dmm052478.

45. Cable DM, et al. Robust decomposition of cell type mixtures in spatial transcriptomics. *Nat Biotechnol.* 2022;40(4):517–526.
