# Supplementary material for: Cerebellar and frontal lobe alterations in pulmonary hypertension implicate an inflammatory lung vascular/brain axis
Source: J Clin Invest. 2026 Nov 2;136(21):e195110. doi: 10.1172/JCI195110 (PMC13722401; doi:10.1172/JCI195110)

Figure 10A

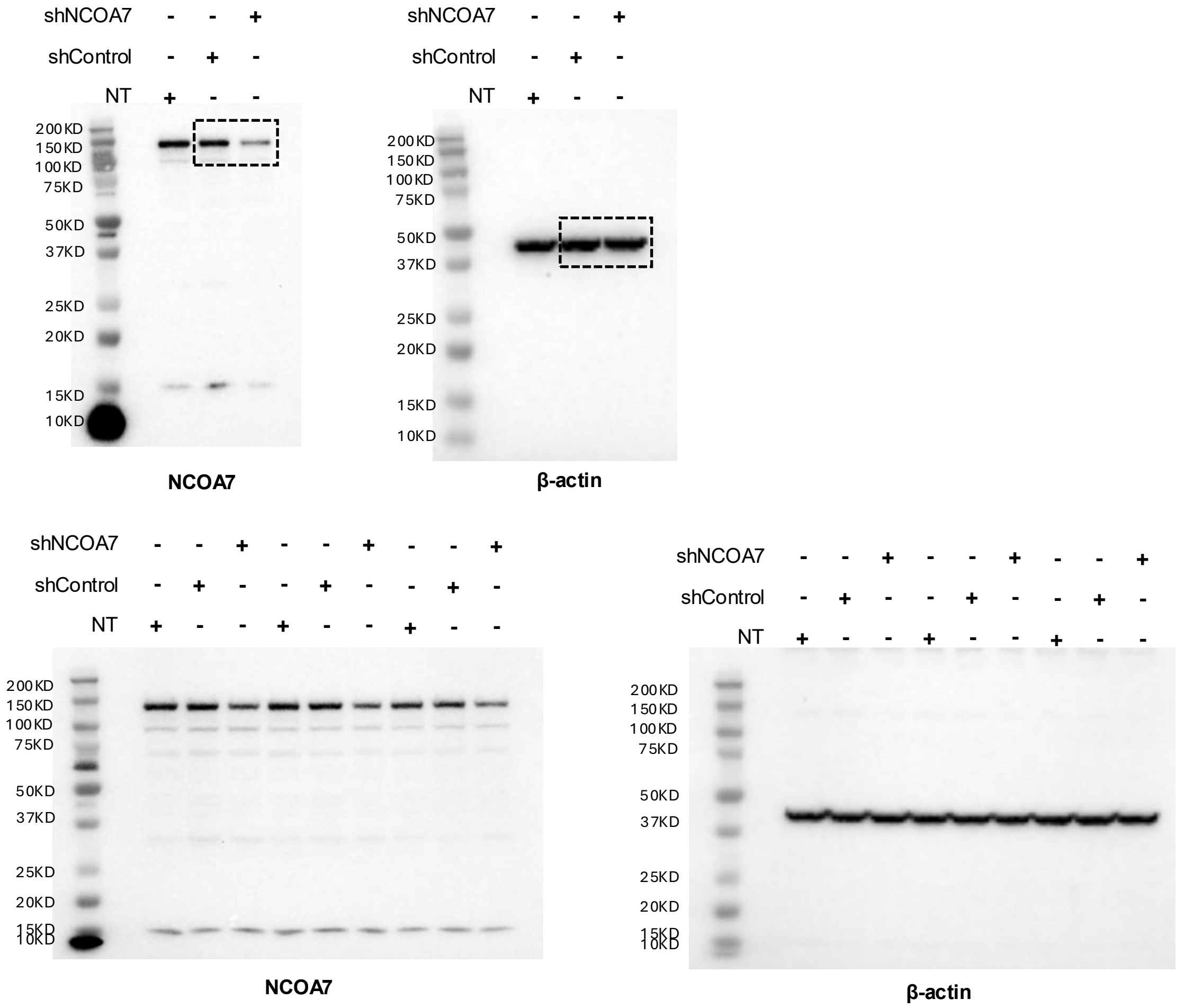

Figure S6A

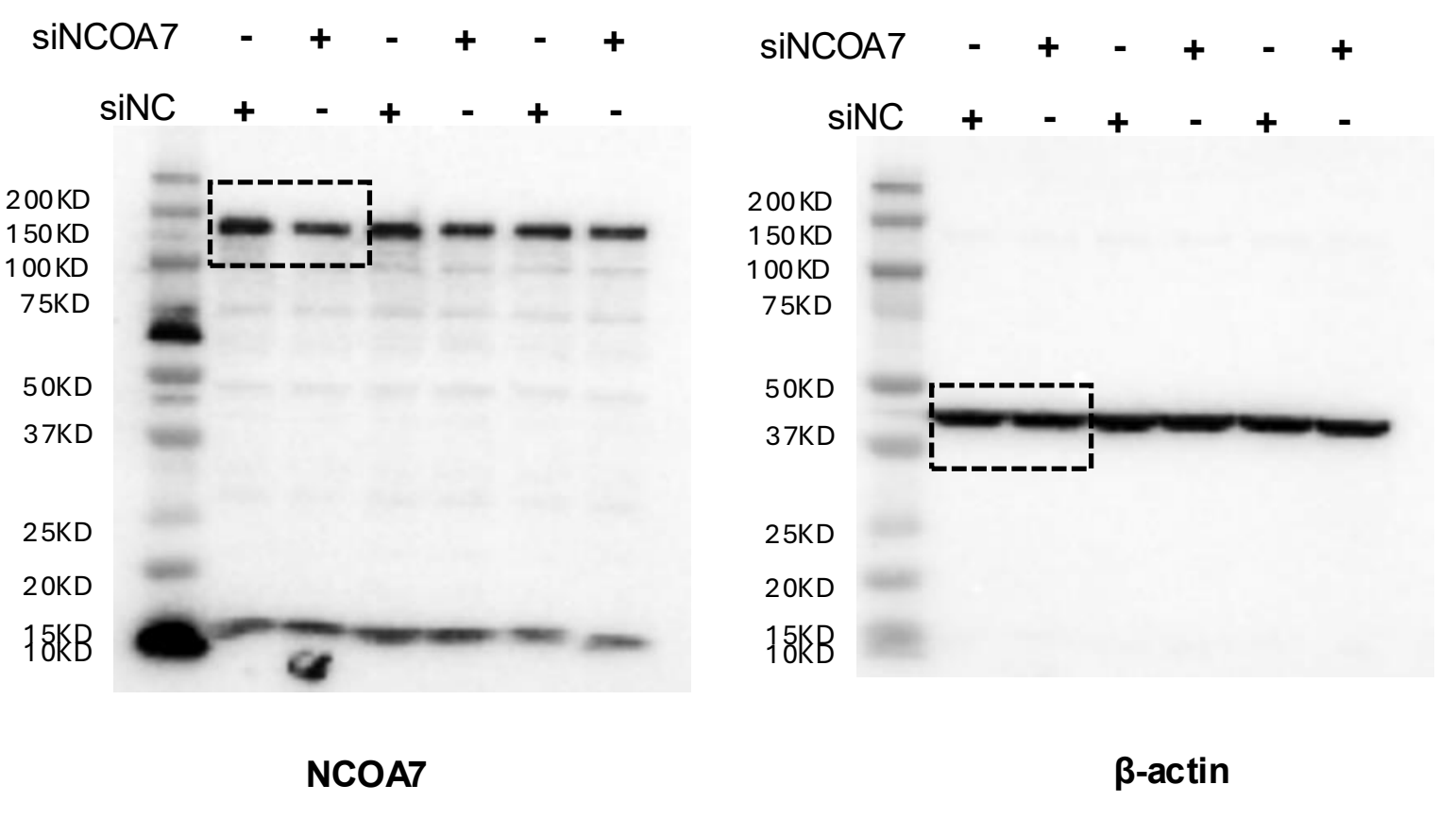

Supplement: Supplementary file 2 [file jci-136-195110-s032.pdf]
